# Application of the STAAR framework in detecting rare variant associations with Alzheimer disease and related dementias: Insights and implications

**DOI:** 10.1016/j.xhgg.2026.100574

**Published:** 2026-01-20

**Authors:** Dongyu Wang, Sabrina Abbruzzese, Nancy Heard-Costa, Andy Rampersaud, Eden Martin, Adam Naj, Bilcag Akgun, Brian Kunkle, Sudha Seshadri, Gina Peloso, The Alzheimer's Disease Neuroimaging Initiative, The Alzheimer's Disease Sequencing Project, Anita L. DeStefano, Zilin Li, Xihao Li, Seung Hoan Choi

**Affiliations:** 1 Department of Biostatistics, Boston University School of Public Health, Boston, MA 02118, USA; 2 Department of Neurology, Boston University Chobanian & Avedisian School of Medicine, Boston, MA 02118, USA; 3 NHLBI Framingham Heart Study, Framingham, MA 01702, USA; 4 Research Computing Services, Information Services & Technology, Boston University, Boston, MA 02215, USA; 5 John P. Hussman Institute for Human Genomics, University of Miami Miller School of Medicine, Miami, FL 33136, USA; 6 The Dr. John T. Macdonald Foundation Department of Human Genetics, University of Miami Miller School of Medicine, Miami, USA, Miami, FL 33136, USA; 7 Department of Biostatistics, Epidemiology, and Informatics, Perelman School of Medicine, Philadelphia, PA 19104, USA; 8 Penn Neurodegeneration Genomics Center, Department of Pathology and Laboratory Medicine, University of Pennsylvania Perelman School of Medicine, Philadelphia, PA 19104, USA; 9 Glenn Biggs Institute for Alzheimer's Disease and Neurodegenerative Diseases, University of Texas Health San Antonio, San Antonio, TX 78229, USA; 10 School of Mathematics and Statistics and KLAS, Northeast Normal University, Changchun, Jilin 130024, China; 11 Department of Biostatistics, University of North Carolina at Chapel Hill, Chapel Hill, NC 27599, USA; 12 Department of Genetics, University of North Carolina at Chapel Hill, Chapel Hill, NC 27599, USA

**Keywords:** rare variant analysis, STAAR framework, Alzheimer disease and related dementias, statistical refinement

## Abstract

Rare genetic variation is considered a potential source of heritability in individuals with sporadic Alzheimer disease and related dementias (ADRD). The Variant-set test for association using annotation information (STAAR) framework leverages multiple functional annotations of genetic variants and combines association statistics from multiple variant aggregation-based methods, including burden, sequence kernel association test (SKAT), and aggregated Cauchy association test (ACAT-V), into a single measure of significance. Using whole-genome sequencing data from the Alzheimer’s Disease Sequencing Project (ADSP), we comprehensively examined the association of rare genetic variation with ADRD in 23,454 individuals (37% individuals affected by ADRD) and with cognitively healthy elder status in 13,292 individuals (13% cognitively healthy elders) from diverse populations via the STAAR framework. We identified several genes significantly associated with ADRD or cognitively healthy status. However, our analysis revealed several limitations within the STAAR framework incorporating ultra-rare variants with dichotomous outcomes. To enhance the robustness of the framework, we proposed several computational refinements, including creating a burden of ultra-rare variants and employing more precise annotations to match the expected mechanism. After implementing the proposed modifications, the association with ADRD for *ZNF200* was no longer statistically significant (α = 1 × 10^−7^), while *TBX19*, *PLXNB2*, *CARD11*, and *LINC01880* remained significantly associated with cognitively healthy status. We identified and addressed the computational limitations in the STAAR framework that could lead to potential spurious results for ultra-rare variant aggregates with an extremely low cumulative minor-allele count. Our proposed refinements produced more robust results for associations with rare variants in the context of dichotomous outcomes.

## Teaser

In this study, we introduced a set of modifications to the STAAR framework to enhance its robustness in rare-gene-based analyses. The modified framework demonstrated improved statistical validity and consistency in identifying rare variant associations using whole-genome sequencing data from a large, diverse cohort.

## Introduction

Alzheimer disease (AD), which has a prevalence of 10.9% among people aged 65 and older in the United States,^1^ remains the primary cause of dementia.^2^ While Mendelian forms of AD account for only about 1% of affected individuals, the majority of instances are sporadic, highlighting the necessity of finding genetic variants that are associated with the disease.^3^ Previous twin studies estimated the heritability of AD to be about 70%, but the estimated SNP heritability was only 3.1% in genome-wide association studies (GWASs).^4^^,^^5^ The gap in heritability estimates between these studies can be attributed to many factors, of which rare genetic variation is a widely discussed potential source of the “missing heritability.”^6^ Many genetic studies focus on the broader phenotypic category of AD and related dementias (ADRD), which affect more than 55 million people worldwide.^7^ Bellenguez et al. performed a GWAS in 788,989 individuals with European ancestry and reported rare single-variant associations with ADRD in *TREM2*, *PLCG2*, and *ABI3* genes; it noted limitations such as insufficient statistical power due to difficulties in the identification of rare variants with very low frequencies (e.g., <0.01%).^8^ Recent advances in next-generation sequencing have enabled short- and long-read sequencing of the whole genome or exome for robust identification of rare variants.^9^^,^^10^^,^^11^ These new technologies have led researchers to focus on rare genetic variants in ADRD-related association studies. A whole-exome sequencing (WES) study with 17 AD-related traits found that rare coding variations in *RBKS* and *OR7A10* contribute to cognitive performance and protection against left hippocampal atrophy, respectively.^12^ Whole-genome sequencing (WGS) ADRD association studies with limited sample sizes have previously been performed to investigate rare variant associations, and these studies found suggestive associations with ADRD in *LAIR1*, *TREM2*, and *PSEN1* genes.^13^^,^^14^

Unlike common variants, rare variant analyses require testing an aggregation of variants to maintain statistical power, and methods have been developed to address this power issue.^15^^,^^16^^,^^17^ Variant-set test for association using annotation information (STAAR) is one of the latest approaches in tackling challenges in rare variant analysis.^18^^,^^19^ By integrating functional annotation via the Functional Annotation of Variants Online Resources (FAVOR),^20^ STAAR efficiently generates functionally interpretable gene-based association results.^18^ The key advantage of STAAR lies in its ability to aggregate results across annotation and test sets into a single measure of statistical significance. This makes it particularly valuable for sequencing data from diverse ancestries, where assumptions about rare variants are less stringent. As a result, the implementation of the STAAR framework has been well accepted to explore rare variant associations using WGS data.^21^^,^^22^^,^^23^ Large-scale sequencing studies such as the Trans-Omics for Precision Medicine (TOPMed) Program of the National Heart, Lung, and Blood Institute (NHLBI), the Alzheimer’s Disease Sequencing Project (ADSP), the All of Us Research Project (AoU), and the UK Biobank have generated vast genomic datasets, and the STAAR framework has been applied within each of these studies.^21^^,^^23^^,^^24^^,^^25^ Here, we implement our extensions into the STAAR framework in the context of ADRD using ADSP data, but the updated STAAR framework is relevant to researchers across a broad range of complex diseases and genomic resources.

The ADSP is a collaborative research effort aimed at identifying genetic risk and protective factors for ADRD. The project collects data from diverse individuals from family, case-control, and cohort studies.^26^ High-quality WGS data were obtained through a standardized variant-calling pipeline with extensive quality controls.^27^^,^^28^ In this study, we investigate rare variant associations with ADRD using the STAAR framework in the ADSP release 4 (R4) WGS data. Additionally, we examine potential protective rare genetic variations among cognitively healthy elders within the ADSP. As one of the studies to implement the STAAR analysis pipeline for rare variant association studies with a dichotomous trait, our findings are of substantial importance to understanding the benefits and limitations of this framework. Given the growing interest, our research will serve as a valuable resource for future application of the STAAR framework in studies of dichotomous traits.

## Material and methods

### Study participants

ADSP includes multiple cohorts, and jointly called WGS data have been generated. A detailed description of the dataset used in this study can be found in the supplemental information. Study participants provided written informed consent per each study’s institutional review board (IRB)-approved protocol. This study was conducted under a protocol approved by the Boston University IRB.

The R4 release of the ADSP data used in this study contains 36,361 WGS samples accessed from the National Institute on Aging Genetics of Alzheimer’s Disease Data Storage Site (NIAGADS) (NG00067.v10). Genotype-wide quality checks were performed by the Genomic Center for AD (GCAD). Sample-level quality measurements were performed by GCAD, and outliers were excluded by using the mean ± 10 standard deviation of the residual of the following measures: genotype missing rate, singleton rate, heterozygous/homozygous ratio, and transition/transversion ratio. Carriers of clinical variants in *APP*, *PSEN1*, and *PSEN2* were removed from the ADRD samples using the same approach as described in a previous study.^29^ Participants from the progressive supranuclear palsy (PSP) and corticobasal degeneration (CBD) cohorts were excluded from our analysis because they did not have ADRD information. To focus on late-onset ADRD and to reduce misclassification in controls, we excluded individuals younger than 55 years. After the exclusions, we have 23,454 samples available for analysis, with 8,697 individuals affected by ADRD and 14,757 control subjects. Utilizing phenotypic data from the Phenotype Harmonization Consortium (PHC), we were able to identify 1,784 cognitively healthy elders and 11,508 general control subjects within our ADSP sample.^30^ Multi-allelic variants were excluded from this study. Quality Control (QC) flags in the ADSP files were used in a filtering process to retain high-quality variants as detailed in the supplemental information.

### Phenotype determination

The ADSP leverages data from diverse cohorts, each employing independent criteria (autopsy, clinical diagnosis, neuropsychological testing, etc.) to define AD or dementia status. Phenotype data from case-control, family, and the Alzheimer’s Disease Neuroimaging Initiative (ADNI) studies were centrally harmonized and provided in three phenotype files. For participants in the ADSP case-control phenotype study, which includes longitudinal data, we defined individuals affected by ADRD as individuals with either prevalent or incident AD. Participants with a baseline ADRD-free status were included as affected individuals if they developed ADRD at a later age, while participants without a diagnosis of ADRD at any point were coded as control subjects. We also included cohorts that provided dementia status rather than AD, including the Longitudinal Aging Study in India-Diagnostic Assessment of Dementia (LASI-DAD) study,^31^ which used machine learning to diagnose some participants.^32^ In the ADSP family studies, ADRD status was defined in varying ways, including no dementia, definite AD, probable AD, possible AD, family-reported AD, other dementia, family-reported no dementia, and unknown. To harmonize ADRD status among the family studies, individuals with possible, probable, or definite AD were defined as affected individuals, while those individuals classified as no dementia were defined as control subjects. Participants with family-reported AD, family-reported no dementia, other dementia, and unknown were all recoded as missing for ADRD status and excluded. The ADNI cohort provides information on mild cognitive impairment (MCI) in addition to AD status. Individuals with a current diagnosis of MCI were excluded from the analysis. The PHC cohort, which curated longitudinal ADRD diagnosis data across multiple ADSP cohorts, enabled the identification of cognitively healthy individuals in the R4 WGS data. Cognitively healthy status was defined as having a PHC diagnosis of no dementia with an age last cognitively normal above 85 or a diagnosis of AD or MCI after age 85. General control subjects were defined as individuals diagnosed with AD or MCI before age 85, individuals diagnosed with AD or MCI with missing age of onset, or individuals who were disease free but younger than age 85.

### Variant annotation

Rare genetic variants were annotated using the whole-genome functional annotation tool FAVORannotator, which automatically defines gene-based genetic variant sets according to their functional annotation.^20^ The FAVORannotator classifies variants into three main categories: coding, non-coding, and non-coding RNA (ncRNA) variants. Coding variants were further categorized into predicted function groups, including putative loss of function (pLoF), disruptive missense (DS), pLoF and DS, missense, and synonymous. Non-coding variants were mapped with information on untranslated regions (UTRs), downstream, upstream, promoters (CAGE and DHS), and enhancers (CAGE and DHS). Variant-level functional scores used principal-component analysis (PCA) to generate multi-dimensional annotation scores. A total of nine annotation principal component (aPC) scores and 3 integrative scores (CADD, LINSIGHT, and FATHMM-XF) were included in the set of annotations as standard output of the FAVORannotator.

### PCA and genetic kinship matrix

To account for population structure, we estimated PCs using PC-AiR in the GENESIS package.^33^^,^^34^ Because PC-AiR calculates ancestry-informative PCs while minimizing the influence of close relatives, this approach was adopted in this dataset, which included a diverse population, ensuring accurate global ancestry inference. For PC calculation, we selected variants with a minor-allele frequency (MAF) of >5%, a call rate of >99%, a GCAD-provided variant flag (VFlag) of 0, and a Robust Unified Hardy-Weinberg Equilbrium (HWE)^35^ *p* value > 10^−4^ and in low linkage disequilibrium (LD) regions (r^2^ < 0.1). Variants in regions of long-range and high LD were also removed from the datasets before PC calculations.^36^

Since the ADSP includes related participants, we estimated empirical relationships among individuals. PC-Relate, a tool for estimating kinship coefficients, identity-by-descent sharing probabilities, and inbreeding coefficients using genomic data, was applied to construct the genetic kinship matrix.^37^ A sparse kinship matrix was generated using the cutoff of fourth-degree relatives (0.022) for computation efficiency. This sparse genetic kinship matrix was incorporated into a generalized mixed-effects model for rare variant analyses.

### Rare variant analysis

We conducted analyses using the STAAR framework to perform association tests between rare variants and ADRD status.^18^^,^^19^^,^^38^ *APOE* genotypes were obtained from array genotyping data provided by participating cohorts. For participants without directly genotyped *APOE* data, *APOE* genotypes were determined from the WGS data. Variants with a MAF of less than 1% were included in the analysis. Different models were fit for ADRD and cognitively healthy status to ensure model fitness and statistical power. For the ADRD analysis, we used a logistic mixed-effects model, adjusting for sex, technical sequencing variables (sequencing center and PCR status), *APOE ε2* and *ε4* allele counts, and PCs associated with ADRD status. In the cognitively healthy individual analysis, we adjusted for technical sequencing variables, *APOE ε2* and *ε4* allele counts, and PCs associated with cognitively healthy status. Relatedness among individuals was adjusted using a genetic kinship matrix.

We set the significance threshold of the rare variant analysis to 1 × 10^−7^ based on Bonferroni correction for ∼20,000 genes across the categories. The STAAR-O *p* value was used to aggregate *p* values from the annotation sets for each gene category, and gene-based aggregates with a STAAR-O *p* value less than the significance threshold were considered statistically significant. Gene categories with a cumulative minor-allele count (cMAC) less than 10 were excluded from the results.^39^

### STAARpipeline modifications

**Figure 1:**
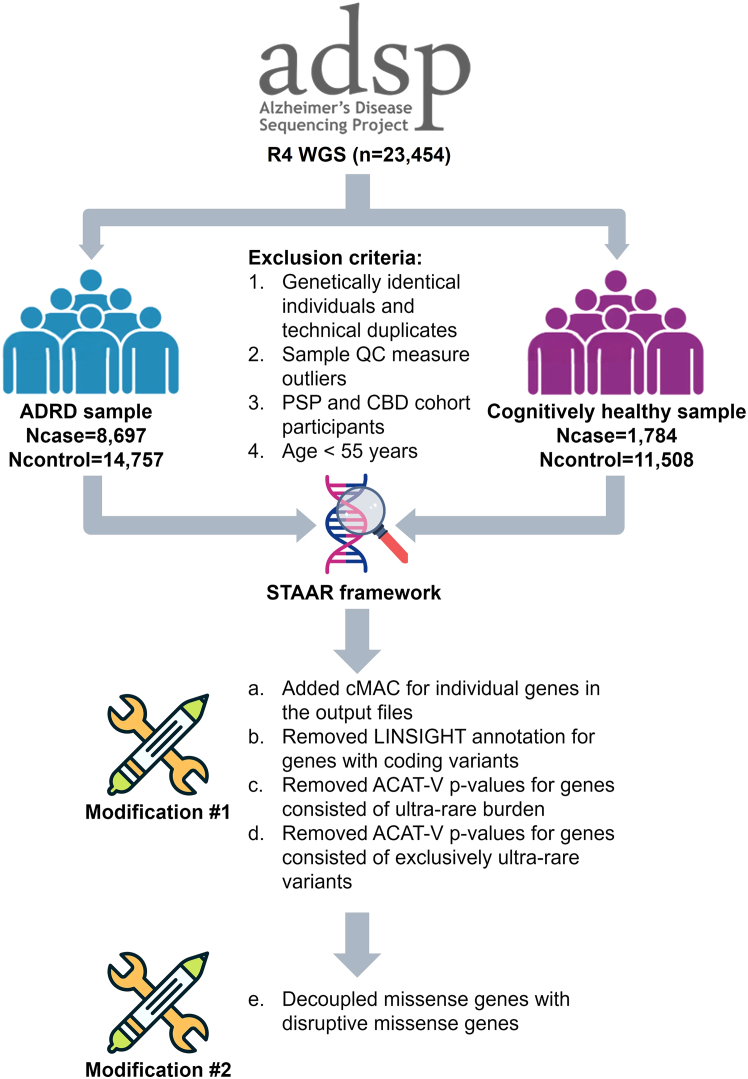
Flow chart of the analysis A schematic of the analysis. The ADSP R4 WGS sample used in this analysis contains 23,454 participants. Rare variant analyses were conducted using the STAAR framework in the R4 WGS samples for Alzheimer disease and related dementias (ADRD) status and a subset of the R4 WGS samples (*n* = 13,292) for cognitively healthy status. We performed two sensitivity analyses after implementing modifications to the STAAR framework in both samples and reported our findings from these analyses. cMAC, cumulative minor-allele count; PSP, progressive supranuclear palsy; CBD, corticobasal degeneration.

To enhance the robustness of the STAAR framework, we modified the STAARpipeline R package by (1) adding the cMAC for each gene aggregate in the output summary files; (2) removing LINSIGHT annotation for coding genetic variants; (3) removing the aggregated Cauchy association test (ACAT-V) *p* values in generating the STAAR-O *p* value when gene-based aggregates were formed from ultra-rare variants (MAC < 10), with the cMAC of these ultra-rare variants being less than 10; (4) removing the ACAT-V *p* values when the cMAC of the ultra-rare variants was equal to the cMAC of all variants in the gene; and (5) decoupling the missense and DS *p* values when generating the STAAR-O *p* values for missense genes. Sensitivity analyses were conducted using the modified STAARpipeline R package, with a suggestive significance threshold set at 1 × 10^−6^. All statistical analyses were performed using R 4.2.2. A schematic of the analysis is shown in Figure 1.

## Results

### Characteristics of participants

Our analysis sample of the ADSP WGS data contained 23,454 participants. Among the individuals affected by ADRD (*n* = 8,697, 37%), the mean baseline age was 75.6 years (SD = 8.6), while ADRD control subjects (*n* = 14,757, 63%) were slightly younger, with a mean baseline age of 74.3 years (SD = 8.9). We observed more females compared to males in both individuals affected by ADRD and control subjects (Table 1). Between the two *APOE* allele types that we modeled, *ε4* was more prevalent in both individuals affected by ADRD and control subjects, specifically, 51.3% of individuals affected by ADRD carrying at least one *ε4* allele as compared to 24.6% of the control subjects. As expected, more carriers of the *ε2* allele were observed in the ADRD control subjects, with 12.3% of the control subjects vs. 7.7% of the affected individuals carrying at least one *ε2* allele (Table 1).

**Table 1:** Descriptive statistics of the ADSP sample

	ADRD sample (*N* = 23,454)		Cognitively healthy sample (*N* = 13,292)	
	ADRD (*n* = 8,697)	Control (*n* = 14,757)	Cognitively healthy (*n* = 1,784)	General control (*n* = 11,508)
Baseline age (years)	75.6 (8.6)	74.3 (8.9)	82.5 (5.3)	71.7 (8.6)

**Sex**				

Female	5,426 (62.4%)	9,329 (63.2%)	1,151 (64.5%)	6,929 (60.2%)
Male	3,271 (37.6%)	5,428 (36.8%)	633 (35.5%)	4,579 (39.8%)

***APOE ε2***				

0	8,025 (92.3%)	12,946 (87.7%)	1,501 (84.1%)	10,494 (91.2%)
1	652 (7.5%)	1,736 (11.8%)	271 (15.2%)	984 (8.6%)
2	20 (0.2%)	75 (0.5%)	12 (0.7%)	30 (0.3%)

***APOE ε4***				

0	4,229 (48.6%)	11,119 (75.3%)	1,442 (80.8%)	6,276 (54.5%)
1	3,709 (42.6%)	3,386 (22.9%)	330 (18.5%)	4,288 (37.3%)
2	759 (8.7%)	252 (1.7%)	12 (0.7%)	944 (8.2%)

Genotyped *APOE* allele counts are reported.

For the cognitively healthy sample (*n* = 13,292), individuals with a cognitively healthy status were generally older at baseline and comprised more females and *APOE ε2* carriers as compared to the general control subjects (Table 1). Separate descriptive statistics, including cognitively normal age and age of onset for the cognitively healthy sample, are reported in Table S1. Based on the reported race and ethnicity provided in the ADSP data, our study sample included 2,085 Hispanic White, 209 Hispanic Black, 6,373 Hispanic other/unknown, 7,663 non-Hispanic White, 4,317 non-Hispanic Black, and 2,807 non-Hispanic other/unknown individuals (see supplemental information for details). Using a MAF threshold of 1%, we found 244.6 million bi-allelic variants for the subsequent gene-based tests.

### Aggregates of rare variants that are associated with ADRD or cognitively healthy status from the original STAAR framework

**Figure 2:**
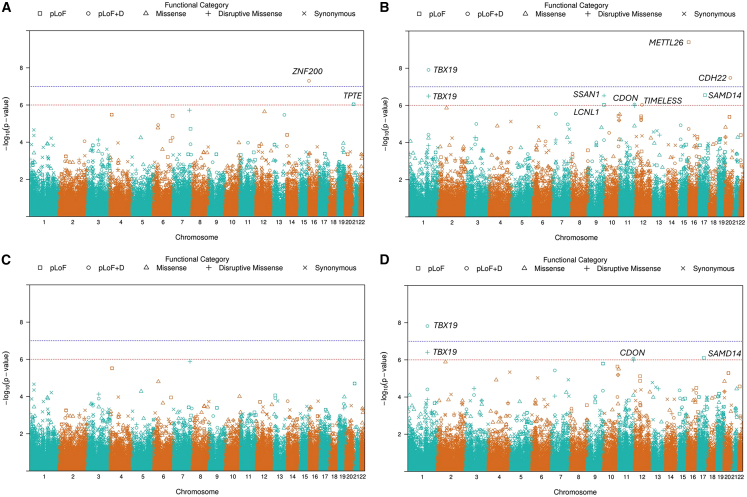
Manhattan plots of gene-based tests for coding variants in the ADRD and cognitively healthy samples before and after implementing modification #2 Manhattan plots show gene-based test results for coding variants. The blue reference line represents the genome-wide significance threshold of 1 × 10^−7^, and the red reference line represents a suggestively significant threshold of 1 × 10^−6^. (A and C) Gene-based test results before and after applying modification #2 in the ADRD sample. *TPTE* was annotated as both a pLoF and DS and a pLoF gene in (A). (B and D) Gene-based test results before and after applying modification #2 in the cognitively healthy sample. The *y* axis shows the −log10(*p* value), while the *x* axis represents the location of genes on chromosomes.

Applying the original STAAR framework, we identified 2 genes associated with ADRD status (Table 2; Figures 2A and S1). After adjusting for *APOE*, we observed that pLoF and DS variants in *ZNF200* showed genome-wide statistically significant association with ADRD (*p* = 5.05 × 10^−8^). Non-coding variants in *HLA-F* have shown suggestively significant association with ADRD (Table S2). In the cognitively healthy sample, we found statistically significant associations between cognitively healthy status and *METTL26*, *TBX19*, and *CDH22* genes (Table 2; Figure 2B). A few genes of non-coding rare variants and ncRNA also showed significant association with cognitively healthy status, such as *PLXNB2*, *CARD11*, and *LINC01880* (Table S2). However, the functional roles of these genes remain unclear, with limited evidence linking them directly to cognitive measures. Additionally, genes with suggestively significant associations (*p* < 1 × 10^−6^) are listed in Tables 2 and S2 as well.

**Table 2:** Gene-based test results for coding variants in the analyses

Gene	Chr	Category	No. of SNVs	cMAC	STAAR-O
**ADRD sample**					

*ZNF200*	16	pLoF and DS	7	25	5.05 × 10^−8^
*TPTE*	21	pLoF and DS	4	102	9.05 × 10^−7^

**Cognitively healthy sample**					

*METTL26*	16	pLoF	9	15	3.95 × 10^−10^
*SAMD14*	17	pLoF	2	98	2.85 × 10^−7^
*TBX19*	1	pLoF and DS	17	22	1.24 × 10^−8^
*LCNL1*	9	pLoF and DS	2	11	9.37 × 10^−7^
*CDON*	11	pLoF and DS	22	45	8.99 × 10^−7^
*TIMELESS*	12	pLoF and DS	9	11	9.29 × 10^−7^
*CDH22*	20	pLoF and DS	11	12	3.32 × 10^−8^
*TBX19*	1	DS	14	18	3.16 × 10^−7^
*SSNA1*	9	DS	3	14	3.06 × 10^−7^

Genes with significant or suggestive association with ADRD or cognitively healthy status are shown. Chr, chromosome; SNV, single-nucleotide variant; cMAC, cumulative minor-allele count; pLoF, putative loss of function; DS, disruptive missense.

### ACAT-V may be sensitive to ultra-rare variant sets

**Figure 3:**
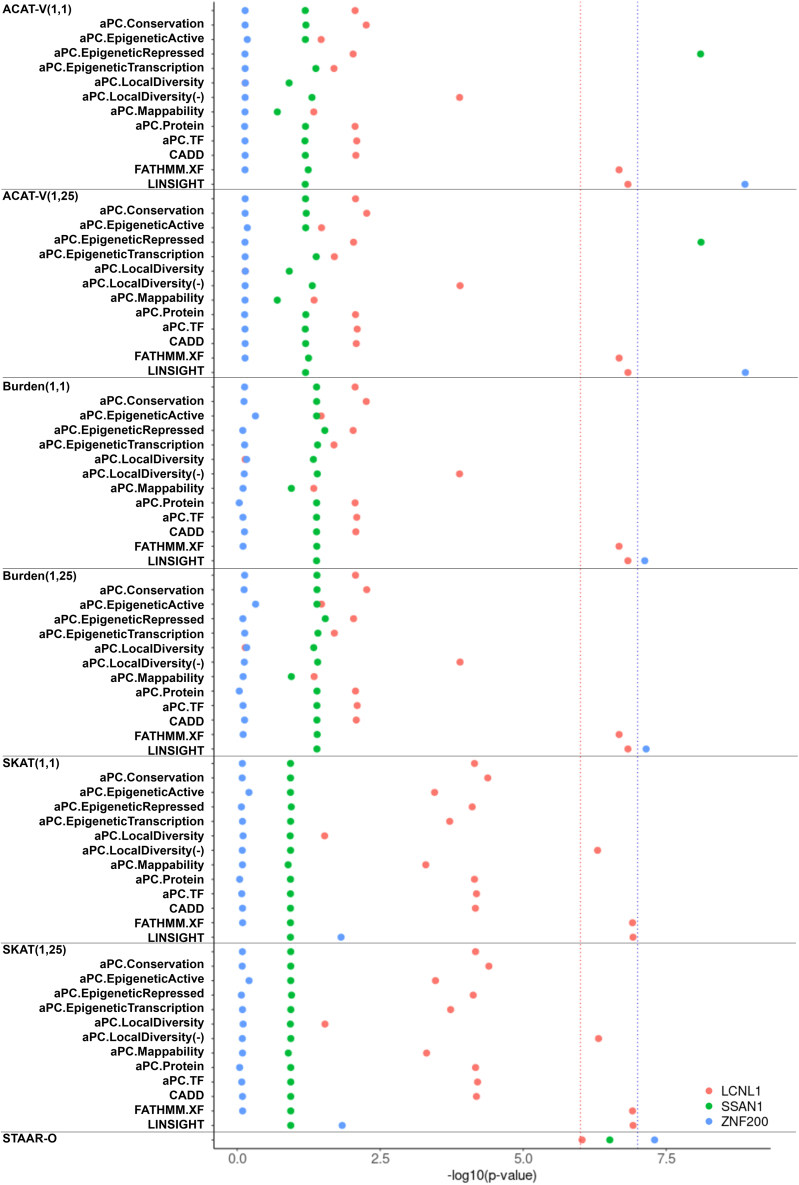
Annotation set *p* value plots for selected genes Scatterplots showing the annotation set *p* values of ACAT-V, burden, and SKAT for *SSAN1*, *LCNL1*, and *ZNF200*. The blue reference line represents the genome-wide significance threshold of 1 × 10^−7^, and the red reference line shows the suggestively significant threshold of 1 × 10^−6^. Color dots demonstrate *LCNL1* (red), *SSAN1* (green), and *ZNF200* (blue) *p* values, respectively. The *x* axis shows the −log10(*p* value), while the *y* axis represents different annotation sets.

The STAAR framework performs statistical analyses of annotation sets of rare variants using three main approaches: the burden test, the sequence kernel association test (SKAT), and the ACAT-V.^19^ ACAT-V has been shown to be robust to the sparsity of causal variants, the directionality of effects, and the choice of weights.^40^ With the significant associations found from our analyses, we next examined which individual annotation test set was driving the overall significance. To understand the exact functional annotation behind the significant signal, we visualized the *p* values from the individual annotation sets for the significant genes (Figure 3). The suggestive significance of *SSAN1*, which is a DS aggregate that includes 3 SNVs totaling 14 alleles, was solely driven by the ACAT-V aPC-conservation set *p* values. Further investigation revealed that only one of the SNPs (rs374374337) was highly significant in the single-variant analysis (Table S3), with only a single alternative allele carrier. ACAT-V integrated single-variant-analysis summary statistics for variants with a MAC of ≥10 and burden test summary statistics for variants with a MAC of <10. However, the burden test result may be sensitive to the low cMAC of *SSAN1* ultra-rare variants (MAC < 10), as the suggestive significance of *SSAN1* is primarily driven by one singleton in cognitively healthy individuals.

Another ACAT-V issue was observed in *LCNL1*, which consists of 2 SNPs totaling 11 alleles (Figure 3). Identical ACAT-V and burden *p* values were obtained for *LCNL1*. This is expected, as the ACAT-V test utilizes the burden test *p* value in its calculation due to the low allele count of *LCNL1* variants. When all variants in an aggregate have a MAC of <10, the cMAC of this gene equals the cMAC of its ultra-rare variants (MAC < 10), causing ACAT-V to generate the same *p* values as the burden test. This redundancy effectively doubles the weighting in the Cauchy approximation process, leading to less accurate results. To improve the robustness of ACAT-V, we applied multiple modifications in the existing STAARpipeline R package, including (1) removing the ACAT-V *p* values from the STAAR-O *p* value calculation when gene-based aggregates were composed of ultra-rare variants (MAC < 10) with a cMAC of <10 and (2) removing the ACAT-V *p* values when the cMAC of the ultra-rare variants was equal to the cMAC of all variants in the gene. Sensitivity analyses were performed to evaluate the impact of these modifications.

### LINSIGHT annotation in coding rare variants

Using the *p* value plot, we also examined the significance of individual annotation sets for the *ZNF200* pLoF and DS aggregate (Figure 3). Interestingly, only the LINSIGHT annotation set presented significant signals for *ZNF200*. LINSIGHT is a predictive measure of negative selection at known non-coding sites with inherited diseases, and the LINSIGHT score can accurately predict disease-associated genetic variants located outside protein-coding genes.^41^ However, we did not expect LINSIGHT-based set *p* values to drive the significance in known coding variants in *ZNF200* (Table S4). To ensure the accuracy of the rare variant annotation, we removed LINSIGHT annotation sets from all rare coding variant tests in the sensitivity analyses.

### Decoupling the DS aggregate from the missense category

**Figure 4:**
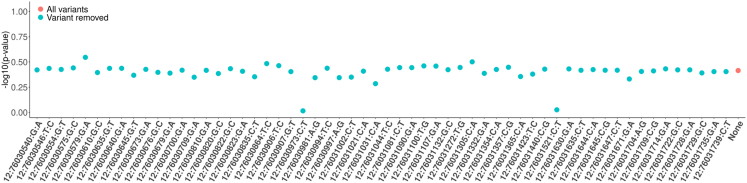
Leave-one-variant-out analysis for the *PHLDA1* missense aggregate Scatterplot demonstrates the STAAR-O *p*-values in the leave-one-variant-out analysis for the *PHLDA1* missense aggregate. Each variant was removed in the aggregate, and we performed 59 individual tests after implementing modification #1 in the ADRD sample. The red dot shows the *p* value without removing any variants in the aggregate. Blue dots represent the *p* values in the leave-one-out analysis. The *y* axis shows the −log10(*p* value), while the *x* axis represents the exact variant removed in each tested aggregate.

After implementing the modifications for ACAT-V and LINSIGHT, we noticed that the *PHLDA1* missense rare variant set has suggestive significance with ADRD (Figures S2 and S3). To determine the putative causal variant(s) within the *PHLDA1* missense aggregate, we performed a leave-one-variant-out analysis to identify the most impactful variant in *PHLDA1*. Surprisingly, none of the 58 tests showed a significant association with ADRD (Figure 4), despite suggestively significant STAAR-O *p* values observed in the Manhattan plot (Figure S2). After further investigation, we found that the STAAR function combines missense STAAR-O *p* value results with DS set *p* values for the same gene. The significance of *PHLDA1* missense aggregate was entirely driven by two ultra-rare DS variants (chr12:76030981A>G and rs551935657), which were already included in the missense aggregate set. Additionally, the cMAC of these two variants is less than 10, indicating that the significance of the *PHLDA1* missense aggregate may be inconclusive. To address this issue, we decoupled the missense and DS results in the STAAR-O *p* value calculation, keeping the results separated by the predicted variant function categories.

### Sensitivity analyses

Overall, we implemented multiple modifications to the existing STAAR and STAARpipeline R packages to enhance analytical robustness: we (1) added the cMAC for each gene aggregate in the output summary files; (2) removed LINSIGHT annotation for coding genetic variants; (3) removed the ACAT-V *p* values from STAAR-O *p* value calculation when gene-based aggregates were formed from ultra-rare variants (MAC < 10) with a cMAC of <10; (4) removed ACAT-V *p* values when the cMAC of the ultra-rare variants was equal to the cMAC of all variants in the gene; and (5) decoupled the missense and DS *p* values when generating the STAAR-O *p* values for missense genes (Figure 1). Sensitivity analyses using the modified STAAR framework revealed that the previously significant coding variant aggregates were no longer significant in the ADRD analysis (Figures 2C and 2D).

In non-coding gene-based tests, however, the *HLA-F* upstream aggregate showed suggestive significance for association with ADRD (Figures S4C and S5; Table S5). *HLA-F* belongs to the major histocompatibility complex (MHC) region, and it requires meticulous interpretation due to the complexity of the region in genetic association studies. In the analysis of cognitively healthy individuals, *TBX19*, *PLXNB2*, *CARD11*, and *LINC01880* remained significantly associated with cognitively healthy status in the sensitivity analysis (Tables 3 and S5; Figure S4D). One family study reported that a *TBX19* gene mutation was found in patients with isolated adrenocorticotropic hormone (ACTH) deficiency, which causes cognitive impairment, particularly in the neonatal period.^42^ Our findings allude to the potential protective role for cognition function decline in these genes. Modifications effectively remedied the issues found in our results and improved the robustness of the STAAR framework.

**Table 3:** Gene-based test results for coding variants in the analyses after implementing modification #2

Gene	Chr	Category	No. of SNVs	cMAC	STAAR-O
**Cognitively healthy sample**					

*SAMD14*	17	pLoF	2	98	7.77 × 10^−7^
*TBX19*	1	pLoF and DS	17	22	1.53 × 10^−8^
*CDON*	11	pLoF and DS	22	45	8.53 × 10^−7^
*TBX19*	1	DS	14	18	3.91 × 10^−7^

Genes with significant or suggestive association with ADRD or cognitively healthy status are shown. Chr, chromosome; SNV, single-nucleotide variant; cMAC, cumulative minor-allele count; pLoF, putative loss of function; DS, disruptive missense.

## Discussion

Rare genetic variants play a key role in the underlying genetic mechanism of ADRD, yet evidence linking rare variants to ADRD remains limited. The recently published STAAR framework offers a powerful, scalable, and accessible approach for performing annotation-based omnibus tests in rare variant analysis. The ADSP is the largest genetic consortium with WGS data from diverse populations of individuals with ADRD status. Prior ADSP publications have identified and confirmed genetic associations with ADRD, but the evidence supporting associations with rare genetic variations is still limited.^13^^,^^14^ To address this gap in knowledge, we leveraged functional annotation in rare variant analysis by applying the STAAR framework to gene-based tests in the ADSP R4 WGS data. This approach identified a few biologically plausible gene targets for ADRD or cognitively healthy status using both coding and non-coding rare variant aggregates. However, some of these findings may be sensitive to certain characteristics of rare variant aggregates due to the limitations in the framework. To enhance statistical robustness, we proposed and implemented modifications to mitigate the issues.

One of the challenges in rare variant analysis is adequately combining different test results from widely used methods such as burden tests and SKAT. Assumptions of these tests often involve the choice of weights, effect sizes, and direction of causal variants, as well as the number of causal variants. SKAT-O was developed as an omnibus test that combines both burden tests and SKAT, offering greater robustness to varying directionality in effect sizes.^43^ However, the power of SKAT-O can be severely impacted by the sparsity of causal variants in the genome.^44^ Additionally, combining multiple tests with SKAT-O can be computationally expensive because of the correlation among the tests. In contrast, ACAT-V has some distinctive benefits. Unlike conventional tests, ACAT-V requires minimal assumptions and relies only on the *p* values, making it computationally efficient.^40^ Utilizing the approximation from a Cauchy distribution, ACAT-V does not require LD or a population reference panel to construct the set-level-based test statistics. Instead, ACAT-V treats *p* values as independent variables, due to a key property of the Cauchy distribution.^45^ Additionally, if the set-based tests were effectively controlled for genetic relatedness and/or population structure, ACAT-V results will retain the same adjustment in their calculation automatically.^40^

In our analyses, we observed unique features of the ACAT-V that had not been previously discussed. Notably, ACAT-V combines burden test *p* values for ultra-rare variants (MAC < 10) with single-variant-analysis *p* values for rare variants (MAC ≥ 10) into a single significance measure.^40^ The STAAR framework incorporated this method, but the results are reliable only when the cMACs of ultra-rare variants within a gene aggregate exceed 10. For example, the *SSAN1* pLoF and DS aggregate only consists of 2 singletons and 1 rare variant with 14 alleles. A burden test was not appropriate in this scenario because the cMAC of the two singletons is far below the commonly used cutoff of 10 (Table S3).^39^^,^^46^^,^^47^ Additionally, ACAT-V test sets can share identical *p* values with the burden test sets if the gene aggregate consisted exclusively of ultra-rare variants. This redundancy may inflate the statistical weights in the STAAR-O *p* value calculation, as the same set of *p* values contributes twice to the final statistical measure. As demonstrated by *LCNL1* in Figure 3, the suggestive significance disappeared after our modifications in sensitivity analyses (Table 3; Figure 2D). To address these issues, we implemented adequate filtering approaches in the STAAR framework. We installed a MAC filter and a cMAC filter for ultra-rare variants in each gene aggregate, which effectively removed the ACAT-V *p* values from the STAAR-O *p* value calculation when gene aggregates consisted of ultra-rare variants with a cMAC of <10. We also excluded ACAT-V *p* values when a gene aggregate consists solely of ultra-rare variants. With these modifications, we believe that the statistical robustness of the STAAR framework was improved.

LINSIGHT combines a generalized linear model for functional genomic data with a probabilistic evolution model to identify non-coding variants associated with inherited diseases.^41^ A higher score in LINSIGHT corresponds to a higher probability that the non-coding variants are constrained to mutations. A previous report showed that rs7304782, a variant in the first intron of the *MTRFR* region, may contribute to schizophrenia risk by regulating *OGFOD2* expression in human brain tissues.^48^ However, the highly significant LINSIGHT-annotated burden, SKAT, and ACAT *p* values for *ZNF200* appear inconsistent with the intended function of the LINSIGHT score (Figure 3). In addition, we could not find any published evidence suggestive of the LINSIGHT score in protein-coding variants. To address this discrepancy, we removed LINSIGHT annotation test sets from all coding variant aggregates when combining the individual test set results in the STAAR framework.

STAAR framework integrates the FAVOR annotation database for genome-wide variant information, including gene name, position, and function.^20^ Missense variants were defined by non-synonymous status from the GENCODE exonic category, while DS status was predicted using a meta-analytic support vector machine (MetaSVM) algorithm.^49^^,^^50^ A common strategy in rare variant analysis is to construct separate missense and DS variant aggregates for individual genes, and the STAAR framework adopted this approach. However, the calculation of STAAR-O *p* values for missense genes was based on the annotation set *p* values of both the missense and DS aggregates of the same gene. This was unexpected, as the DS variants were already included in the missense aggregates, leading to overlapping variants in both functional sets. For example, we observed a suggestively significant signal in *PHLDA1*, yet the leave-one-variant-out analysis showed that none of the aggregates reached the suggestive significance threshold (Figures S2 and 4). We believe that the missense gene-based results should be decoupled from the DS results in generating the STAAR-O *p* value for the same gene. After separating missense with DS results for *PHLDA1*, we no longer observed suggestive significance in the sensitivity analyses.

The main focus of this study is on introducing computational refinements in the STAAR framework to produce robust results in association analyses of rare variants for dichotomous traits. We demonstrated the need for and utility of these refinements by applying them to publicly available ADSP WGS data using two distinct dichotomous phenotypes: ADRD status and cognitively healthy elder status. We recognize some limitations of these phenotype definitions. The exclusion of individuals younger than 55 years of age reduces misclassification of controls but may also limit power to identify rare variants driving Mendelian and/or complex forms of this disease in the 639 individuals affected by ADRD among those younger than 55 years in the ADSP R4 WGS data. Other study designs that implement distinct age thresholds for affected individuals and control subjects may provide additional information regarding ADRD genetic architecture. However, given our methodological focus, we consider these analyses beyond the scope of the current work and are better suited to larger-sample-size datasets.

The STAAR framework was initially applied to quantitative traits such as blood lipid levels, inflammation biomarkers, height, and glycemic traits.^19^^,^^21^^,^^23^^,^^24^^,^^25^ To date, there have been limited studies investigating rare genetic variant associations with dichotomous traits. This study was based on the ADSP R4 WGS data, but we have also observed the same issues in other datasets.^51^ Hence, the modifications proposed here are likely to have widespread utility in the study of rare variants in association with dichotomous traits.

### Conclusion

We systematically evaluated the association of rare genetic variation with ADRD in 23,455 individuals using the ADSP R4 WGS data. After final analyses, we did not observe significant associations with ADRD. Additionally, we investigated potentially protective rare variants associated with cognitively healthy status using a subset of the ADSP R4 WGS data. We identified that *TBX19*, *PLXNB2*, *CARD11*, and *LINC01880* are significantly associated with a cognitively healthy status. Further replication studies, which are beyond the scope of this method-focused article, are needed to confirm these association findings. Our main finding is that when implementing the original STAAR framework, we observed potential spurious associations in multiple genes due to issues related to ACAT-V, LINSIGHT annotation, and other factors. To address these issues, we proposed modifications to mitigate their effects while maintaining statistical power and robustness. We recommend the use of this modified STAARpipeline for analysis of dichotomous traits.

## Data and code availability

ADSP WGS data (NG00067.v10) and PHC phenotypic data (NG00067.v12) are available through the NIAGADS (https://www.niagads.org/). The code generated during this study is available at GitHub: https://github.com/DanielDYWang/STAARpipeline_new.

## Acknowledgments

Nancy Heard-Costa is supported by grants 75N92025D00012 and U01AG058589. Eden Martin is supported by U01AG058654. Adam Naj is supported by U01AG058654, RF1AG060472, U54AG052427, U01AG032984, and U24AG041689. Brian W. Kunkle is supported by U01AG076482, U01AG066767, U01AG058654, U01AG057659, and U01AG062943. Gina M. Peloso reports grant support from U01AG058589. Xihao Li is supported by R01AG085581. Seung Hoan Choi, Anita L. DeStefano, and Dongyu Wang are supported by U01AG058589 and U01AG068221. See the supplemental information for details. Data used in preparation of this article were obtained from the ADNI database (adni.loni.usc.edu). As such, the investigators within the ADNI contributed to the design and implementation of ADNI and/or provided data but did not participate in the analysis or writing of this report. A complete listing of ADNI investigators can be found at http://adni.loni.usc.edu/wp-content/uploads/how_to_apply/ADNI_Acknowledgement_List.pdf.

## Declaration of interests

The authors declare no competing interests.

## Web resources

Modified STAARpipeline R package, https://github.com/DanielDYWang/STAARpipeline_new
STAARpipeline R package, https://github.com/xihaoli/STAARpipeline

## Supplemental information

Document S1. Figures S1–S5, Tables S1–S5, and supplemental material and methods

Document S2. Article plus supplemental information
