# Supplementary material for: Application of the STAAR framework in detecting rare variant associations with Alzheimer disease and related dementias: Insights and implications
Source: HGG Adv. 2026 Jan 20;7(2):100574. doi: 10.1016/j.xhgg.2026.100574 (PMC12934298; doi:10.1016/j.xhgg.2026.100574)
Supplement: Document S1. Figures S1–S5, Tables S1–S5, and supplemental material and methods [file mmc1.pdf]

HGGA, Volume 7

Supplemental information

Application of the STAAR framework in detecting rare variant associations with Alzheimer disease and related dementias: Insights and implications

Dongyu Wang, Sabrina Abbruzzese, Nancy Heard-Costa, Andy Rampersaud, Eden Martin, Adam Naj, Bilcag Akgun, Brian Kunkle, Sudha Seshadri, Gina Peloso, The Alzheimer's Disease Neuroimaging Initiative, The Alzheimer's Disease Sequencing Project, Anita L. DeStefano, Zilin Li, Xihao Li, and Seung Hoan Choi

Supplemental Information

Supplemental Methods

ADSP Sample Description

snd10000-Alzheimer’s Disease Sequencing Project (ADSP) Discovery

The initial phase of the ADSP research plan is called the Discovery Phase. Samples were selected from well-characterized study cohorts of individuals with or without an AD diagnosis and the presence or absence of known risk factor genes. The ADSP generated three sets of genome sequence data for these samples as part of the Discovery Phase: (1) WGS for 584 samples from 113 multiplex families (two or more affected per family), (2) Whole Exome Sequence (WES) for 5,096 AD cases and 4,965 controls, and (3) WES of an Enriched sample set comprised of 853 AD cases from multiply affected families and 171 Hispanic controls. The Case-Control and Enriched Case Study spans 24 cohorts provided by the Alzheimer’s Disease Genetics Consortium (ADGC) and the Cohorts for Heart and Aging Research in Genomic Epidemiology (CHARGE) Consortium.

Sequencing for these samples was conducted through three National Human Genome Research Institute (NHGRI) funded Large Scale Sequencing and Analysis Centers (LSACs): Baylor College of Medicine Human Genome Sequencing Center, the Broad Institute, the McDonnell Genome Institute at Washington University. The samples were sequenced on the Illumina HiSeq 2000/2500 platforms with 100bp paired-end reads. In the ADSP Discovery Case Control, 4586 samples were sequenced using the Illumina Rapid Capture Exome (ICE) kit and 6343 samples were sequenced using Roche Nimblegen’s VCRome v2.1 target capture kit. BAM files from hg37 build were sent to GCAD for processing on the VCPA1.1 pipeline. 10634 passed sequencing metrics and quality control.

snd10001- Alzheimer’s Disease Sequencing Project (ADSP) Extension

To further assess the genomes in multiply affected families, under funding provided by NHGRI, an additional 427 samples were whole genome sequenced. This included 107 additional samples from families studied under the Discovery Phase, 175 samples from 47 new families, and 145 Hispanic Controls. This portion of the study is called the Discovery Extension Phase. The Family Based Study spans seven cohorts provided by the Alzheimer’s Disease Genetics

Consortium (ADGC) and the Cohorts for Heart and Aging Research in Genomic Epidemiology (CHARGE) Consortium.

Under funding provided by NHGRI, an additional 3,000 subjects were whole genome sequenced. This included 1,466 cases and 1,534 controls. Of these 1,000 each of Non-Hispanic White (NHW), Caribbean Hispanic (CH), and African American (AA) descent were sequenced. Of these a total of 739 autopsy samples were sequenced [568 cases (500 NHW cases and 68 AA cases) and 171 controls (164 NHW and 7 AA)]. This Case-Control spans 5 cohorts provided by the Alzheimer's Disease Genetics Consortium (ADGC).

Sequencing for these samples was conducted through three National Human Genome Research Institute (NHGRI) funded Large Scale Sequencing and Analysis Centers (LSACs): Baylor College of Medicine Human Genome Sequencing Center, the Broad Institute, the McDonnell Genome Institute at Washington University. The samples were sequenced on the Illumina HiSeq X Ten platform with 150bp paired-end reads.

snd10002- Alzheimer's Disease Neuroimaging Initiative (ADNI)

ADNI is a global research study that actively supports the investigation and development of treatments that slow or stop the progression of AD. In this multisite longitudinal study, researchers at 63 sites in the US and Canada track the progression of AD in the human brain with clinical, imaging, genetic and biospecimen biomarkers through the process of normal aging, early mild cognitive impairment (EMCI), and late mild cognitive impairment (LMCI) to dementia or AD. Participants undergo a series of initial tests that are repeated at intervals over subsequent years, including a clinical evaluation, neuropsychological tests, genetic testing, lumbar puncture, and MRI and PET scans. The overall goal of ADNI is to validate biomarkers for use in Alzheimer' s disease clinical treatment trials. 1338 cases and 483 controls are included in this study across 4 phases.

Data used in the preparation of this article were obtained from the Alzheimer's Disease Neuroimaging Initiative (ADNI) database (adni.loni.usc.edu). The ADNI was launched in 2003 as a public-private partnership, led by Principal Investigator Michael W. Weiner, MD. The primary goal of ADNI has been to test whether serial magnetic resonance imaging

(MRI), positron emission tomography (PET), other biological markers, and clinical and neuropsychological assessment can be combined to measure the progression of mild cognitive impairment (MCI) and early Alzheimer’s disease (AD). For up-to-date information, see www.adni-info.org.

snd10011-Accelerating Medicines Partnership – Alzheimer’s Disease (AMP-AD)

AMP-AD samples from the ROSMAP, MayoRNAseq, and Mount Sinai Brain Bank cohorts were whole-genome sequenced at New York Genome Center on the HiSeqX machine. FASTQ files were sent to GCAD for processing on the VCPA1.1 pipeline. 1326 samples passed sequencing metrics and quality control.

snd10020-ADSP-Follow-up Study 1(FUS1)

The ADSP-FUS is a National Institute on Aging (NIA) initiative focused on identifying genetic risk and protective variants for late-onset Alzheimer Disease (LOAD). A concern in AD genetic studies is a lack of racial-ethnic diversity. The ADSP-FUS collects and sequences existing ethnically diverse and unique cohorts with clinical data to expand the utility of new discoveries for individuals from all populations.

ADSP FUS1 samples were whole-genome sequenced at USUHS either on the HiSeqX or NovaSeq machine. FASTQ files were sent to GCAD for processing on the VCPA1.1 pipeline. A total of 8,160 samples passed sequencing metrics and quality control.

This first release contains 3,250 AD cases, 4,149 cognitively normal individuals, 194 individuals with mild cognitive impairment, and 567 with unknown or other dementia from seven datasets (PR1066, ADC Autopsy, ADGC African American (release 1), HIHG Brain Bank, ADNI-WGS-2, APOE Extremes, and StEP AD).

snd10030-Alzheimer’s Disease Genetics Consortium Texas Alzheimer’s Research and Care Consortium (ADGC-TARCC)

The TARCC samples were sequenced at USUHS on the Novaseq machine. 1,018 samples were sequenced and FASTQ files were sent to GCAD for processing on the VCPA 1.1 pipeline. A total of 1,017 samples passed sequencing metrics and quality control checks.

snd10031-ADSP-Follow-up Study 2(FUS2)

The ADSP-FUS is a National Institute on Aging (NIA) initiative focused on identifying genetic risk and protective variants for late-onset Alzheimer Disease (LOAD). A concern in AD genetic studies is a lack of racial-ethnic diversity. The ADSP-FUS collects and sequences existing ethnically diverse and unique cohorts with clinical data to expand the utility of new discoveries for individuals from all populations.

ADSP FUS2 samples were whole-genome sequenced at either at the University of Miami on the HiSeqX or USUHS on the NovaSeq machine. 13228 samples were sequenced and FASTQ files were sent to GCAD for processing on the VCPA1.1 pipeline. A total of 12,621 samples passed sequencing metrics and quality control checks.

snd10032-Early-onset Alzheimer's Disease (EOAD1)

The EOAD samples were sequenced at USUHS on the NovaSeq machine. 3176 samples were sequenced and FASTQ files were sent to GCAD for processing on the VCPA 1.1 pipeline. A total of 3,132 samples passed sequencing metrics and quality control checks.

snd10033- The Diagnostic Assessment of Dementia for the Longitudinal Aging Study of India (LASI-DAD)

2,768 LASI-DAD respondents from 18 diverse ethno-linguistic and genographic groups across India who consented to the blood sample collection samples were sequenced at Medgenome on the HiSeqX machine. FASTQ files were sent to GCAD for processing on the VCPA 1.1 pipeline. A total of 2,686 samples passed sequencing metrics and quality control checks (including 6 technical replicates sequenced with ADSP rounds).

Clinical consensus diagnosis of dementia was achieved by using the Clinical Dementia Rating (CDR). At least three clinicians reviewed the case status and a second round of review by independent clinicians were conducted if the global ratings were different. For individuals without clinical consensus diagnoses, dementia status was predicted using a selected machine learning model, whose overall accuracy and agreement with the final consensus diagnoses

between the selected machine learning model and clinicians who participated in the clinical consensus diagnostic process were highest among all trained machine learning models.

Quality Control Filtering

The Genomic Center for Alzheimer Disease (GCAD) performs single nucleotide and insertion deletion variant calling for the ADSP. They deliver project level VCF files that include all variants called with flags indicating quality control (QC) metrics that can be used for filtering. For the current study the GCAD provided QC flags were used to identify and retain high quality variants within the ADSP VCFs. Specifically, for WGS data variants that did not receive a GATK pass, that were monomorphic across all samples, or had low call rate across all studies were excluded. Additionally, genotypes were set to missing for all individuals within a study if the variant had high mean depth or an ABhet ratio outside of 0.25 to 0.75. For WES data, the same filtering was implemented with the following modifications: genotypes were set to missing within a study if a low call rate was observed within that study and if variants were outside of the target region defined as the intersection of the target capture kits used by all contributing studies. Details of the GCAD provided QC flags can be found in the readme files at NIAGADS.

Race/Ethnicity Information

Reported race and ethnicity information in the phenotypic files from NIAGADS was combined to represent Hispanic and Non-Hispanic White, Hispanic and Non-Hispanic Black, Hispanic and Non-Hispanic Other/Unknown groups in the ADSP individuals. The other/unknown group includes Native Americans/Alaska Native, Asian, Native Hawaiian/Pacific Islander, other reported races, and unknown race.

Acknowledgement

Data for this study were prepared, archived, and distributed by the National Institute on Aging Alzheimer’s Disease Data Storage Site (NIAGADS) at the University of Pennsylvania (U24-AG041689), funded by the National Institute on Aging.

Alzheimer's Disease Sequencing Project (sa000001) data:

The Alzheimer’s Disease Sequencing Project (ADSP) is comprised of two Alzheimer’s Disease (AD) genetics consortia and three National Human Genome Research Institute (NHGRI) funded Large Scale Sequencing and Analysis Centers (LSAC). The two AD genetics consortia are the Alzheimer’s Disease Genetics Consortium (ADGC) funded by NIA (U01 AG032984), and the Cohorts for Heart and Aging Research in Genomic Epidemiology (CHARGE) funded by NIA (R01 AG033193), the National Heart, Lung, and Blood Institute (NHLBI), other National Institute of Health (NIH) institutes and other foreign governmental and non-governmental organizations. The Discovery Phase analysis of sequence data is supported through UF1AG047133 (to Drs. Schellenberg, Farrer, Pericak-Vance, Mayeux, and Haines); U01AG049505 to Dr. Seshadri; U01AG049506 to Dr. Boerwinkle; U01AG049507 to Dr. Wijsman; and U01AG049508 to Dr. Goate and the Discovery Extension Phase analysis is supported through U01AG052411 to Dr. Goate, U01AG052410 to Dr. Pericak-Vance and U01 AG052409 to Drs. Seshadri and Fornage.

Sequencing for the Follow Up Study (FUS) is supported through U01AG057659 (to Drs. PericakVance, Mayeux, and Vardarajan) and U01AG062943 (to Drs. Pericak-Vance and Mayeux). Data generation and harmonization in the Follow-up Phase is supported by U54AG052427 (to Drs. Schellenberg and Wang). The FUS Phase analysis of sequence data is supported through U01AG058589 (to Drs. Destefano, Boerwinkle, De Jager, Fornage, Seshadri, and Wijsman), U01AG058654 (to Drs. Haines, Bush, Farrer, Martin, and Pericak-Vance), U01AG058635 (to Dr. Goate), RF1AG058066 (to Drs. Haines, Pericak-Vance, and Scott), RF1AG057519 (to Drs. Farrer and Jun), R01AG048927 (to Dr. Farrer), and RF1AG054074 (to Drs. Pericak-Vance and Beecham).

The ADGC cohorts include: Adult Changes in Thought (ACT) (U01 AG006781, U19 AG066567), the Alzheimer’s Disease Research Centers (ADRC) (P30 AG062429, P30

AG066468, P30 AG062421, P30 AG066509, P30 AG066514, P30 AG066530, P30 AG066507, P30 AG066444, P30 AG066518, P30 AG066512, P30 AG066462, P30 AG072979, P30 AG072972, P30 AG072976, P30 AG072975, P30 AG072978, P30 AG072977, P30 AG066519, P30 AG062677, P30 AG079280, P30 AG062422, P30 AG066511, P30 AG072946, P30 AG062715, P30 AG072973, P30 AG066506, P30 AG066508, P30 AG066515, P30 AG072947, P30 AG072931, P30 AG066546, P20 AG068024, P20 AG068053, P20 AG068077, P20 AG068082, P30 AG072958, P30 AG072959), the Chicago Health and Aging Project (CHAP) (R01 AG11101, RC4 AG039085, K23 AG030944), Indiana Memory and Aging Study (IMAS) (R01 AG019771), Indianapolis Ibadan (R01 AG009956, P30 AG010133), the Memory and Aging Project (MAP) (R01 AG17917), Mayo Clinic (MAYO) (R01 AG032990, U01 AG046139, R01 NS080820, RF1 AG051504, P50 AG016574), Mayo Parkinson's Disease controls (NS039764, NS071674, 5RC2HG005605), University of Miami (R01 AG027944, R01 AG028786, R01 AG019085, IIRG09133827, A2011048), the Multi-Institutional Research in Alzheimer's Genetic Epidemiology Study (MIRAGE) (R01 AG09029, R01 AG025259), the National Centralized Repository for Alzheimer's Disease and Related Dementias (NCRAD) (U24 AG021886), the National Institute on Aging Late Onset Alzheimer's Disease Family Study (NIA- LOAD) (U24 AG056270), the Religious Orders Study (ROS) (P30 AG10161, R01 AG15819), the Texas Alzheimer's Research and Care Consortium (TARCC) (funded by the Darrell K Royal Texas Alzheimer's Initiative), Vanderbilt University/Case Western Reserve University (VAN/CWRU) (R01 AG019757, R01 AG021547, R01 AG027944, R01 AG028786, P01 NS026630, and Alzheimer's Association), the Washington Heights-Inwood Columbia Aging Project (WHICAP) (RF1 AG054023), the University of Washington Families (VA Research Merit Grant, NIA: P50AG005136, R01AG041797, NINDS: R01NS069719), the Columbia University Hispanic Estudio Familiar de Influencia Genetica de Alzheimer (EFIGA) (RF1 AG015473), the University of Toronto (UT) (funded by Wellcome Trust, Medical Research Council, Canadian Institutes of Health Research), and Genetic Differences (GD) (R01 AG007584). The CHARGE cohorts are supported in part by National Heart, Lung, and Blood Institute (NHLBI) infrastructure grant HL105756 (Psaty), RC2HL102419 (Boerwinkle) and the neurology working group is supported by the National Institute on Aging (NIA) R01 grant AG033193.

The CHARGE cohorts participating in the ADSP include the following: Austrian Stroke Prevention Study (ASPS), ASPS-Family study, and the Prospective Dementia Registry-Austria (ASPS/PRODEM-Aus), the Atherosclerosis Risk in Communities (ARIC) Study, the Cardiovascular Health Study (CHS), the Erasmus Rucphen Family Study (ERF), the Framingham Heart Study (FHS), and the Rotterdam Study (RS). ASPS is funded by the Austrian Science Fond (FWF) grant number P20545-P05 and P13180 and the Medical University of Graz. The ASPS-Fam is funded by the Austrian Science Fund (FWF) project I904), the EU Joint Programme – Neurodegenerative Disease Research (JPND) in frame of the BRIDGET project (Austria, Ministry of Science) and the Medical University of Graz and the Steiermärkische Krankenanstalten Gesellschaft. PRODEM-Austria is supported by the Austrian Research Promotion agency (FFG) (Project No. 827462) and by the Austrian National Bank (Anniversary Fund, project 15435. ARIC research is carried out as a collaborative study supported by NHLBI contracts (HHSN268201100005C, HHSN268201100006C, HHSN268201100007C, HHSN268201100008C, HHSN268201100009C, HHSN268201100010C, HHSN268201100011C, and HHSN268201100012C). Neurocognitive data in ARIC is collected by U01 2U01HL096812, 2U01HL096814, 2U01HL096899, 2U01HL096902, 2U01HL096917 from the NIH (NHLBI, NINDS, NIA and NIDCD), and with previous brain MRI examinations funded by R01-HL70825 from the NHLBI. CHS research was supported by contracts HHSN268201200036C, HHSN268200800007C, N01HC55222, N01HC85079, N01HC85080, N01HC85081, N01HC85082, N01HC85083, N01HC85086, and grants U01HL080295 and U01HL130114 from the NHLBI with additional contribution from the National Institute of Neurological Disorders and Stroke (NINDS). Additional support was provided by R01AG023629, R01AG15928, and R01AG20098 from the NIA. FHS research is supported by NHLBI contracts N01-HC-25195 and HHSN268201500001I. This study was also supported by additional grants from the NIA (R01s AG054076, AG049607 and AG033040 and NINDS (R01 NS017950). The ERF study as a part of EUROSPAN (European Special Populations Research Network) was supported by European Commission FP6 STRP grant number 018947 (LSHG-CT-2006-01947) and also received funding from the European Community's Seventh Framework Programme (FP7/2007-2013)/grant agreement HEALTH-F4-2007-201413 by the European Commission under the programme "Quality of Life and Management of the Living Resources" of 5th Framework Programme (no. QLG2-CT-2002-

01254). High-throughput analysis of the ERF data was supported by a joint grant from the Netherlands Organization for Scientific Research and the Russian Foundation for Basic Research (NWO-RFBR 047.017.043). The Rotterdam Study is funded by Erasmus Medical Center and Erasmus University, Rotterdam, the Netherlands Organization for Health Research and Development (ZonMw), the Research Institute for Diseases in the Elderly (RIDE), the Ministry of Education, Culture and Science, the Ministry for Health, Welfare and Sports, the European Commission (DG XII), and the municipality of Rotterdam. Genetic data sets are also supported by the Netherlands Organization of Scientific Research NWO Investments (175.010.2005.011, 911-03-012), the Genetic Laboratory of the Department of Internal Medicine, Erasmus MC, the Research Institute for Diseases in the Elderly (014-93-015; RIDE2), and the Netherlands Genomics Initiative (NGI)/Netherlands Organization for Scientific Research (NWO) Netherlands Consortium for Healthy Aging (NCHA), project 050-060-810. All studies are grateful to their participants, faculty and staff. The content of these manuscripts is solely the responsibility of the authors and does not necessarily represent the official views of the National Institutes of Health or the U.S. Department of Health and Human Services.

The FUS cohorts include: the Alzheimer’s Disease Research Centers (ADRC) (P30 AG062429, P30 AG066468, P30 AG062421, P30 AG066509, P30 AG066514, P30 AG066530, P30 AG066507, P30 AG066444, P30 AG066518, P30 AG066512, P30 AG066462, P30 AG072979, P30 AG072972, P30 AG072976, P30 AG072975, P30 AG072978, P30 AG072977, P30 AG066519, P30 AG062677, P30 AG079280, P30 AG062422, P30 AG066511, P30 AG072946, P30 AG062715, P30 AG072973, P30 AG066506, P30 AG066508, P30 AG066515, P30 AG072947, P30 AG072931, P30 AG066546, P20 AG068024, P20 AG068053, P20 AG068077, P20 AG068082, P30 AG072958, P30 AG072959), Alzheimer’s Disease Neuroimaging Initiative (ADNI) (U19AG024904), Amish Protective Variant Study (RF1AG058066), Cache County Study (R01AG11380, R01AG031272, R01AG21136, RF1AG054052), Case Western Reserve University Brain Bank (CWRUBB) (P50AG008012), Case Western Reserve University Rapid Decline (CWRURD) (RF1AG058267, NU38CK000480), CubanAmerican Alzheimer’s Disease Initiative (CuAADI) (3U01AG052410), Estudio Familiar de Influencia Genetica en Alzheimer (EFIGA) (5R37AG015473, RF1AG015473, R56AG051876), Genetic and Environmental Risk Factors for Alzheimer Disease Among African Americans Study (GenerAAtions) (2R01AG09029, R01AG025259,

2R01AG048927), Gwangju Alzheimer and Related Dementias Study (GARD) (U01AG062602), Hillblom Aging Network (2014-A-004-NET, R01AG032289, R01AG048234), Hussman Institute for Human Genomics Brain Bank (HIHGBB) (R01AG027944, Alzheimer's Association "Identification of Rare Variants in Alzheimer Disease"), Ibadan Study of Aging (IBADAN) (5R01AG009956), Longevity Genes Project (LGP) and LonGenity (R01AG042188, R01AG044829, R01AG046949, R01AG057909, R01AG061155, P30AG038072), Mexican Health and Aging Study (MHAS) (R01AG018016), Multi-Institutional Research in Alzheimer's Genetic Epidemiology (MIRAGE) (2R01AG09029, R01AG025259, 2R01AG048927), Northern Manhattan Study (NOMAS) (R01NS29993), Peru Alzheimer's Disease Initiative (PeADI) (RF1AG054074), Puerto Rican 1066 (PR1066) (Wellcome Trust (GR066133/GR080002), European Research Council (340755)), Puerto Rican Alzheimer Disease Initiative (PRADI) (RF1AG054074), Reasons for Geographic and Racial Differences in Stroke (REGARDS) (U01NS041588), Research in African American Alzheimer Disease Initiative (REAAADI) (U01AG052410), the Religious Orders Study (ROS) (P30 AG10161, P30 AG72975, R01 AG15819, R01 AG42210), the RUSH Memory and Aging Project (MAP) (R01 AG017917, R01 AG42210Stanford Extreme Phenotypes in AD (R01AG060747), University of Miami Brain Endowment Bank (MBB), University of Miami/Case Western/North Carolina A&T African American (UM/CASE/NCAT) (U01AG052410, R01AG028786), Wisconsin Registry for Alzheimer's Prevention (WRAP) (R01AG027161 and R01AG054047), Mexico-Southern California Autosomal Dominant Alzheimer's Disease Consortium (R01AG069013), Center for Cognitive Neuroscience and Aging (R01AG047649), and the A4 Study (R01AG063689, U19AG010483 and U24AG057437).

The four LSACs are: the Human Genome Sequencing Center at the Baylor College of Medicine (U54 HG003273), the Broad Institute Genome Center (U54HG003067), The American Genome Center at the Uniformed Services University of the Health Sciences (U01AG057659), and the Washington University Genome Institute (U54HG003079). Genotyping and sequencing for the ADSP FUS is also conducted at John P. Hussman Institute for Human Genomics (HIHG) Center for Genome Technology (CGT).

Biological samples and associated phenotypic data used in primary data analyses were stored at Study Investigators institutions, and at the National Centralized Repository for Alzheimer's

Disease and Related Dementias (NCRAD, U24AG021886) at Indiana University funded by NIA. Associated Phenotypic Data used in primary and secondary data analyses were provided by Study Investigators, the NIA funded Alzheimer's Disease Centers (ADCs), and the National Alzheimer's Coordinating Center (NACC, U24AG072122) and the National Institute on Aging Genetics of Alzheimer's Disease Data Storage Site (NIAGADS, U24AG041689) at the University of Pennsylvania, funded by NIA. Harmonized phenotypes were provided by the ADSP Phenotype Harmonization Consortium (ADSP-PHC), funded by NIA (U24 AG074855, U01 AG068057 and R01 AG059716) and Ultrascale Machine Learning to Empower Discovery in Alzheimer's Disease Biobanks (AI4AD, U01 AG068057). This research was supported in part by the Intramural Research Program of the National Institutes of health, National Library of Medicine. Contributors to the Genetic Analysis Data included Study Investigators on projects that were individually funded by NIA, and other NIH institutes, and by private U.S. organizations, or foreign governmental or nongovernmental organizations.

The ADSP Phenotype Harmonization Consortium (ADSP-PHC) is funded by NIA (U24 AG074855, U01 AG068057 and R01 AG059716). The harmonized cohorts within the ADSP-PHC include: the Anti-Amyloid Treatment in Asymptomatic Alzheimer's study (A4 Study), a secondary prevention trial in preclinical Alzheimer's disease, aiming to slow cognitive decline associated with brain amyloid accumulation in clinically normal older individuals. The A4 Study is funded by a public-private-philanthropic partnership, including funding from the National Institutes of Health-National Institute on Aging, Eli Lilly and Company, Alzheimer's Association, Accelerating Medicines Partnership, GHR Foundation, an anonymous foundation and additional private donors, with in-kind support from Avid and Cogstate. The companion observational Longitudinal Evaluation of Amyloid Risk and Neurodegeneration (LEARN) Study is funded by the Alzheimer's Association and GHR Foundation. The A4 and LEARN Studies are led by Dr. Reisa Sperling at Brigham and Women's Hospital, Harvard Medical School and Dr. Paul Aisen at the Alzheimer's Therapeutic Research Institute (ATRI), University of Southern California. The A4 and LEARN Studies are coordinated by ATRI at the University of Southern California, and the data are made available through the Laboratory for Neuro Imaging at the University of Southern California. The participants screening for the A4 Study provided permission to share their de-identified data in order to advance the quest to find a successful treatment for Alzheimer's disease. We would like to acknowledge the dedication of all the

participants, the site personnel, and all of the partnership team members who continue to make the A4 and LEARN Studies possible. The complete A4 Study Team list is available on: a4study.org/a4-study-team.; the Adult Changes in Thought study (ACT), U01 AG006781, U19 AG066567; Alzheimer's Disease Neuroimaging Initiative (ADNI): Data collection and sharing for this project was funded by the Alzheimer's Disease Neuroimaging Initiative (ADNI) (National Institutes of Health Grant U01 AG024904) and DOD ADNI (Department of Defense award number W81XWH-12-2-0012). ADNI is funded by the National Institute on Aging, the National Institute of Biomedical Imaging and Bioengineering, and through generous contributions from the following: AbbVie, Alzheimer's Association; Alzheimer's Drug Discovery Foundation; Araclon Biotech; BioClinica, Inc.; Biogen; Bristol-Myers Squibb Company; CereSpir, Inc.; Cogstate; Eisai Inc.; Elan Pharmaceuticals, Inc.; Eli Lilly and Company; EuroImmun; F. Hoffmann-La Roche Ltd and its affiliated company Genentech, Inc.; Fujirebio; GE Healthcare; IXICO Ltd.;Janssen Alzheimer Immunotherapy Research & Development, LLC.; Johnson & Johnson Pharmaceutical Research & Development LLC.; Lumosity; Lundbeck; Merck & Co., Inc.;Meso Scale Diagnostics, LLC.; NeuroRx Research; Neurotrack Technologies; Novartis Pharmaceuticals Corporation; Pfizer Inc.; Piramal Imaging; Servier; Takeda Pharmaceutical Company; and Transition Therapeutics. The Canadian Institutes of Health Research is providing funds to support ADNI clinical sites in Canada. Private sector contributions are facilitated by the Foundation for the National Institutes of Health (www.fnih.org). The grantee organization is the Northern California Institute for Research and Education, and the study is coordinated by the Alzheimer's Therapeutic Research Institute at the University of Southern California. ADNI data are disseminated by the Laboratory for Neuro Imaging at the University of Southern California; Estudio Familiar de Influencia Genetica en Alzheimer (EFIGA): 5R37AG015473, RF1AG015473, R56AG051876; the Health & Aging Brain Study – Health Disparities (HABS-HD), supported by the National Institute on Aging of the National Institutes of Health under Award Numbers R01AG054073, R01AG058533, R01AG070862, P41EB015922, and U19AG078109; the Korean Brain Aging Study for the Early Diagnosis and Prediction of Alzheimer's disease (KBASE), which was supported by a grant from Ministry of Science, ICT and Future Planning (Grant No: NRF-2014M3C7A1046042); Memory & Aging Project at Knight Alzheimer's Disease Research Center (MAP at Knight ADRC): The Memory and Aging Project at the Knight-ADRC (Knight-

ADRC). This work was supported by the National Institutes of Health (NIH) grants R01AG064614, R01AG044546, RF1AG053303, RF1AG058501, U01AG058922 and R01AG064877 to Carlos Cruchaga. The recruitment and clinical characterization of research participants at Washington University was supported by NIH grants P30AG066444, P01AG03991, and P01AG026276. Data collection and sharing for this project was supported by NIH grants RF1AG054080, P30AG066462, R01AG064614 and U01AG052410. We thank the contributors who collected samples used in this study, as well as patients and their families, whose help and participation made this work possible. This work was supported by access to equipment made possible by the Hope Center for Neurological Disorders, the Neurogenomics and Informatics Center (NGI: https://neurogenomics.wustl.edu/) and the Departments of Neurology and Psychiatry at Washington University School of Medicine; National Alzheimer's Coordinating Center (NACC): The NACC database is funded by NIA/NIH Grant U24 AG072122. SCAN is a multi-institutional project that was funded as a U24 grant (AG067418) by the National Institute on Aging in May 2020. Data collected by SCAN and shared by NACC are contributed by the NIA-funded ADRCs as follows: P30 AG062429 (PI James Brewer, MD, PhD), P30 AG066468 (PI Oscar Lopez, MD), P30 AG062421 (PI Bradley Hyman, MD, PhD), P30 AG066509 (PI Thomas Grabowski, MD), P30 AG066514 (PI Mary Sano, PhD), P30 AG066530 (PI Helena Chui, MD), P30 AG066507 (PI Marilyn Albert, PhD), P30 AG066444 (PI John Morris, MD), P30 AG066518 (PI Jeffrey Kaye, MD), P30 AG066512 (PI Thomas Wisniewski, MD), P30 AG066462 (PI Scott Small, MD), P30 AG072979 (PI David Wolk, MD), P30 AG072972 (PI Charles DeCarli, MD), P30 AG072976 (PI Andrew Saykin, PsyD), P30 AG072975 (PI David Bennett, MD), P30 AG072978 (PI Neil Kowall, MD), P30 AG072977 (PI Robert Vassar, PhD), P30 AG066519 (PI Frank LaFerla, PhD), P30 AG062677 (PI Ronald Petersen, MD, PhD), P30 AG079280 (PI Eric Reiman, MD), P30 AG062422 (PI Gil Rabinovici, MD), P30 AG066511 (PI Allan Levey, MD, PhD), P30 AG072946 (PI Linda Van Eldik, PhD), P30 AG062715 (PI Sanjay Asthana, MD, FRCP), P30 AG072973 (PI Russell Swerdlow, MD), P30 AG066506 (PI Todd Golde, MD, PhD), P30 AG066508 (PI Stephen Strittmatter, MD, PhD), P30 AG066515 (PI Victor Henderson, MD, MS), P30 AG072947 (PI Suzanne Craft, PhD), P30 AG072931 (PI Henry Paulson, MD, PhD), P30 AG066546 (PI Sudha Seshadri, MD), P20 AG068024 (PI Erik Roberson, MD, PhD), P20 AG068053 (PI Justin Miller, PhD), P20 AG068077 (PI Gary Rosenberg, MD), P20 AG068082 (PI Angela Jefferson, PhD),

P30 AG072958 (PI Heather Whitson, MD), P30 AG072959 (PI James Leverenz, MD); National Institute on Aging Alzheimer's Disease Family Based Study (NIA-AD FBS): U24 AG056270; Religious Orders Study (ROS): P30AG10161,R01AG15819, R01AG42210; Memory and Aging Project (MAP - Rush): R01AG017917, R01AG42210; Minority Aging Research Study (MARS): R01AG22018, R01AG42210; the Texas Alzheimer's Research and Care Consortium (TARCC), funded by the Darrell K Royal Texas Alzheimer's Initiative, directed by the Texas Council on Alzheimer's Disease and Related Disorders; Washington Heights/Inwood Columbia Aging Project (WHICAP): RF1 AG054023;and Wisconsin Registry for Alzheimer's Prevention (WRAP): R01AG027161 and R01AG054047. Additional acknowledgments include the National Institute on Aging Genetics of Alzheimer's Disease Data Storage Site (NIAGADS, U24AG041689) at the University of Pennsylvania, funded by NIA.

Alzheimer's Disease Neuroimaging Initiative (sa000002) data:

Data collection and sharing for this project was funded by the Alzheimer's Disease Neuroimaging Initiative (ADNI) (National Institutes of Health Grant U01 AG024904) and DOD ADNI (Department of Defense award number W81XWH-12-2-0012). ADNI is funded by the National Institute on Aging, the National Institute of Biomedical Imaging and Bioengineering, and through generous contributions from the following: AbbVie, Alzheimer's Association; Alzheimer's Drug Discovery Foundation; Araclon Biotech; BioClinica, Inc.; Biogen; Bristol-Myers Squibb Company; CereSpir, Inc.; Cogstate; Eisai Inc.; Elan Pharmaceuticals, Inc.; Eli Lilly and Company; EuroImmun; F. Hoffmann-La Roche Ltd and its affiliated company Genentech, Inc.; Fujirebio; GE Healthcare; IXICO Ltd.; Janssen Alzheimer Immunotherapy Research & Development, LLC.; Johnson & Johnson Pharmaceutical Research & Development LLC.; Lumosity; Lundbeck; Merck & Co., Inc.; Meso Scale Diagnostics, LLC.; NeuroRx Research; Neurotrack Technologies; Novartis Pharmaceuticals Corporation; Pfizer Inc.; Piramal Imaging; Servier; Takeda Pharmaceutical Company; and Transition Therapeutics. The Canadian Institutes of Health Research is providing funds to support ADNI clinical sites in Canada. Private sector contributions are facilitated by the Foundation for the National Institutes of Health (www.fnih.org). The grantee organization is the Northern California Institute for Research and Education, and the study is coordinated by the Alzheimer's Therapeutic

Research Institute at the University of Southern California. ADNI data are disseminated by the Laboratory for Neuro Imaging at the University of Southern California.

Additional information to include in an acknowledgment statement can be found on the LONI site: https://adni.loni.usc.edu/wp-content/uploads/how_to_apply/ADNI_Data_Use_Agreement.pdf.

Alzheimer's Disease Genetics Consortium (sa000003) data:

The Alzheimer's Disease Genetics Consortium (ADGC) supported sample preparation, sequencing and data processing through NIA grant U01AG032984. Sequencing data generation and harmonization is supported by the Genome Center for Alzheimer's Disease, U54AG052427, and data sharing is supported by NIAGADS, U24AG041689. Samples from the National Centralized Repository for Alzheimer's Disease and Related Dementias (NCRAD), which receives government support under a cooperative agreement grant (U24 AG021886) awarded by the National Institute on Aging (NIA), were used in this study. We thank contributors who collected samples used in this study, as well as patients and their families, whose help and participation made this work possible.

ADGC-TARCC (snd10030) data:

This study was made possible by the Texas Alzheimer's Research and Care Consortium (TARCC) funded by the state of Texas through the Texas Council on Alzheimer's Disease and Related Disorders and the Darrell K Royal Texas Alzheimer's Initiative.

Accelerating Medicines Partnership-Alzheimer's Disease (AMP-AD) (sa000011) data:

Mayo RNAseq Study- Study data were provided by the following sources: The Mayo Clinic Alzheimer's Disease Genetic Studies, led by Dr. Nilufer Ertekin-Taner and Dr. Steven G. Younkin, Mayo Clinic, Jacksonville, FL using samples from the Mayo Clinic Study of Aging, the Mayo Clinic Alzheimer's Disease Research Center, and the Mayo Clinic Brain Bank. Data collection was supported through funding by NIA grants P50 AG016574, R01 AG032990, U01 AG046139, R01 AG018023, U01 AG006576, U01 AG006786, R01 AG025711, R01 AG017216, R01 AG003949, NINDS grant R01 NS080820, CurePSP Foundation, and support from Mayo Foundation. Study data includes samples collected through the Sun Health Research Institute

Brain and Body Donation Program of Sun City, Arizona. The Brain and Body Donation Program is supported by the National Institute of Neurological Disorders and Stroke (U24 NS072026 National Brain and Tissue Resource for Parkinson's Disease and Related Disorders), the National Institute on Aging (P30 AG19610 Arizona Alzheimer's Disease Core Center), the Arizona Department of Health Services (contract 211002, Arizona Alzheimer's Research Center), the Arizona Biomedical Research Commission (contracts 4001, 0011, 05-901 and 1001 to the Arizona Parkinson's Disease Consortium) and the Michael J. Fox Foundation for Parkinson's Research

ROSMAP- We are grateful to the participants in the Religious Order Study, the Memory and Aging Project. This work is supported by the US National Institutes of Health [U01 AG046152, R01 AG043617, R01 AG042210, R01 AG036042, R01 AG036836, R01 AG032990, R01 AG18023, RC2 AG036547, P50 AG016574, U01 ES017155, KL2 RR024151, K25 AG041906-01, R01 AG30146, P30 AG10161, R01 AG17917, R01 AG15819, K08 AG034290, P30 AG10161 and R01 AG11101.

Mount Sinai Brain Bank (MSBB)- This work was supported by the grants R01AG046170, RF1AG054014, RF1AG057440 and R01AG057907 from the NIH/National Institute on Aging (NIA). R01AG046170 is a component of the AMP-AD Target Discovery and Preclinical Validation Project. Brain tissue collection and characterization was supported by NIH HHSN271201300031C.

The Diagnostic Assessment of Dementia for the Longitudinal Aging Study of India (LASI-DAD) (sa000019) data:

The Longitudinal Aging Study in India, Diagnostic Assessment of Dementia data is sponsored by the National Institute on Aging (grant numbers R01AG051125 and U01AG065958) and is conducted by the University of Southern California.

Dissecting the Genomic Etiology of non-Mendelian Early-Onset Alzheimer Disease (EOAD) and Related Phenotypes (sa000023) data:

This work was supported by the National Institutes of Health (NIH) grant R01AG064614. The ADSP-FUS is supported by U01AG057659.

The National Institutes of Health, National Institute on Aging (NIH-NIA) supported this work through the following grants: ADGC, U01 AG032984, RC2 AG036528; samples from the National Centralized Repository for Alzheimer's Disease and Related Dementias (NCRAD), which receives government support under a cooperative agreement grant (U24 AG21886) awarded by the National Institute on Aging (NIA), were used in this study. Sequencing data generation and harmonization is supported by the Genome Center for Alzheimer's Disease, U54AG052427, and data sharing is supported by NIAGADS, U24AG041689. We thank contributors who collected samples used in this study, as well as patients and their families, whose help and participation made this work possible.

NIH grants supported enrollment and data collection for the individual studies including the Alzheimer's Disease Centers (ADC, P30 AG062429 (PI James Brewer, MD, PhD), P30 AG066468 (PI Oscar Lopez, MD), P30 AG062421 (PI Bradley Hyman, MD, PhD), P30 AG066509 (PI Thomas Grabowski, MD), P30 AG066514 (PI Mary Sano, PhD), P30 AG066530 (PI Helena Chui, MD), P30 AG066507 (PI Marilyn Albert, PhD), P30 AG066444 (PI John Morris, MD), P30 AG066518 (PI Jeffrey Kaye, MD), P30 AG066512 (PI Thomas Wisniewski, MD), P30 AG066462 (PI Scott Small, MD), P30 AG072979 (PI David Wolk, MD), P30 AG072972 (PI Charles DeCarli, MD), P30 AG072976 (PI Andrew Saykin, PsyD), P30 AG072975 (PI David Bennett, MD), P30 AG072978 (PI Neil Kowall, MD), P30 AG072977 (PI Robert Vassar, PhD), P30 AG066519 (PI Frank LaFerla, PhD), P30 AG062677 (PI Ronald Petersen, MD, PhD), P30 AG079280 (PI Eric Reiman, MD), P30 AG062422 (PI Gil Rabinovici, MD), P30 AG066511 (PI Allan Levey, MD, PhD), P30 AG072946 (PI Linda Van Eldik, PhD), P30 AG062715 (PI Sanjay Asthana, MD, FRCP), P30 AG072973 (PI Russell Swerdlow, MD), P30 AG066506 (PI Todd Golde, MD, PhD), P30 AG066508 (PI Stephen Strittmatter, MD, PhD), P30 AG066515 (PI Victor Henderson, MD, MS), P30 AG072947 (PI Suzanne Craft, PhD), P30 AG072931 (PI Henry Paulson, MD, PhD), P30 AG066546 (PI Sudha Seshadri, MD), P20 AG068024 (PI Erik Roberson, MD, PhD), P20 AG068053 (PI Justin Miller, PhD), P20 AG068077 (PI Gary Rosenberg, MD), P20 AG068082 (PI Angela Jefferson, PhD), P30 AG072958 (PI Heather Whitson, MD), P30 AG072959 (PI James Leverenz, MD). The Miami ascertainment and research were supported in part through: RF1AG054080, R01AG027944, R01AG019085, R01AG028786-02, RC2AG036528. The Columbia ascertainment and research were supported in part through: R37AG015473 and U24AG056270. The University of

Washington ascertainment and research were supported in part through R01AG044546, RF1AG053303, RF1AG058501, U01AG058922 and R01AG064877.

This work was supported in part by NIA grants U01AG058589 and U01AG068221. ADSP data for this study were prepared, archived, and distributed by the National Institute on Aging Alzheimer's Disease Data Storage Site (NIAGADS) at the University of Pennsylvania (U24-AG041689), funded by the National Institute on Aging (accession NG00067). The full acknowledgement statement for the ADSP, which includes funding information, can be found at: https://dss.niagads.org/datasets/ng00067/ .

Table S1. Descriptive Statistics in the Cognitively Healthy Samples.

	**Cognitively Healthy (n=1,784)**	**General Control (n=11,508)**
Female	1,151 (65%)	6,929 (60%)
Baseline Age (years)	82.2 (5.3)	71.7 (8.6)
Age Cognitively normal (years)	87 (2.5)	75 (6.2)
Age of Onset (years)	N/A	76.6 (6.1)

*Genotyped *APOE* allele counts were reported.

Table S2. Gene-based Test Results for Noncoding Variants in the Analyses.

Gene	CHR	Category	#SNV	cMAC	STAAR-O
**AD sample**					
HLA-F	6	Upstream	51	625	8.84×10^{-7}
**Cognitive healthy sample**					
MTERF2	12	UTR	140	1261	8.13×10^{-7}
STAMBP	2	Upstream	43	658	1.70×10^{-7}
PLXNB2	22	Upstream	11	24	8.03×10^{-9}
SULT1B1	4	Enhancer DHS	504	4163	5.61×10^{-7}
CARD11	7	Enhancer DHS	906	9615	3.42×10^{-8}
LINC01880	2	ncRNA	90	881	6.56×10^{-8}

*Showing genes with significant or suggestive association with ADRD or Cognitive healthy status.

**Abbreviations: Chromosome (Chr), single nucleotide variant (SNV), cumulative minor allele count (cMAC), untranslated region (UTR), DNase I hypersensitive site (DHS), noncoding RNA (ncRNA).

Table S3. Single Variant Test Results for *SSAN1* in the Cognitively Healthy Sample.

Chr	Position	Ref	Alt	N	MAC	β	SE	p-value
9	137188778	T	C	13292	1	-1.12	3.28	7.32E-01
9	137189129	A	G	13292	14	1.21	0.91	1.83E-01
9	137189131	A	C	13292	1	68.95	8.36	1.68E-16

Chr: Chromosome; MAC: minor allele count; SE: Standard Error.

Table S4. Single Variant Test Results for *ZNF200* in the AD Sample.

Chr	Position	REF	ALT	N	MAC	β	SE	p-value
16	3223922	G	T	23450	1	1.77	2.03	3.85E-01
16	3223936	G	A	23450	15	-0.19	0.59	7.53E-01
16	3224175	T	C	23451	1	-1.18	2.83	6.77E-01
16	3224196	A	G	23450	1	-1.24	2.58	6.31E-01
16	3224259	T	G	23452	3	0.05	1.25	9.67E-01
16	3224612	T	C	23450	3	0.243	1.32	8.56E-01
16	3233837	C	T	23451	1	47.66	6.98	8.46E-12

Chr: Chromosome; MAC: minor allele count; SE: Standard Error.

Table S5. Gene-based Test Results for Noncoding Variants in the Analyses after Implementing Modification #2.

Gene	CHR	Category	#SNV	cMAC	STAAR-O
**AD sample**					
HLA-F	6	Upstream	51	625	$8.84\ x10^{-7}$
**Cognitive healthy sample**					
MTERF2	12	UTR	140	1261	8.13x10-7
STAMBP	2	Upstream	43	658	1.70x10-7
PLXNB2	22	Upstream	11	24	5.35x10-9
SULT1B1	4	Enhancer DHS	504	4163	5.61x10-7
CARD11	7	Enhancer DHS	906	9615	3.42x10-8
LINC01880	2	ncRNA	90	881	6.56x10-8

*Showing genes with significant or suggestive association with ADRD or Cognitive healthy status.

**Abbreviations: Chromosome (Chr), single nucleotide variant (SNV), cumulative minor allele count (cMAC), untranslated region (UTR), DNase I hypersensitive site (DHS), noncoding RNA (ncRNA).

Figure S1. QQ-plots of the Gene-based Tests in the ADRD and Cognitively Healthy using the Original STAAR Framework.

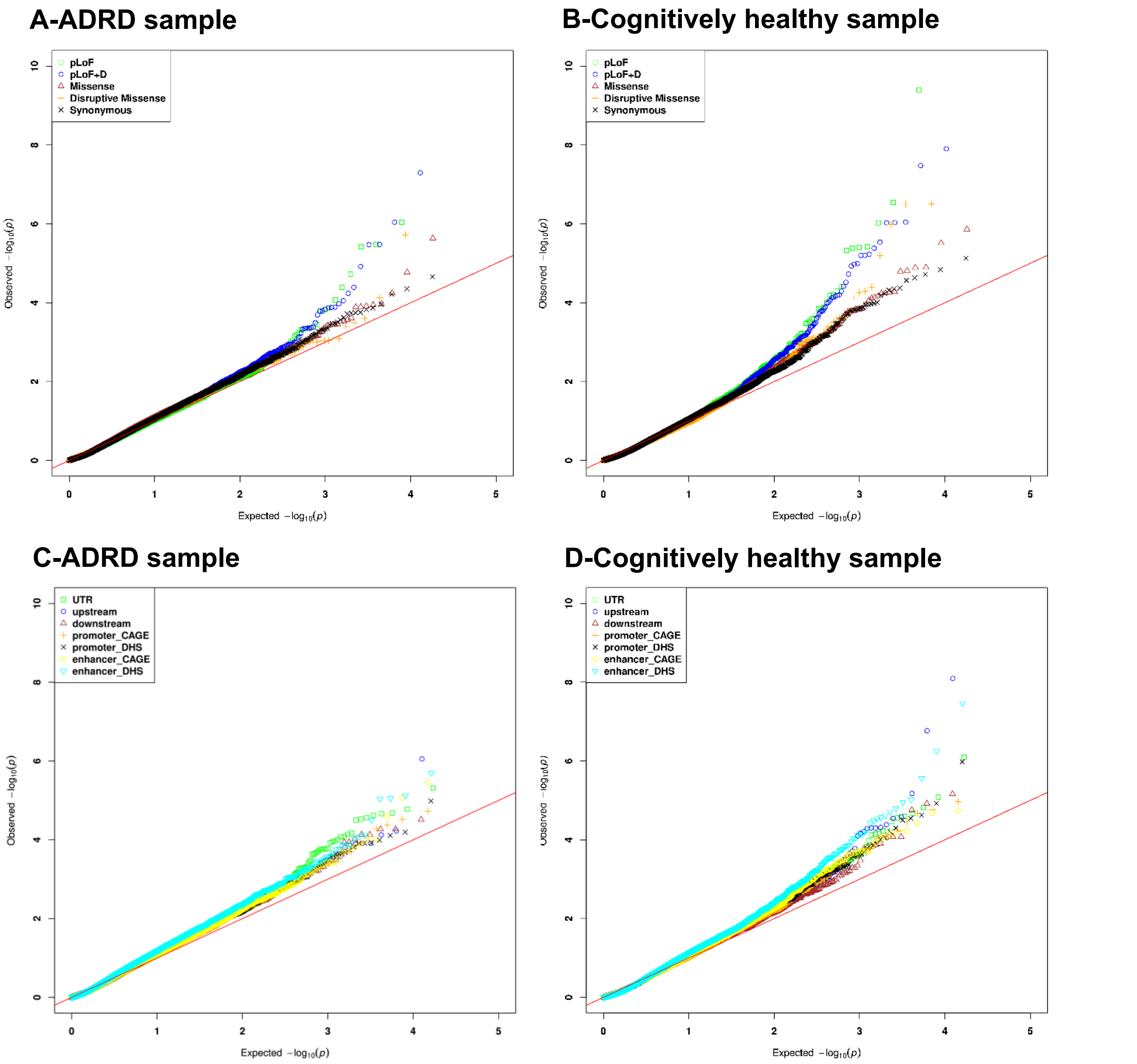

QQ-plots showing the distributions of gene-based test p-values from the original STAAR framework in the ADRD and cognitive healthy samples. A: gene-based test results for coding variants in the ADRD sample. B: gene-based test results for coding variant in the cognitive healthy sample. C: gene-based test results for noncoding variants in the ADRD sample. D: gene-based test results for noncoding variants in the cognitive healthy sample.

Figure S2. Manhattan Plots of the Gene-based Test Results in the ADRD and Cognitively Healthy Samples after implementing modification #1 to the STAAR framework.

A-ADRD sample

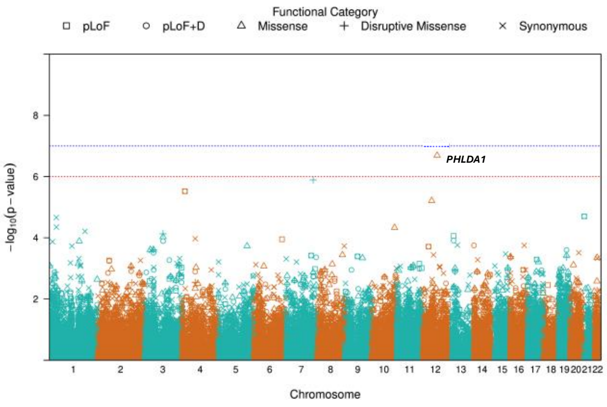

B-Cognitively healthy sample

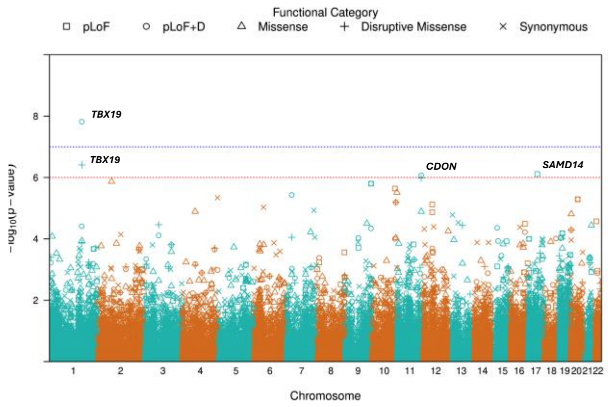

C-ADRD sample

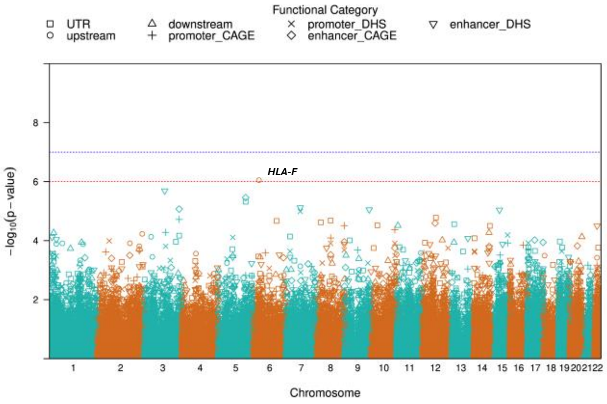

D-Cognitively healthy sample

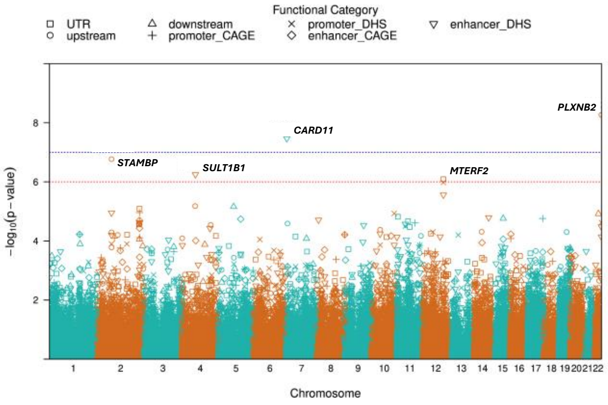

Manhattan plots showing gene-based test results for coding and noncoding variants after applying the modification #1 to the STAAR framework. The blue reference line represents the genome-wide significance threshold of 1x10^{-7} and the red reference line represents a suggestively significant threshold of 1x10-6. Specifically, panel A and C are gene-based test results for coding and noncoding variants in the ADRD sample, respectively. Panel B and D are gene-based test results for coding and noncoding variants in the cognitively healthy sample, respectively. Y-axis shows -log10(p-value) while x-axis represents the location of genes on chromosomes.

Figure S3. QQ-plots of the Gene-based Tests in the ADRD and Cognitively Healthy Samples after implementing modification #1 to the STAAR framework.

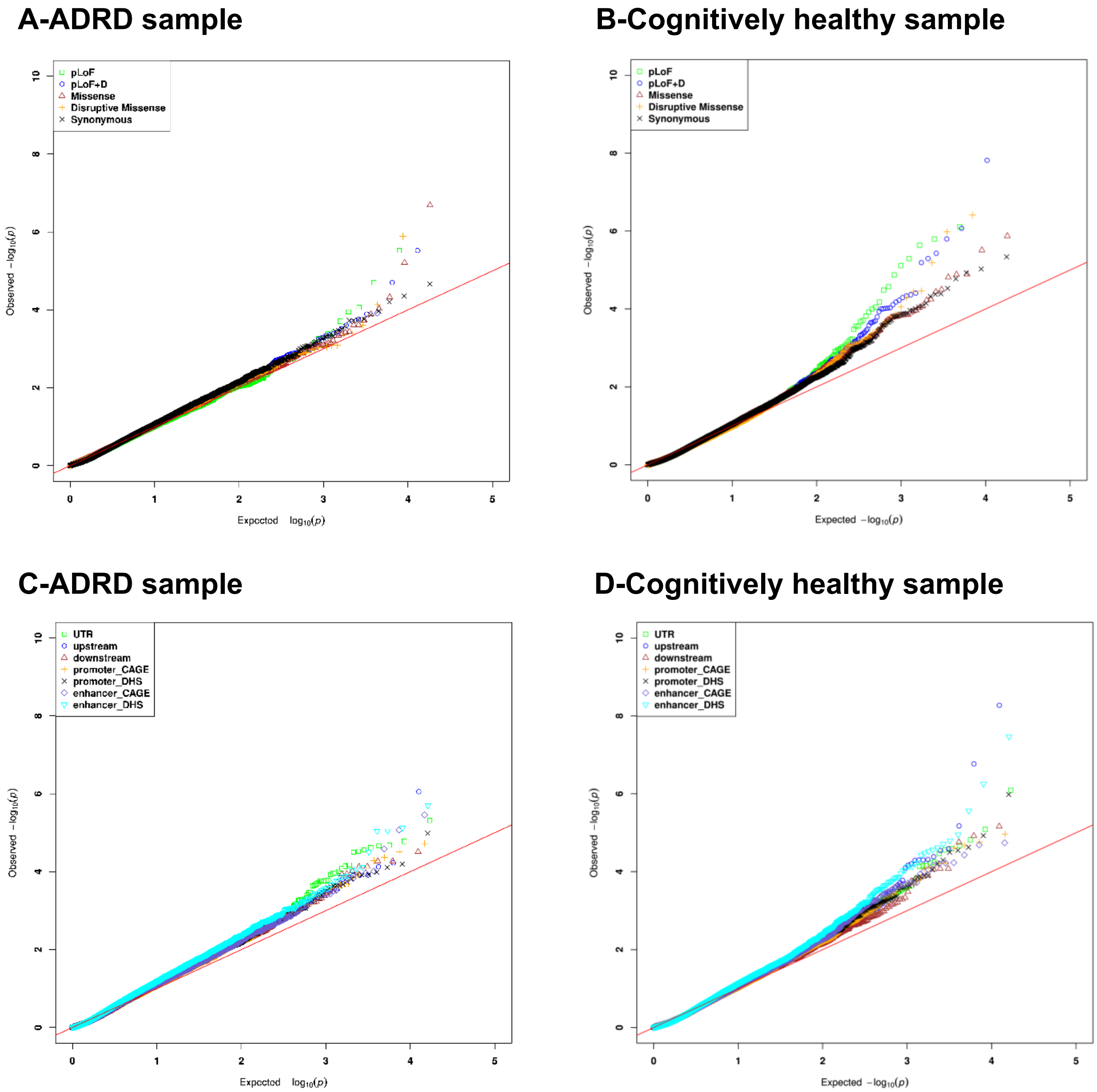

QQ-plots showing the distributions of gene-based test p-values after applying modification #1 to the STAAR framework in the ADRD and cognitive healthy samples. A: gene-based test results for coding variants in the ADRD sample. B: gene-based test results for coding variants in the cognitive healthy sample. C: gene-based test results for noncoding variants in the ADRD sample. D: gene-based test results for noncoding variants in the cognitive healthy sample.

Figure S4. Manhattan Plots of the Gene-based Tests of Noncoding Variants in the ADRD and Cognitively Healthy Samples before and after implementing modification #2.

A-ADRD sample

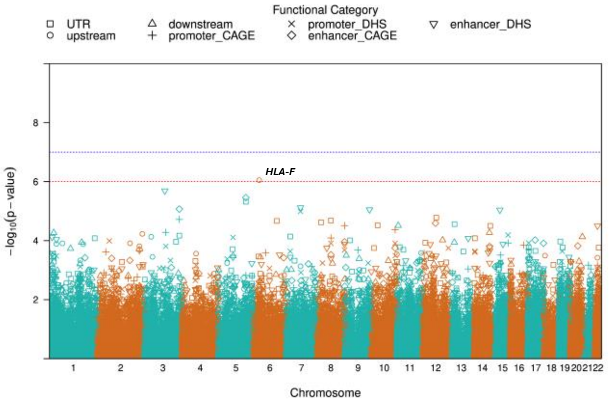

B- Cognitively healthy sample

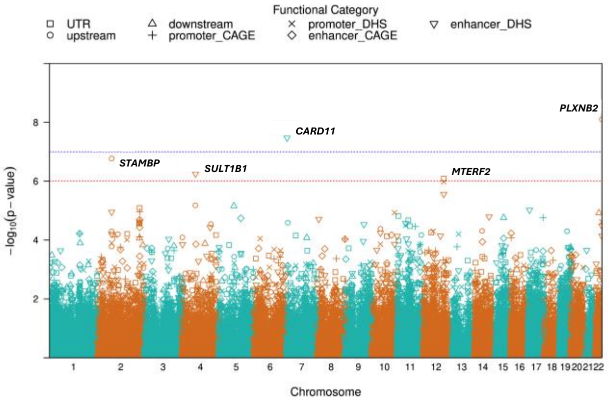

C-ADRD sample

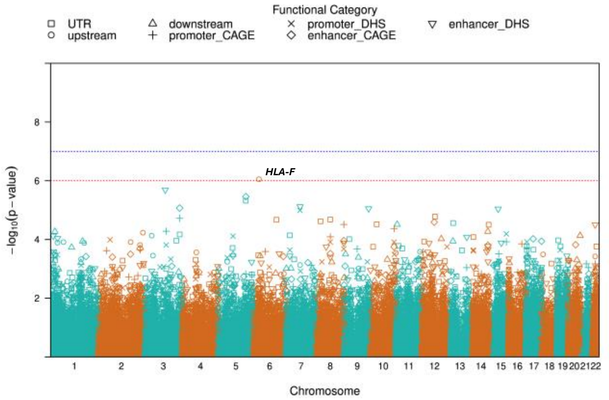

D- Cognitively healthy sample

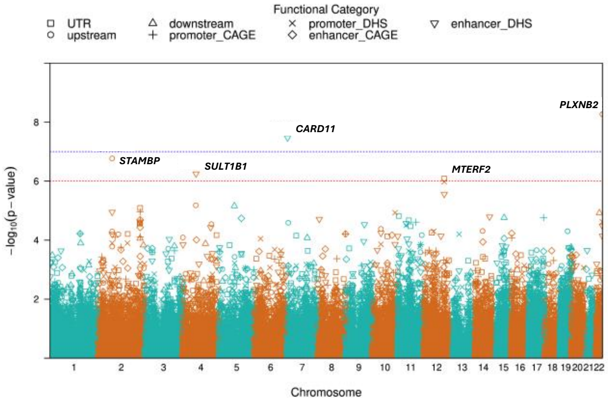

Manhattan plots showing gene-based test results for noncoding variants after applying modification #2 to the STAAR framework. The blue reference line represents the genome-wide significance threshold of $1x10^{-7}$ and the red reference line represents a suggestively significant threshold of $1x10^{-6}$. Specifically, panel A and C are gene-based test results before and after applying modifications #2 in the ADRD sample. Panel B and D are gene-based test results before and after applying modifications #2 in the cognitively healthy sample. Y-axis shows -log10(p-value) while x-axis represents the location of genes on chromosomes.

Figure S5. QQ-plots of the Gene-based Tests in the ADRD and Cognitively Healthy Samples after implementing modification #2 to the STAAR framework.

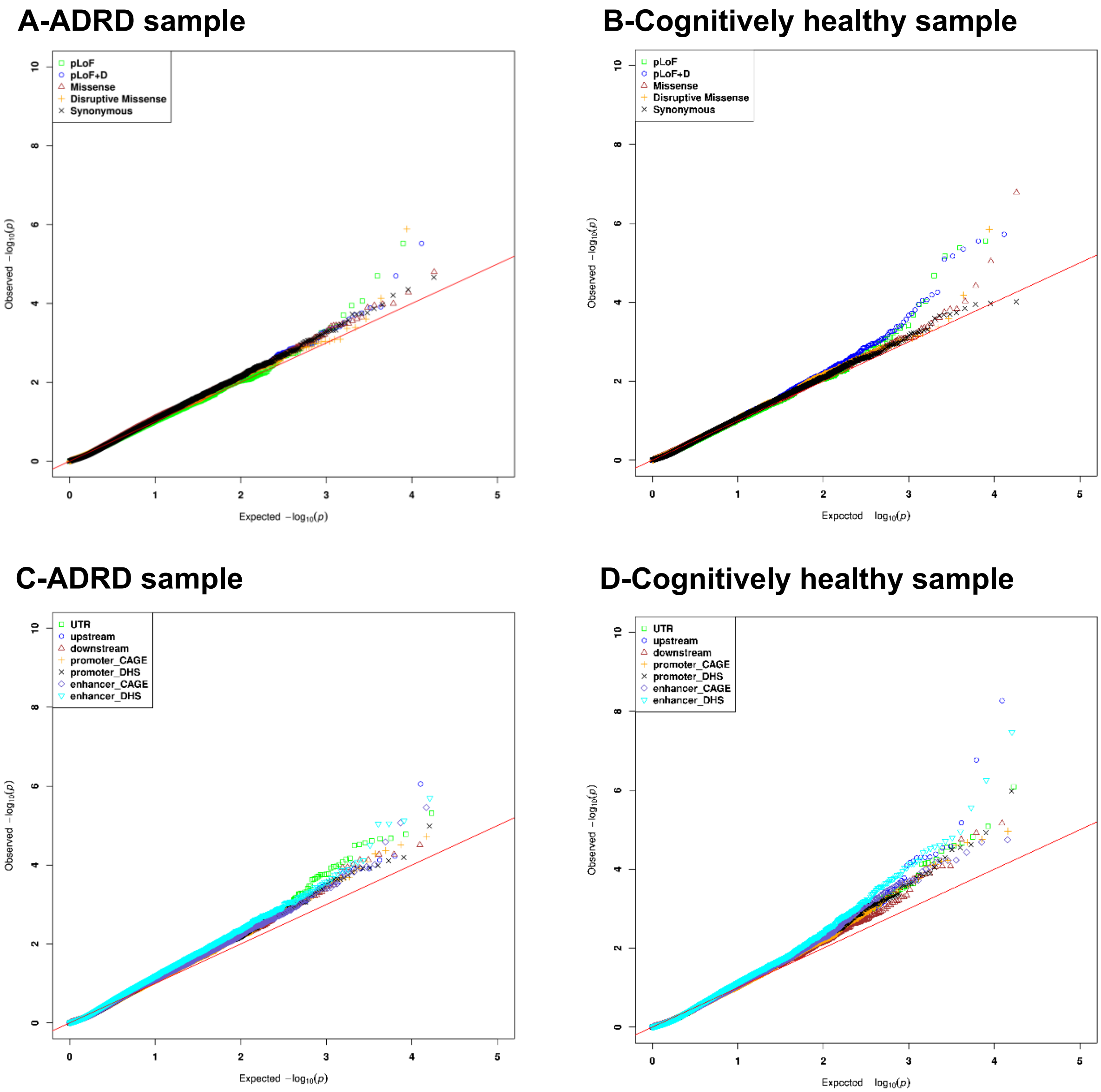

QQ-plots showing the distributions of gene-based test p-values after applying modification #2 to the STAAR framework in the ADRD and cognitive healthy samples. A: gene-based test results for coding variants in the ADRD sample. B: gene-based test results for coding variant in the cognitive healthy sample. C: gene-based test results for noncoding variants in the ADRD sample. D: gene-based test results for noncoding variants in the cognitive healthy sample.
