# Supplementary material for: Application of the STAAR framework in detecting rare variant associations with Alzheimer disease and related dementias: Insights and implications
Source: HGG Adv. 2026 Jan 20;7(2):100574. doi: 10.1016/j.xhgg.2026.100574 (PMC12934298; doi:10.1016/j.xhgg.2026.100574)
Supplement: Document S2. Article plus supplemental information [file mmc2.pdf]

HGG
Advances

ARTICLE

Application of the STAAR framework in detecting rare variant associations with Alzheimer disease and related dementias: Insights and implications

Dongyu Wang,[1] Sabrina Abbruzzese,[1] Nancy Heard-Costa,[2,3] Andy Rampersaud,[4] Eden Martin,[5,6] Adam Naj,[7,8] Bilcag Akgun,[5] Brian Kunkle,[5,6] Sudha Seshadri,[2,3,9] Gina Peloso,[1] The Alzheimer's Disease Neuroimaging Initiative,[14] The Alzheimer's Disease Sequencing Project,[14] Anita L. DeStefano,[1,2] Zilin Li,[10] Xihao Li,[11,12,13,*] and Seung Hoan Choi[1,13,15,*]

Summary

Rare genetic variation is considered a potential source of heritability in individuals with sporadic Alzheimer disease and related dementias (ADRD). The Variant-set test for association using annotation information (STAAR) framework leverages multiple functional annotations of genetic variants and combines association statistics from multiple variant aggregation-based methods, including burden, sequence kernel association test (SKAT), and aggregated Cauchy association test (ACAT-V), into a single measure of significance. Using whole-genome sequencing data from the Alzheimer's Disease Sequencing Project (ADSP), we comprehensively examined the association of rare genetic variation with ADRD in 23,454 individuals (37% individuals affected by ADRD) and with cognitively healthy elder status in 13,292 individuals (13% cognitively healthy elders) from diverse populations via the STAAR framework. We identified several genes significantly associated with ADRD or cognitively healthy status. However, our analysis revealed several limitations within the STAAR framework incorporating ultra-rare variants with dichotomous outcomes. To enhance the robustness of the framework, we proposed several computational refinements, including creating a burden of ultra-rare variants and employing more precise annotations to match the expected mechanism. After implementing the proposed modifications, the association with ADRD for *ZNF200* was no longer statistically significant ($\alpha = 1 \times 10^{-7}$), while *TBX19*, *PLXNB2*, *CARD11*, and *LINC01880* remained significantly associated with cognitively healthy status. We identified and addressed the computational limitations in the STAAR framework that could lead to potential spurious results for ultra-rare variant aggregates with an extremely low cumulative minor-allele count. Our proposed refinements produced more robust results for associations with rare variants in the context of dichotomous outcomes.

Introduction

Alzheimer disease (AD), which has a prevalence of 10.9% among people aged 65 and older in the United States,[1] remains the primary cause of dementia.[2] While Mendelian forms of AD account for only about 1% of affected individuals, the majority of instances are sporadic, highlighting the necessity of finding genetic variants that are associated with the disease.[3] Previous twin studies estimated the heritability of AD to be about 70%, but the estimated SNP heritability was only 3.1% in genome-wide association studies (GWASs).[4,5] The gap in heritability estimates between these studies can be attributed to many factors, of which rare genetic variation is a widely discussed potential source of the "missing heritability."[6] Many genetic studies focus on the broader phenotypic category of AD and related dementias (ADRD), which affect more than 55 million people worldwide.[7] Bellenguez et al. performed a GWAS in 788,989 individuals with European ancestry and reported rare single-variant associations with ADRD in *TREM2*, *PLCG2*, and *ABI3* genes; it noted limitations such as insufficient statistical power due to difficulties in the identification of rare variants with very low frequencies (e.g., <0.01%).[8] Recent advances in next-generation sequencing have enabled short- and long-read sequencing of the whole genome or exome for robust identification of rare variants.[9–11] These new technologies have led researchers to focus on rare genetic variants in

[1]Department of Biostatistics, Boston University School of Public Health, Boston, MA 02118, USA; [2]Department of Neurology, Boston University Chobanian & Avedisian School of Medicine, Boston, MA 02118, USA; [3]NHLBI Framingham Heart Study, Framingham, MA 01702, USA; [4]Research Computing Services, Information Services & Technology, Boston University, Boston, MA 02215, USA; [5]John P. Hussman Institute for Human Genomics, University of Miami Miller School of Medicine, Miami, FL 33136, USA; [6]The Dr. John T. Macdonald Foundation Department of Human Genetics, University of Miami Miller School of Medicine, Miami, USA, Miami, FL 33136, USA; [7]Department of Biostatistics, Epidemiology, and Informatics, Perelman School of Medicine, Philadelphia, PA 19104, USA; [8]Penn Neurodegeneration Genomics Center, Department of Pathology and Laboratory Medicine, University of Pennsylvania Perelman School of Medicine, Philadelphia, PA 19104, USA; [9]Glenn Biggs Institute for Alzheimer's Disease and Neurodegenerative Diseases, University of Texas Health San Antonio, San Antonio, TX 78229, USA; [10]School of Mathematics and Statistics and KLAS, Northeast Normal University, Changchun, Jilin 130024, China; [11]Department of Biostatistics, University of North Carolina at Chapel Hill, Chapel Hill, NC 27599, USA; [12]Department of Genetics, University of North Carolina at Chapel Hill, Chapel Hill, NC 27599, USA
[13]These authors contributed equally
[14]Further details can be found in the supplemental information
[15]Lead contact
*Correspondence: seuchoi@bu.edu (S.H.C.), xihaoli@unc.edu (X.L.)
https://doi.org/10.1016/j.xhgg.2026.100574.
© 2026 The Author(s). Published by Elsevier Inc. on behalf of American Society of Human Genetics.
This is an open access article under the CC BY license (http://creativecommons.org/licenses/by/4.0/).

ADRD-related association studies. A whole-exome sequencing (WES) study with 17 AD-related traits found that rare coding variations in *RBKS* and *OR7A10* contribute to cognitive performance and protection against left hippocampal atrophy, respectively.[12] Whole-genome sequencing (WGS) ADRD association studies with limited sample sizes have previously been performed to investigate rare variant associations, and these studies found suggestive associations with ADRD in *LAIR1*, *TREM2*, and *PSEN1* genes.[13,14]

Unlike common variants, rare variant analyses require testing an aggregation of variants to maintain statistical power, and methods have been developed to address this power issue.[15–17] Variant-set test for association using annotation information (STAAR) is one of the latest approaches in tackling challenges in rare variant analysis.[18,19] By integrating functional annotation via the Functional Annotation of Variants Online Resources (FAVOR),[20] STAAR efficiently generates functionally interpretable gene-based association results.[18] The key advantage of STAAR lies in its ability to aggregate results across annotation and test sets into a single measure of statistical significance. This makes it particularly valuable for sequencing data from diverse ancestries, where assumptions about rare variants are less stringent. As a result, the implementation of the STAAR framework has been well accepted to explore rare variant associations using WGS data.[21–23] Large-scale sequencing studies such as the Trans-Omics for Precision Medicine (TOPMed) Program of the National Heart, Lung, and Blood Institute (NHLBI), the Alzheimer's Disease Sequencing Project (ADSP), the All of Us Research Project (AoU), and the UK Biobank have generated vast genomic datasets, and the STAAR framework has been applied within each of these studies.[21,23–25] Here, we implement our extensions into the STAAR framework in the context of ADRD using ADSP data, but the updated STAAR framework is relevant to researchers across a broad range of complex diseases and genomic resources.

The ADSP is a collaborative research effort aimed at identifying genetic risk and protective factors for ADRD. The project collects data from diverse individuals from family, case-control, and cohort studies.[26] High-quality WGS data were obtained through a standardized variant-calling pipeline with extensive quality controls.[27,28] In this study, we investigate rare variant associations with ADRD using the STAAR framework in the ADSP release 4 (R4) WGS data. Additionally, we examine potential protective rare genetic variations among cognitively healthy elders within the ADSP. As one of the studies to implement the STAAR analysis pipeline for rare variant association studies with a dichotomous trait, our findings are of substantial importance to understanding the benefits and limitations of this framework. Given the growing interest, our research will serve as a valuable resource for future application of the STAAR framework in studies of dichotomous traits.

Material and methods

Study participants

ADSP includes multiple cohorts, and jointly called WGS data have been generated. A detailed description of the dataset used in this study can be found in the supplemental information. Study participants provided written informed consent per each study's institutional review board (IRB)-approved protocol. This study was conducted under a protocol approved by the Boston University IRB.

The R4 release of the ADSP data used in this study contains 36,361 WGS samples accessed from the National Institute on Aging Genetics of Alzheimer's Disease Data Storage Site (NIAGADS) (NG00067.v10). Genotype-wide quality checks were performed by the Genomic Center for AD (GCAD). Sample-level quality measurements were performed by GCAD, and outliers were excluded by using the mean ± 10 standard deviation of the residual of the following measures: genotype missing rate, singleton rate, heterozygous/homozygous ratio, and transition/transversion ratio. Carriers of clinical variants in *APP*, *PSEN1*, and *PSEN2* were removed from the ADRD samples using the same approach as described in a previous study.[29] Participants from the progressive supranuclear palsy (PSP) and corticobasal degeneration (CBD) cohorts were excluded from our analysis because they did not have ADRD information. To focus on late-onset ADRD and to reduce misclassification in controls, we excluded individuals younger than 55 years. After the exclusions, we have 23,454 samples available for analysis, with 8,697 individuals affected by ADRD and 14,757 control subjects. Utilizing phenotypic data from the Phenotype Harmonization Consortium (PHC), we were able to identify 1,784 cognitively healthy elders and 11,508 general control subjects within our ADSP sample.[30] Multi-allelic variants were excluded from this study. Quality Control (QC) flags in the ADSP files were used in a filtering process to retain high-quality variants as detailed in the supplemental information.

Phenotype determination

The ADSP leverages data from diverse cohorts, each employing independent criteria (autopsy, clinical diagnosis, neuropsychological testing, etc.) to define AD or dementia status. Phenotype data from case-control, family, and the Alzheimer's Disease Neuroimaging Initiative (ADNI) studies were centrally harmonized and provided in three phenotype files. For participants in the ADSP case-control phenotype study, which includes longitudinal data, we defined individuals affected by ADRD as individuals with either prevalent or incident AD. Participants with a baseline ADRD-free status were included as affected individuals if they developed ADRD at a later age, while participants without a diagnosis of ADRD at any point were coded as control subjects. We also included cohorts that provided dementia status rather than AD, including the Longitudinal Aging Study in India-Diagnostic Assessment of Dementia (LASI-DAD) study,[31] which used machine learning to diagnose some participants.[32] In the ADSP family studies, ADRD status was defined in varying ways, including no dementia, definite AD, probable AD, possible AD, family-reported AD, other dementia, family-reported no dementia, and unknown. To harmonize ADRD status among the family studies, individuals with possible, probable, or definite AD were defined as affected individuals, while those individuals classified as no dementia were defined as control

subjects. Participants with family-reported AD, family-reported no dementia, other dementia, and unknown were all recoded as missing for ADRD status and excluded. The ADNI cohort provides information on mild cognitive impairment (MCI) in addition to AD status. Individuals with a current diagnosis of MCI were excluded from the analysis. The PHC cohort, which curated longitudinal ADRD diagnosis data across multiple ADSP cohorts, enabled the identification of cognitively healthy individuals in the R4 WGS data. Cognitively healthy status was defined as having a PHC diagnosis of no dementia with an age last cognitively normal above 85 or a diagnosis of AD or MCI after age 85. General control subjects were defined as individuals diagnosed with AD or MCI before age 85, individuals diagnosed with AD or MCI with missing age of onset, or individuals who were disease free but younger than age 85.

Variant annotation

Rare genetic variants were annotated using the whole-genome functional annotation tool FAVORannotator, which automatically defines gene-based genetic variant sets according to their functional annotation.[20] The FAVORannotator classifies variants into three main categories: coding, non-coding, and non-coding RNA (ncRNA) variants. Coding variants were further categorized into predicted function groups, including putative loss of function (pLoF), disruptive missense (DS), pLoF and DS, missense, and synonymous. Non-coding variants were mapped with information on untranslated regions (UTRs), downstream, upstream, promoters (CAGE and DHS), and enhancers (CAGE and DHS). Variant-level functional scores used principal-component analysis (PCA) to generate multi-dimensional annotation scores. A total of nine annotation principal component (aPC) scores and 3 integrative scores (CADD, LINSIGHT, and FATHMM-XF) were included in the set of annotations as standard output of the FAVORannotator.

PCA and genetic kinship matrix

To account for population structure, we estimated PCs using PC-AiR in the GENESIS package.[33,34] Because PC-AiR calculates ancestry-informative PCs while minimizing the influence of close relatives, this approach was adopted in this dataset, which included a diverse population, ensuring accurate global ancestry inference. For PC calculation, we selected variants with a minor-allele frequency (MAF) of >5%, a call rate of >99%, a GCAD-provided variant flag (VFlag) of 0, and a Robust Unified Hardy-Weinberg Equilbrium (HWE)[35] p value $> 10^{-4}$ and in low linkage disequilibrium (LD) regions ($r^2 < 0.1$). Variants in regions of long-range and high LD were also removed from the datasets before PC calculations.[36]

Since the ADSP includes related participants, we estimated empirical relationships among individuals. PC-Relate, a tool for estimating kinship coefficients, identity-by-descent sharing probabilities, and inbreeding coefficients using genomic data, was applied to construct the genetic kinship matrix.[37] A sparse kinship matrix was generated using the cutoff of fourth-degree relatives (0.022) for computation efficiency. This sparse genetic kinship matrix was incorporated into a generalized mixed-effects model for rare variant analyses.

Rare variant analysis

We conducted analyses using the STAAR framework to perform association tests between rare variants and ADRD status.[18,19,38] *APOE* genotypes were obtained from array genotyping data provided by participating cohorts. For participants without directly genotyped *APOE* data, *APOE* genotypes were determined from the WGS data. Variants with a MAF of less than 1% were included in the analysis. Different models were fit for ADRD and cognitively healthy status to ensure model fitness and statistical power. For the ADRD analysis, we used a logistic mixed-effects model, adjusting for sex, technical sequencing variables (sequencing center and PCR status), *APOE* $\varepsilon 2$ and $\varepsilon 4$ allele counts, and PCs associated with ADRD status. In the cognitively healthy individual analysis, we adjusted for technical sequencing variables, *APOE* $\varepsilon 2$ and $\varepsilon 4$ allele counts, and PCs associated with cognitively healthy status. Relatedness among individuals was adjusted using a genetic kinship matrix.

We set the significance threshold of the rare variant analysis to 1×10^{-7} based on Bonferroni correction for ~20,000 genes across the categories. The STAAR-O p value was used to aggregate p values from the annotation sets for each gene category, and gene-based aggregates with a STAAR-O p value less than the significance threshold were considered statistically significant. Gene categories with a cumulative minor-allele count (cMAC) less than 10 were excluded from the results.[39]

STAARpipeline modifications

To enhance the robustness of the STAAR framework, we modified the STAARpipeline R package by (1) adding the cMAC for each gene aggregate in the output summary files; (2) removing LINSIGHT annotation for coding genetic variants; (3) removing the aggregated Cauchy association test (ACAT-V) p values in generating the STAAR-O p value when gene-based aggregates were formed from ultra-rare variants (MAC < 10), with the cMAC of these ultra-rare variants being less than 10; (4) removing the ACAT-V p values when the cMAC of the ultra-rare variants was equal to the cMAC of all variants in the gene; and (5) decoupling the missense and DS p values when generating the STAAR-O p values for missense genes. Sensitivity analyses were conducted using the modified STAARpipeline R package, with a suggestive significance threshold set at 1×10^{-6}. All statistical analyses were performed using R 4.2.2. A schematic of the analysis is shown in Figure 1.

Results

Characteristics of participants

Our analysis sample of the ADSP WGS data contained 23,454 participants. Among the individuals affected by ADRD (n = 8,697, 37%), the mean baseline age was 75.6 years (SD = 8.6), while ADRD control subjects (n = 14,757, 63%) were slightly younger, with a mean baseline age of 74.3 years (SD = 8.9). We observed more females compared to males in both individuals affected by ADRD and control subjects (Table 1). Between the two *APOE* allele types that we modeled, $\varepsilon 4$ was more prevalent in both individuals affected by ADRD and control subjects, specifically, 51.3% of individuals affected by ADRD carrying at least one $\varepsilon 4$ allele as compared to 24.6% of the control subjects. As expected, more carriers of the $\varepsilon 2$ allele were observed in the ADRD control subjects, with 12.3% of the control subjects vs. 7.7% of the

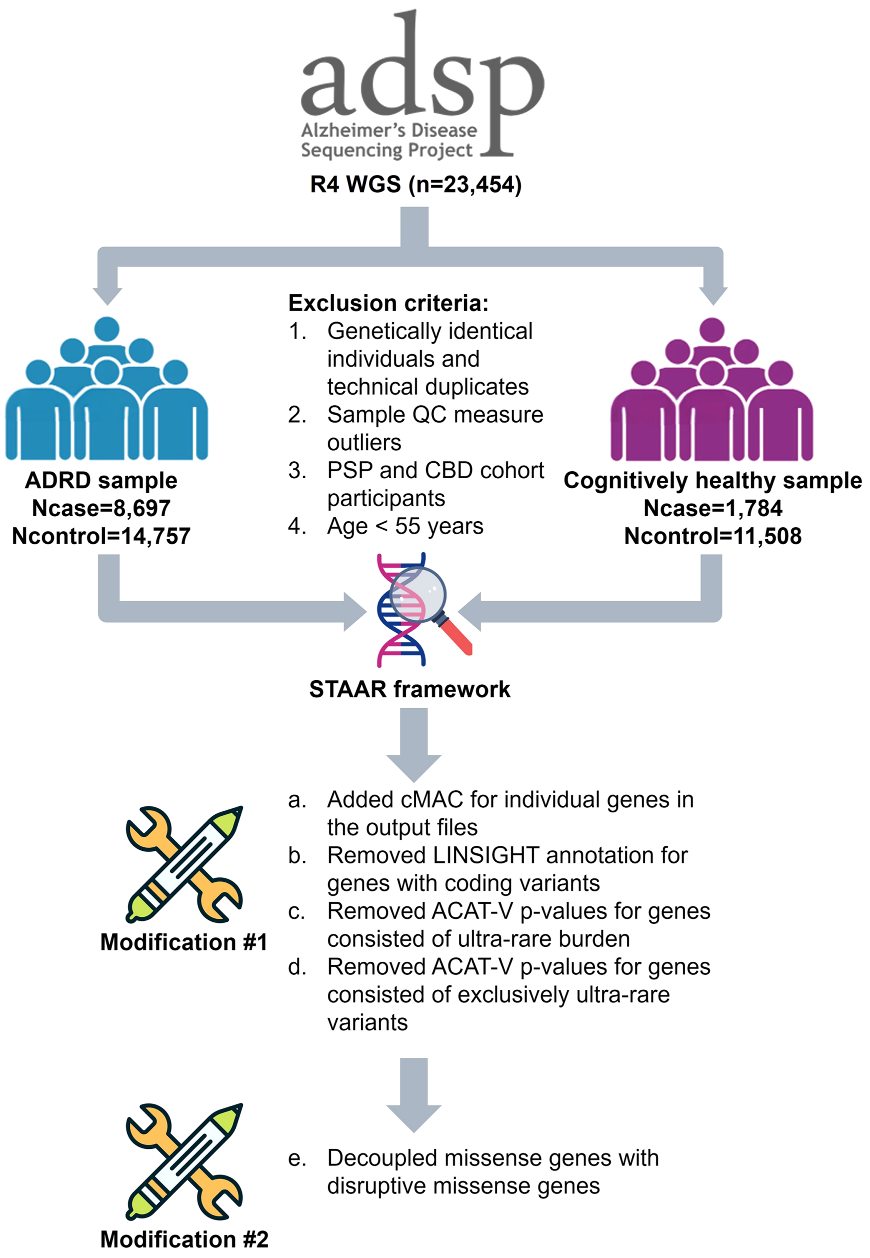

Figure 1. Flow chart of the analysis
A schematic of the analysis. The ADSP R4 WGS sample used in this analysis contains 23,454 participants. Rare variant analyses were conducted using the STAAR framework in the R4 WGS samples for Alzheimer disease and related dementias (ADRD) status and a subset of the R4 WGS samples ($n = 13{,}292$) for cognitively healthy status. We performed two sensitivity analyses after implementing modifications to the STAAR framework in both samples and reported our findings from these analyses. cMAC, cumulative minor-allele count; PSP, progressive supranuclear palsy; CBD, corticobasal degeneration.

affected individuals carrying at least one *ε2* allele (Table 1).

For the cognitively healthy sample ($n = 13{,}292$), individuals with a cognitively healthy status were generally older at baseline and comprised more females and *APOE ε2* carriers as compared to the general control subjects (Table 1). Separate descriptive statistics, including cognitively normal age and age of onset for the cognitively healthy sample, are reported in Table S1. Based on the reported race and ethnicity provided in the ADSP data, our study sample included 2,085 Hispanic White, 209 Hispanic Black, 6,373 Hispanic other/unknown, 7,663 non-Hispanic White, 4,317 non-Hispanic Black, and 2,807 non-Hispanic other/unknown individuals (see supplemental information for details). Using a MAF threshold of 1%, we found 244.6 million bi-allelic variants for the subsequent gene-based tests.

Aggregates of rare variants that are associated with ADRD or cognitively healthy status from the original STAAR framework

Applying the original STAAR framework, we identified 2 genes associated with ADRD status (Table 2; Figures 2A and S1). After adjusting for *APOE*, we observed that pLoF and DS variants in *ZNF200* showed genome-wide statistically significant association with ADRD ($p = 5.05 \times 10^{-8}$). Non-coding variants in *HLA-F* have shown suggestively significant association with ADRD (Table S2). In the cognitively healthy sample, we found statistically significant associations between cognitively healthy status and *METTL26*, *TBX19*, and *CDH22* genes (Table 2; Figure 2B). A few genes of non-coding rare variants and ncRNA also showed significant association with cognitively healthy status, such as *PLXNB2*, *CARD11*, and *LINC01880* (Table S2). However, the functional roles of these genes remain unclear, with limited evidence linking them directly to cognitive measures. Additionally, genes with suggestively significant associations ($p < 1 \times 10^{-6}$) are listed in Tables 2 and S2 as well.

ACAT-V may be sensitive to ultra-rare variant sets

The STAAR framework performs statistical analyses of annotation sets of rare variants using three main approaches: the burden test, the sequence kernel association test (SKAT), and the ACAT-V.[19] ACAT-V has been shown to be robust to the sparsity of causal variants, the directionality of effects, and the choice of weights.[40] With the significant associations found from our analyses, we

Table 1. Descriptive statistics of the ADSP sample

	ADRD sample ($N = 23,454$)		Cognitively healthy sample ($N = 13,292$)	
	ADRD ($n = 8,697$)	Control ($n = 14,757$)	Cognitively healthy ($n = 1,784$)	General control ($n = 11,508$)
Baseline age (years)	75.6 (8.6)	74.3 (8.9)	82.5 (5.3)	71.7 (8.6)
**Sex**				
Female	5,426 (62.4%)	9,329 (63.2%)	1,151 (64.5%)	6,929 (60.2%)
Male	3,271 (37.6%)	5,428 (36.8%)	633 (35.5%)	4,579 (39.8%)
APOE ε2				
0	8,025 (92.3%)	12,946 (87.7%)	1,501 (84.1%)	10,494 (91.2%)
1	652 (7.5%)	1,736 (11.8%)	271 (15.2%)	984 (8.6%)
2	20 (0.2%)	75 (0.5%)	12 (0.7%)	30 (0.3%)
APOE ε4				
0	4,229 (48.6%)	11,119 (75.3%)	1,442 (80.8%)	6,276 (54.5%)
1	3,709 (42.6%)	3,386 (22.9%)	330 (18.5%)	4,288 (37.3%)
2	759 (8.7%)	252 (1.7%)	12 (0.7%)	944 (8.2%)

Genotyped *APOE* allele counts are reported.

next examined which individual annotation test set was driving the overall significance. To understand the exact functional annotation behind the significant signal, we visualized the *p* values from the individual annotation sets for the significant genes (Figure 3). The suggestive significance of *SSAN1*, which is a DS aggregate that includes 3 SNVs totaling 14 alleles, was solely driven by the ACAT-V aPC-conservation set *p* values. Further investigation revealed that only one of the SNPs (rs374374337) was highly significant in the single-variant analysis (Table S3), with only a single alternative allele carrier. ACAT-V integrated single-variant-analysis summary statistics for variants with a MAC of ≥10 and burden test summary statistics for variants with a MAC of <10. However, the burden test result may be sensitive to the low cMAC of *SSAN1* ultra-rare variants (MAC < 10), as the suggestive significance of *SSAN1* is primarily driven by one singleton in cognitively healthy individuals.

Another ACAT-V issue was observed in *LCNL1*, which consists of 2 SNPs totaling 11 alleles (Figure 3). Identical ACAT-V and burden *p* values were obtained for *LCNL1*. This is expected, as the ACAT-V test utilizes the burden test *p* value in its calculation due to the low allele count of *LCNL1* variants. When all variants in an aggregate have a MAC of <10, the cMAC of this gene equals the cMAC of its ultra-rare variants (MAC < 10), causing ACAT-V to generate the same *p* values as the burden test. This redundancy effectively doubles the weighting in the Cauchy approximation process, leading to less accurate results. To improve the robustness of ACAT-V, we applied multiple modifications in the existing STAARpipeline R package, including (1) removing the ACAT-V *p* values from the STAAR-O *p* value calculation when gene-based aggregates were composed of ultra-rare variants (MAC < 10) with a cMAC of <10 and (2) removing the ACAT-V *p* values when the cMAC of the ultra-rare variants was equal to the cMAC of all variants in the gene. Sensitivity analyses were performed to evaluate the impact of these modifications.

LINSIGHT annotation in coding rare variants

Using the *p* value plot, we also examined the significance of individual annotation sets for the *ZNF200* pLoF and DS aggregate (Figure 3). Interestingly, only the LINSIGHT annotation set presented significant signals for *ZNF200*. LINSIGHT is a predictive measure of negative selection at known non-coding sites with inherited diseases, and the LINSIGHT score can accurately predict disease-associated genetic variants located outside protein-coding genes.[41] However, we did not expect LINSIGHT-based set *p* values to drive the significance in known coding variants in *ZNF200* (Table S4). To ensure the accuracy of the rare variant annotation, we removed LINSIGHT annotation sets from all rare coding variant tests in the sensitivity analyses.

Decoupling the DS aggregate from the missense category

After implementing the modifications for ACAT-V and LINSIGHT, we noticed that the *PHLDA1* missense rare variant set has suggestive significance with ADRD (Figures S2 and S3). To determine the putative causal variant(s) within the *PHLDA1* missense aggregate, we performed a leave-one-variant-out analysis to identify the most impactful variant in *PHLDA1*. Surprisingly, none of the 58 tests showed a significant association with ADRD (Figure 4), despite suggestively significant STAAR-O *p* values observed in the Manhattan plot (Figure S2). After

Table 2. Gene-based test results for coding variants in the analyses

Gene	Chr	Category	No. of SNVs	cMAC	STAAR-O
**ADRD sample**					
ZNF200	16	pLoF and DS	7	25	5.05×10^{-8}
TPTE	21	pLoF and DS	4	102	9.05×10^{-7}
**Cognitively healthy sample**					
METTL26	16	pLoF	9	15	3.95×10^{-10}
SAMD14	17	pLoF	2	98	2.85×10^{-7}
TBX19	1	pLoF and DS	17	22	1.24×10^{-8}
LCNL1	9	pLoF and DS	2	11	9.37×10^{-7}
CDON	11	pLoF and DS	22	45	8.99×10^{-7}
TIMELESS	12	pLoF and DS	9	11	9.29×10^{-7}
CDH22	20	pLoF and DS	11	12	3.32×10^{-8}
TBX19	1	DS	14	18	3.16×10^{-7}
SSNA1	9	DS	3	14	3.06×10^{-7}

Genes with significant or suggestive association with ADRD or cognitively healthy status are shown. Chr, chromosome; SNV, single-nucleotide variant; cMAC, cumulative minor-allele count; pLoF, putative loss of function; DS, disruptive missense.

further investigation, we found that the STAAR function combines missense STAAR-O p value results with DS set p values for the same gene. The significance of *PHLDA1* missense aggregate was entirely driven by two ultra-rare DS variants (chr12:76030981A>G and rs551935657), which were already included in the missense aggregate set. Additionally, the cMAC of these two variants is less than 10, indicating that the significance of the *PHLDA1* missense aggregate may be inconclusive. To address this issue, we decoupled the missense and DS results in the STAAR-O p value calculation, keeping the results separated by the predicted variant function categories.

Sensitivity analyses

Overall, we implemented multiple modifications to the existing STAAR and STAARpipeline R packages to enhance analytical robustness: we (1) added the cMAC for each gene aggregate in the output summary files; (2) removed LINSIGHT annotation for coding genetic variants; (3) removed the ACAT-V p values from STAAR-O p value calculation when gene-based aggregates were formed from ultra-rare variants (MAC < 10) with a cMAC of <10; (4) removed ACAT-V p values when the cMAC of the ultra-rare variants was equal to the cMAC of all variants in the gene; and (5) decoupled the missense and DS p values when generating the STAAR-O p values for missense genes (Figure 1). Sensitivity analyses using the modified STAAR framework revealed that the previously significant coding variant aggregates were no longer significant in the ADRD analysis (Figures 2C and 2D).

In non-coding gene-based tests, however, the *HLA-F* upstream aggregate showed suggestive significance for association with ADRD (Figures S4C and S5; Table S5). *HLA-F* belongs to the major histocompatibility complex (MHC) region, and it requires meticulous interpretation due to the complexity of the region in genetic association studies. In the analysis of cognitively healthy individuals, *TBX19*, *PLXNB2*, *CARD11*, and *LINC01880* remained significantly associated with cognitively healthy status in the sensitivity analysis (Tables 3 and S5; Figure S4D). One family study reported that a *TBX19* gene mutation was found in patients with isolated adrenocorticotropic hormone (ACTH) deficiency, which causes cognitive impairment, particularly in the neonatal period.[42] Our findings allude to the potential protective role for cognition function decline in these genes. Modifications effectively remedied the issues found in our results and improved the robustness of the STAAR framework.

Discussion

Rare genetic variants play a key role in the underlying genetic mechanism of ADRD, yet evidence linking rare variants to ADRD remains limited. The recently published STAAR framework offers a powerful, scalable, and accessible approach for performing annotation-based omnibus tests in rare variant analysis. The ADSP is the largest genetic consortium with WGS data from diverse populations of individuals with ADRD status. Prior ADSP publications have identified and confirmed genetic associations with ADRD, but the evidence supporting associations with rare genetic variations is still limited.[13,14] To address this gap in knowledge, we leveraged functional annotation in rare variant analysis by applying the STAAR framework to gene-based tests in the ADSP R4 WGS data. This approach identified a few biologically plausible gene

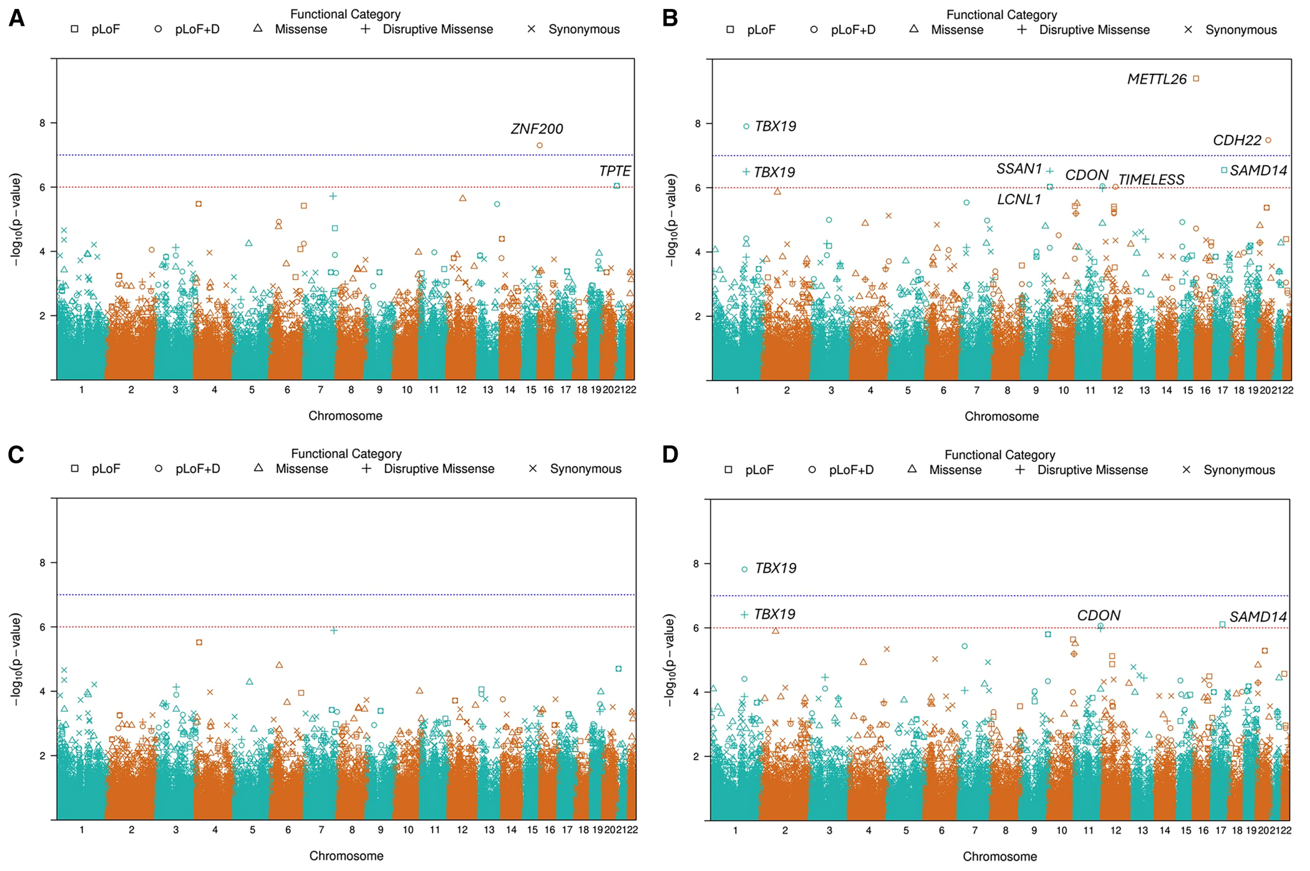

Figure 2. Manhattan plots of gene-based tests for coding variants in the ADRD and cognitively healthy samples before and after implementing modification #2

Manhattan plots show gene-based test results for coding variants. The blue reference line represents the genome-wide significance threshold of 1×10^{-7}, and the red reference line represents a suggestively significant threshold of 1×10^{-6}.

(A and C) Gene-based test results before and after applying modification #2 in the ADRD sample. *TPTE* was annotated as both a pLoF and DS and a pLoF gene in (A).

(B and D) Gene-based test results before and after applying modification #2 in the cognitively healthy sample. The *y* axis shows the −log10(*p* value), while the *x* axis represents the location of genes on chromosomes.

targets for ADRD or cognitively healthy status using both coding and non-coding rare variant aggregates. However, some of these findings may be sensitive to certain characteristics of rare variant aggregates due to the limitations in the framework. To enhance statistical robustness, we proposed and implemented modifications to mitigate the issues.

One of the challenges in rare variant analysis is adequately combining different test results from widely used methods such as burden tests and SKAT. Assumptions of these tests often involve the choice of weights, effect sizes, and direction of causal variants, as well as the number of causal variants. SKAT-O was developed as an omnibus test that combines both burden tests and SKAT, offering greater robustness to varying directionality in effect sizes.[43] However, the power of SKAT-O can be severely impacted by the sparsity of causal variants in the genome.[44] Additionally, combining multiple tests with SKAT-O can be computationally expensive because of the correlation among the tests. In contrast, ACAT-V has some distinctive benefits. Unlike conventional tests, ACAT-V requires minimal assumptions and relies only on the *p* values, making it computationally efficient.[40] Utilizing the approximation from a Cauchy distribution, ACAT-V does not require LD or a population reference panel to construct the set-level-based test statistics. Instead, ACAT-V treats *p* values as independent variables, due to a key property of the Cauchy distribution.[45] Additionally, if the set-based tests were effectively controlled for genetic relatedness and/or population structure, ACAT-V results will retain the same adjustment in their calculation automatically.[40]

In our analyses, we observed unique features of the ACAT-V that had not been previously discussed. Notably, ACAT-V combines burden test *p* values for ultra-rare variants (MAC < 10) with single-variant-analysis *p* values for rare variants (MAC ≥ 10) into a single significance measure.[40] The STAAR framework incorporated this method, but the results are reliable only when the cMACs of ultra-rare variants within a gene aggregate exceed 10. For example, the *SSAN1* pLoF and DS aggregate only consists of 2 singletons and 1 rare variant with 14 alleles. A

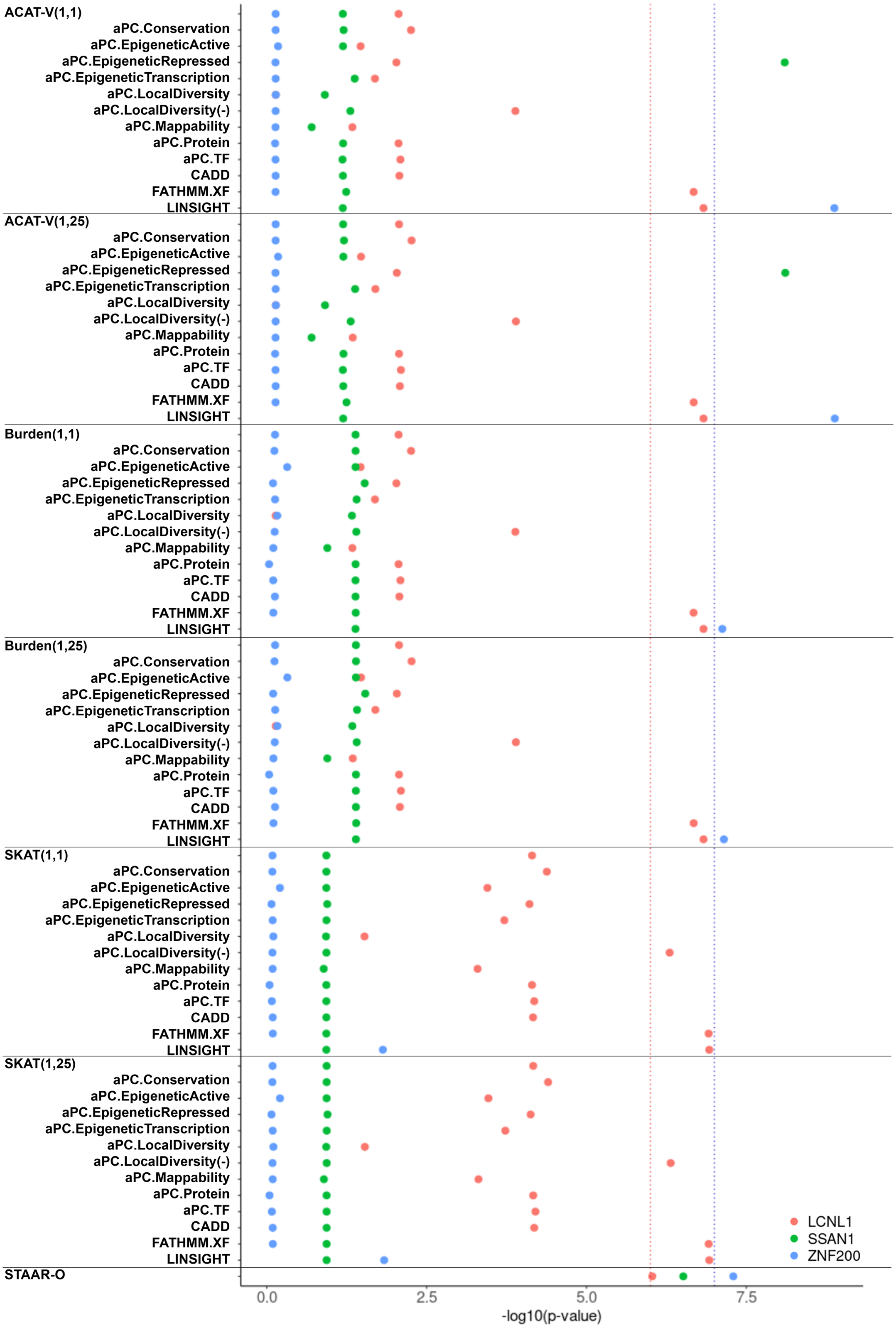

Figure 3. Annotation set *p* value plots for selected genes
Scatterplots showing the annotation set *p* values of ACAT-V, burden, and SKAT for *SSAN1*, *LCNL1*, and *ZNF200*. The blue reference line represents the genome-wide significance threshold of 1×10^{-7}, and the red reference line shows the suggestively significant threshold of 1×10^{-6}. Color dots demonstrate *LCNL1* (red), *SSAN1* (green), and *ZNF200* (blue) *p* values, respectively. The *x* axis shows the $-\log10(p \text{ value})$, while the *y* axis represents different annotation sets.

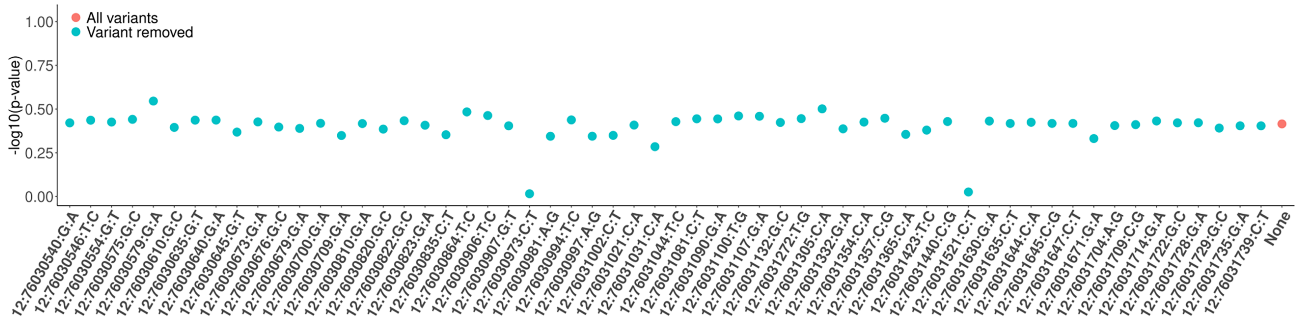

Figure 4. Leave-one-variant-out analysis for the *PHLDA1* missense aggregate
Scatterplot demonstrates the STAAR-O *p*-values in the leave-one-variant-out analysis for the *PHLDA1* missense aggregate. Each variant was removed in the aggregate, and we performed 59 individual tests after implementing modification #1 in the ADRD sample. The red dot shows the *p* value without removing any variants in the aggregate. Blue dots represent the *p* values in the leave-one-out analysis. The *y* axis shows the −log10(*p* value), while the *x* axis represents the exact variant removed in each tested aggregate.

burden test was not appropriate in this scenario because the cMAC of the two singletons is far below the commonly used cutoff of 10 (Table S3).[39,46,47] Additionally, ACAT-V test sets can share identical *p* values with the burden test sets if the gene aggregate consisted exclusively of ultra-rare variants. This redundancy may inflate the statistical weights in the STAAR-O *p* value calculation, as the same set of *p* values contributes twice to the final statistical measure. As demonstrated by *LCNL1* in Figure 3, the suggestive significance disappeared after our modifications in sensitivity analyses (Table 3; Figure 2D). To address these issues, we implemented adequate filtering approaches in the STAAR framework. We installed a MAC filter and a cMAC filter for ultra-rare variants in each gene aggregate, which effectively removed the ACAT-V *p* values from the STAAR-O *p* value calculation when gene aggregates consisted of ultra-rare variants with a cMAC of <10. We also excluded ACAT-V *p* values when a gene aggregate consists solely of ultra-rare variants. With these modifications, we believe that the statistical robustness of the STAAR framework was improved.

LINSIGHT combines a generalized linear model for functional genomic data with a probabilistic evolution model to identify non-coding variants associated with inherited diseases.[41] A higher score in LINSIGHT corresponds to a higher probability that the non-coding variants are constrained to mutations. A previous report showed that rs7304782, a variant in the first intron of the *MTRFR* region, may contribute to schizophrenia risk by regulating *OGFOD2* expression in human brain tissues.[48] However, the highly significant LINSIGHT-annotated burden, SKAT, and ACAT *p* values for *ZNF200* appear inconsistent with the intended function of the LINSIGHT score (Figure 3). In addition, we could not find any published evidence suggestive of the LINSIGHT score in protein-coding variants. To address this discrepancy, we removed LINSIGHT annotation test sets from all coding variant aggregates when combining the individual test set results in the STAAR framework.

STAAR framework integrates the FAVOR annotation database for genome-wide variant information, including gene name, position, and function.[20] Missense variants were defined by non-synonymous status from the GENCODE exonic category, while DS status was predicted using a meta-analytic support vector machine (MetaSVM) algorithm.[49,50] A common strategy in rare variant analysis is to construct separate missense and DS variant aggregates for individual genes, and the STAAR framework adopted this approach. However, the calculation of STAAR-O *p* values for missense genes was based on the annotation set *p* values of both the missense and DS aggregates of the same gene. This was unexpected, as the DS variants were already included in the missense aggregates, leading to overlapping variants in both functional sets. For example, we observed a suggestively significant signal in *PHLDA1*, yet the leave-one-variant-out analysis showed that none of the aggregates reached the suggestive significance threshold (Figures S2 and 4). We believe that the missense gene-based results should be decoupled from the DS results in generating the STAAR-O *p* value for the same gene. After separating missense with DS results for *PHLDA1*, we no longer observed suggestive significance in the sensitivity analyses.

The main focus of this study is on introducing computational refinements in the STAAR framework to produce robust results in association analyses of rare variants for dichotomous traits. We demonstrated the need for and utility of these refinements by applying them to publicly available ADSP WGS data using two distinct dichotomous phenotypes: ADRD status and cognitively healthy elder status. We recognize some limitations of these phenotype definitions. The exclusion of individuals younger than 55 years of age reduces misclassification of controls but may also limit power to identify rare variants driving Mendelian and/or complex forms of this disease in the 639 individuals affected by ADRD among those younger than 55 years in the ADSP R4 WGS data. Other study designs that implement distinct age thresholds for affected individuals and control subjects may

Table 3. Gene-based test results for coding variants in the analyses after implementing modification #2

Gene	Chr	Category	No. of SNVs	cMAC	STAAR-O
**Cognitively healthy sample**					
SAMD14	17	pLoF	2	98	7.77×10^{-7}
TBX19	1	pLoF and DS	17	22	1.53×10^{-8}
CDON	11	pLoF and DS	22	45	8.53×10^{-7}
TBX19	1	DS	14	18	3.91×10^{-7}

Genes with significant or suggestive association with ADRD or cognitively healthy status are shown. Chr, chromosome; SNV, single-nucleotide variant; cMAC, cumulative minor-allele count; pLoF, putative loss of function; DS, disruptive missense.

provide additional information regarding ADRD genetic architecture. However, given our methodological focus, we consider these analyses beyond the scope of the current work and are better suited to larger-sample-size datasets.

The STAAR framework was initially applied to quantitative traits such as blood lipid levels, inflammation biomarkers, height, and glycemic traits.[19,21,23–25] To date, there have been limited studies investigating rare genetic variant associations with dichotomous traits. This study was based on the ADSP R4 WGS data, but we have also observed the same issues in other datasets.[51] Hence, the modifications proposed here are likely to have widespread utility in the study of rare variants in association with dichotomous traits.

Conclusion

We systematically evaluated the association of rare genetic variation with ADRD in 23,455 individuals using the ADSP R4 WGS data. After final analyses, we did not observe significant associations with ADRD. Additionally, we investigated potentially protective rare variants associated with cognitively healthy status using a subset of the ADSP R4 WGS data. We identified that *TBX19*, *PLXNB2*, *CARD11*, and *LINC01880* are significantly associated with a cognitively healthy status. Further replication studies, which are beyond the scope of this method-focused article, are needed to confirm these association findings. Our main finding is that when implementing the original STAAR framework, we observed potential spurious associations in multiple genes due to issues related to ACAT-V, LINSIGHT annotation, and other factors. To address these issues, we proposed modifications to mitigate their effects while maintaining statistical power and robustness. We recommend the use of this modified STAARpipeline for analysis of dichotomous traits.

Data and code availability

ADSP WGS data (NG00067.v10) and PHC phenotypic data (NG00067.v12) are available through the NIAGADS (https://www.niagads.org/). The code generated during this study is available at GitHub: https://github.com/DanielDYWang/STAARpipeline_new.

Acknowledgments

Nancy Heard-Costa is supported by grants 75N92025D00012 and U01AG058589. Eden Martin is supported by U01AG058654. Adam Naj is supported by U01AG058654, RF1AG060472, U54AG052427, U01AG032984, and U24AG041689. Brian W. Kunkle is supported by U01AG076482, U01AG066767, U01AG058654, U01AG057659, and U01AG062943. Gina M. Peloso reports grant support from U01AG058589. Xihao Li is supported by R01AG085581. Seung Hoan Choi, Anita L. DeStefano, and Dongyu Wang are supported by U01AG058589 and U01AG068221. See the supplemental information for details. Data used in preparation of this article were obtained from the ADNI database (adni.loni.usc.edu). As such, the investigators within the ADNI contributed to the design and implementation of ADNI and/or provided data but did not participate in the analysis or writing of this report. A complete listing of ADNI investigators can be found at http://adni.loni.usc.edu/wp-content/uploads/how_to_apply/ADNI_Acknowledgement_List.pdf.

Declaration of interests

The authors declare no competing interests.

Supplemental information

Supplemental information can be found online at https://doi.org/10.1016/j.xhgg.2026.100574.

Web resources

Modified STAARpipeline R package, https://github.com/DanielDYWang/STAARpipeline_new

STAARpipeline R package, https://github.com/xihaoli/STAARpipeline

Received: June 30, 2025
Accepted: January 13, 2026

References

1. Rajan, K.B., Weuve, J., Barnes, L.L., McAninch, E.A., Wilson, R.S., and Evans, D.A. (2021). Population Estimate of People with Clinical AD and Mild Cognitive Impairment in the United States (2020–2060). Alzheimer's Dement. *17*, 1966–1975. https://doi.org/10.1002/ALZ.12362.
2. Focus on Alzheimer’s Disease and Related Dementias. National Institute of Neurological Disorders and Stroke.

https://www.ninds.nih.gov/current-research/focus-disorders/focus-alzheimers-disease-and-related-dementias.
3. Chouraki, V., and Seshadri, S. (2014). Genetics of Alzheimer's disease. Adv. Genet. *87*, 245–294. https://doi.org/10.1016/B978-0-12-800149-3.00005-6.
4. Karlsson, I.K., Escott-Price, V., Gatz, M., Hardy, J., Pedersen, N.L., Shoai, M., and Reynolds, C.A. (2022). Measuring heritable contributions to Alzheimer's disease: polygenic risk score analysis with twins. Brain Commun. *4*, fcab308. https://doi.org/10.1093/BRAINCOMMS/FCAB308.
5. Wightman, D.P., Jansen, I.E., Savage, J.E., Shadrin, A.A., Bahrami, S., Holland, D., Rongve, A., Børte, S., Winsvold, B.S., Drange, O.K., et al. (2021). A genome-wide association study with 1,126,563 individuals identifies new risk loci for Alzheimer's disease. Nat. Genet. *53*, 1276–1282. https://doi.org/10.1038/s41588-021-00921-z.
6. Andrews, S.J., Renton, A.E., Fulton-Howard, B., Podlesny-Drabiniok, A., Marcora, E., and Goate, A.M. (2023). The complex genetic architecture of Alzheimer's disease: novel insights and future directions. EBioMedicine *90*, 104511. https://doi.org/10.1016/j.ebiom.2023.104511.
7. World Health Organization. Dementia. https://www.who.int/en/news-room/fact-sheets/detail/dementia.
8. Bellenguez, C., Küçükali, F., Jansen, I.E., Kleineidam, L., Moreno-Grau, S., Amin, N., Naj, A.C., Campos-Martin, R., Grenier-Boley, B., Andrade, V., et al. (2022). New insights into the genetic etiology of Alzheimer's disease and related dementias. Nat. Genet. *54*, 412–436. https://doi.org/10.1038/s41588-022-01024-z.
9. Khani, M., Gibbons, E., Bras, J., and Guerreiro, R. (2022). Challenge accepted: uncovering the role of rare genetic variants in Alzheimer's disease. Mol. Neurodegener. *17*, 3–15. https://doi.org/10.1186/S13024-021-00505-9.
10. Marx, V. (2023). Method of the year: long-read sequencing. Nat. Methods *20*, 6–11. https://doi.org/10.1038/s41592-022-01730-w.
11. Hiatt, S.M., Lawlor, J.M.J., Handley, L.H., Latner, D.R., Bonnstetter, Z.T., Finnila, C.R., Thompson, M.L., Boston, L.B., Williams, M., Nunez, I.R., et al. (2024). Long-read genome sequencing and variant reanalysis increase diagnostic yield in neurodevelopmental disorders. Genome Res *34*, 1747–1762. https://doi.org/10.1101/GR.279227.124.
12. Küçükali, F., Neumann, A., Van Dongen, J., De Pooter, T., Joris, G., De Rijk, P., Ohlei, O., Dobricic, V., Bos, I., Vos, S.J.B., et al. (2023). Whole-exome rare-variant analysis of Alzheimer's disease and related biomarker traits. Alzheimer's Dement. *19*, 2317–2331. https://doi.org/10.1002/ALZ.12842.
13. Wang, Y., Sarnowski, C., Lin, H., Pitsillides, A.N., Heard-Costa, N.L., Choi, S.H., Wang, D., Bis, J.C., Blue, E.E., Alzheimer's Disease Neuroimaging Initiative ADNI, et al. (2024). Key variants via the Alzheimer's Disease Sequencing Project whole genome sequence data. Alzheimer's Dement. *20*, 3290–3304. https://doi.org/10.1002/ALZ.13705.
14. Lee, W.-P., Choi, S.H., Shea, M.G., Cheng, P.-L., Dombroski, B.A., Pitsillides, A.N., Heard-Costa, N.L., Wang, H., Bulekova, K., Kuzma, A.B., et al. (2024). Association of common and rare variants with Alzheimer's disease in more than 13,000 diverse individuals with whole-genome sequencing from the Alzheimer's Disease Sequencing Project. Alzheimer's Dement. *20*, 8470–8483. https://doi.org/10.1002/ALZ.14283.
15. Lee, S., Emond, M.J., Bamshad, M.J., Barnes, K.C., Rieder, M.J., Nickerson, D.A., NHLBI GO Exome Sequencing Project—ESP Lung Project Team, Christiani, D.C., Wurfel, M.M., and Lin, X. (2012). Optimal Unified Approach for Rare-Variant Association Testing with Application to Small-Sample Case-Control Whole-Exome Sequencing Studies. Am. J. Hum. Genet. *91*, 224–237. https://doi.org/10.1016/J.AJHG.2012.06.007.
16. Zhou, W., Bi, W., Zhao, Z., Dey, K.K., Jagadeesh, K.A., Karczewski, K.J., Daly, M.J., Neale, B.M., and Lee, S. (2022). SAIGE-GENE+ improves the efficiency and accuracy of set-based rare variant association tests. Nat. Genet. *54*, 1466–1469. https://doi.org/10.1038/s41588-022-01178-w.
17. Chen, W., Coombes, B.J., and Larson, N.B. (2022). Recent advances and challenges of rare variant association analysis in the biobank sequencing era. Front. Genet. *13*, 1014947. https://doi.org/10.3389/fgene.2022.1014947.
18. Li, X., Li, Z., Zhou, H., Gaynor, S.M., Liu, Y., Chen, H., Sun, R., Dey, R., Arnett, D.K., Aslibekyan, S., et al. (2020). Dynamic incorporation of multiple in silico functional annotations empowers rare variant association analysis of large whole genome sequencing studies at scale. Nat. Genet. *52*, 969–983. https://doi.org/10.1038/S41588-020-0676-4.
19. Li, Z., Li, X., Zhou, H., Gaynor, S.M., Selvaraj, M.S., Arapoglou, T., Quick, C., Liu, Y., Chen, H., Sun, R., et al. (2022). A framework for detecting noncoding rare variant associations of large-scale whole-genome sequencing studies. Nat. Methods *19*, 1599–1611. https://doi.org/10.1038/S41592-022-01640-X.
20. Zhou, H., Arapoglou, T., Li, X., Li, Z., Zheng, X., Moore, J., Asok, A., Kumar, S., Blue, E.E., Buyske, S., et al. (2023). FAVOR: functional annotation of variants online resource and annotator for variation across the human genome. Nucleic Acids Res. *51*, D1300–D1311. https://doi.org/10.1093/NAR/GKAC966.
21. Hawkes, G., Beaumont, R.N., Li, Z., Mandla, R., Li, X., Albert, C.M., Arnett, D.K., Ashley-Koch, A.E., Ashrani, A.A., Barnes, K.C., et al. (2024). Whole-genome sequencing in 333,100 individuals reveals rare non-coding single variant and aggregate associations with height. Nat. Commun. *15*, 8549. https://doi.org/10.1038/s41467-024-52579-w.
22. Feofanova, E.V., Brown, M.R., Alkis, T., Manuel, A.M., Li, X., Tahir, U.A., Li, Z., Mendez, K.M., Kelly, R.S., Qi, Q., et al. (2023). Whole-Genome Sequencing Analysis of Human Metabolome in Multi-Ethnic Populations. Nat. Commun. *14*, 3111–3112. https://doi.org/10.1038/s41467-023-38800-2.
23. Selvaraj, M.S., Li, X., Li, Z., Pampana, A., Zhang, D.Y., Park, J., Aslibekyan, S., Bis, J.C., Brody, J.A., Cade, B.E., et al. (2022). Whole genome sequence analysis of blood lipid levels in >66,000 individuals. Nat. Commun. *13*, 5995. https://doi.org/10.1038/s41467-022-33510-7.
24. Gardner, E.J., Kentistou, K.A., Stankovic, S., Lockhart, S., Wheeler, E., Day, F.R., Kerrison, N.D., Wareham, N.J., Langenberg, C., O'Rahilly, S., et al. (2022). Damaging missense variants in IGF1R implicate a role for IGF-1 resistance in the etiology of type 2 diabetes. Cell Genom. *2*, 100208. https://doi.org/10.1016/J.XGEN.2022.100208.
25. Jiang, M.Z., Gaynor, S.M., Li, X., Van Buren, E., Stilp, A., Buth, E., Wang, F.F., Manansala, R., Gogarten, S.M., Li, Z., et al. (2024). Whole genome sequencing based analysis of

inflammation biomarkers in the Trans-Omics for Precision Medicine (TOPMed) consortium. Hum. Mol. Genet. *33*, 1429–1441. https://doi.org/10.1093/HMG/DDAE050.

26. Beecham, G.W., Bis, J.C., Martin, E.R., Choi, S.H., DeStefano, A.L., Van Duijn, C.M., Fornage, M., Gabriel, S.B., Koboldt, D.C., Larson, D.E., et al. (2017). The Alzheimer's Disease Sequencing Project: Study design and sample selection. Neurol. Genet. *3*, e194. https://doi.org/10.1212/NXG.0000000000000194.
27. Leung, Y.Y., Valladares, O., Chou, Y.F., Lin, H.J., Kuzma, A.B., Cantwell, L., Qu, L., Gangadharan, P., Salerno, W.J., Schellenberg, G.D., et al. (2019). VCPA: genomic variant calling pipeline and data management tool for Alzheimer's Disease Sequencing Project. Bioinformatics *35*, 1768–1770. https://doi.org/10.1093/BIOINFORMATICS/BTY894.
28. Naj, A.C., Lin, H., Vardarajan, B.N., White, S., Lancour, D., Ma, Y., Schmidt, M., Sun, F., Butkiewicz, M., Bush, W.S., et al. (2019). Quality control and integration of genotypes from two calling pipelines for whole genome sequence data in the Alzheimer's disease sequencing project. Genomics *111*, 808–818. https://doi.org/10.1016/J.YGENO.2018.05.004.
29. Wang, D., Scalici, A., Wang, Y., Lin, H., Pitsillides, A., Heard-Costa, N., Cruchaga, C., Ziegemeier, E., Bis, J.C., Fornage, M., et al. (2025). Frequency of variants in Mendelian Alzheimer's disease genes within the Alzheimer's Disease Sequencing Project. J. Alzheimers Dis. *104*, 841–851. https://doi.org/10.1177/13872877251320375.
30. Hohman, T.J. (2023). ADSP Phenotype Harmonization Consortium. Alzheimer's Dement. *19*, e077713. https://doi.org/10.1002/ALZ.077713.
31. Lee, J., Banerjee, J., Khobragade, P.Y., Angrisani, M., and Dey, A.B. (2019). LASI-DAD study: a protocol for a prospective cohort study of late-life cognition and dementia in India. BMJ Open *9*, e030300. https://doi.org/10.1136/BMJOPEN-2019-030300.
32. Jin, H., Chien, S., Meijer, E., Khobragade, P., and Lee, J. (2021). Learning From Clinical Consensus Diagnosis in India to Facilitate Automatic Classification of Dementia: Machine Learning Study. JMIR Ment. Health *8*, e27113. https://doi.org/10.2196/27113.
33. Conomos, M.P., Miller, M.B., and Thornton, T.A. (2015). Robust inference of population structure for ancestry prediction and correction of stratification in the presence of relatedness. Genet. Epidemiol. *39*, 276–293. https://doi.org/10.1002/GEPI.21896.
34. Gogarten, S.M., Sofer, T., Chen, H., Yu, C., Brody, J.A., Thornton, T.A., Rice, K.M., and Conomos, M.P. (2019). Genetic association testing using the GENESIS R/Bioconductor package. Bioinformatics *35*, 5346–5348. https://doi.org/10.1093/BIOINFORMATICS/BTZ567.
35. Kwong, A.M., Blackwell, T.W., Lefaive, J., De Andrade, M., Barnard, J., Barnes, K.C., Blangero, J., Boerwinkle, E., Burchard, E.G., Cade, B.E., et al. (2021). Robust, flexible, and scalable tests for Hardy–Weinberg equilibrium across diverse ancestries. Genetics *218*, iyab044. https://doi.org/10.1093/GENETICS/IYAB044.
36. Price, A.L., Weale, M.E., Patterson, N., Myers, S.R., Need, A.C., Shianna, K.V., Ge, D., Rotter, J.I., Torres, E., Taylor, K.D., et al. (2008). Long-Range LD Can Confound Genome Scans in Admixed Populations. Am. J. Hum. Genet. *83*, 132–139. https://doi.org/10.1016/J.AJHG.2008.06.005.
37. Conomos, M.P., Laurie, C.A., Stilp, A.M., Gogarten, S.M., McHugh, C.P., Nelson, S.C., Sofer, T., Fernández-Rhodes, L., Justice, A.E., Graff, M., et al. (2016). Genetic Diversity and Association Studies in US Hispanic/Latino Populations: Applications in the Hispanic Community Health Study/Study of Latinos. Am. J. Hum. Genet. *98*, 165–184. https://doi.org/10.1016/J.AJHG.2015.12.001.
38. (2022). STAARpipeline: an all-in-one rare-variant tool for biobank-scale whole-genome sequencing data. Nat. Methods *19*, 1532–1533. https://doi.org/10.1038/s41592-022-01641-w.
39. Bis, J.C., Jian, X., Kunkle, B.W., Chen, Y., Hamilton-Nelson, K.L., Bush, W.S., Salerno, W.J., Lancour, D., Ma, Y., Renton, A.E., et al. (2018). Whole exome sequencing study identifies novel rare and common Alzheimer's-Associated variants involved in immune response and transcriptional regulation. Mol. Psychiatry *25*, 1859–1875. https://doi.org/10.1038/s41380-018-0112-7.
40. Liu, Y., Chen, S., Li, Z., Morrison, A.C., Boerwinkle, E., and Lin, X. (2019). ACAT: A Fast and Powerful p Value Combination Method for Rare-Variant Analysis in Sequencing Studies. Am. J. Hum. Genet. *104*, 410–421. https://doi.org/10.1016/J.AJHG.2019.01.002.
41. Huang, Y.F., Gulko, B., and Siepel, A. (2017). Fast, scalable prediction of deleterious noncoding variants from functional and population genomic data. Nat. Genet. *49*, 618–624. https://doi.org/10.1038/NG.3810.
42. Charnay, T., Mougel, G., Amouroux, C., Gueorguieva, I., Joubert, F., Pertuit, M., Reynaud, R., Barlier, A., Brue, T., and Saveanu, A. (2022). A novel TBX19 gene mutation in patients with isolated ACTH deficiency from distinct families with a common geographical origin. Front. Endocrinol. *13*, 1080649. https://doi.org/10.3389/FENDO.2022.1080649.
43. Lee, S., Wu, M.C., and Lin, X. (2012). Optimal tests for rare variant effects in sequencing association studies. Biostatistics *13*, 762–775. https://doi.org/10.1093/BIOSTATISTICS/KXS014.
44. Barnett, I., Mukherjee, R., and Lin, X. (2017). The Generalized Higher Criticism for Testing SNP-Set Effects in Genetic Association Studies. J. Am. Stat. Assoc. *112*, 64–76. https://doi.org/10.1080/01621459.2016.1192039.
45. Liu, Y., and Xie, J. (2020). Cauchy combination test: a powerful test with analytic p-value calculation under arbitrary dependency structures. J. Am. Stat. Assoc. *115*, 393–402. https://doi.org/10.1080/01621459.2018.1554485.
46. Holstege, H., Hulsman, M., Charbonnier, C., Grenier-Boley, B., Quenez, O., Grozeva, D., van Rooij, J.G.J., Sims, R., Ahmad, S., Amin, N., et al. (2022). Exome sequencing identifies rare damaging variants in ATP8B4 and ABCA1 as risk factors for Alzheimer's disease. Nat. Genet. *54*, 1786–1794. https://doi.org/10.1038/s41588-022-01208-7.
47. Pillalamarri, V., Shi, W., Say, C., Yang, S., Lane, J., Guallar, E., Pankratz, N., and Arking, D.E. (2023). Whole-exome sequencing in 415,422 individuals identifies rare variants associated with mitochondrial DNA copy number. HGG Adv. *4*, 100147. https://doi.org/10.1016/j.xhgg.2022.100147.
48. Ma, C., Li, Y., Li, X., Liu, J., and Luo, X.J. (2020). Identification of a functional SNP rs7304782 at schizophrenia risk locus 12q24.31 and validation of its association with schiz ophrenia in Chinese populations. Psychiatry Res. *294*, 113491. https://doi.org/10.1016/J.PSYCHRES.2020.113491.

49. Mudge, J.M., Carbonell-Sala, S., Diekhans, M., Martinez, J.G., Hunt, T., Jungreis, I., Loveland, J.E., Arnan, C., Barnes, I., Bennett, R., et al. (2025). GENCODE 2025: reference gene annotation for human and mouse. Nucleic Acids Res. *53*, D966–D975. https://doi.org/10.1093/NAR/GKAE1078.

50. Dong, C., Wei, P., Jian, X., Gibbs, R., Boerwinkle, E., Wang, K., and Liu, X. (2015). Comparison and integration of deleteriousness prediction methods for nonsynonymous SNVs in whole exome sequencing studies. Hum. Mol. Genet. *24*, 2125–2137. https://doi.org/10.1093/HMG/DDU733.

51. Wang, D., Choi, S.H., Abbruzzese, S., Rosser, M.A., Bis, J.C., Fornage, M., Boerwinkle, E., Satizabal, C.L., Psaty, B.M., Lopez, O.L., et al. (2024). Whole Genome Sequencing Analysis of Cognitively Wellderly Individuals Identifies Potential Protective Genetic Variants for Alzheimer's Disease. Alzheimer's Dement. *20*, e088478. https://doi.org/10.1002/ALZ.088478.

HGGA, Volume 7

Supplemental information

Application of the STAAR framework in detecting rare variant associations with Alzheimer disease and related dementias: Insights and implications

Dongyu Wang, Sabrina Abbruzzese, Nancy Heard-Costa, Andy Rampersaud, Eden Martin, Adam Naj, Bilcag Akgun, Brian Kunkle, Sudha Seshadri, Gina Peloso, The Alzheimer's Disease Neuroimaging Initiative, The Alzheimer's Disease Sequencing Project, Anita L. DeStefano, Zilin Li, Xihao Li, and Seung Hoan Choi

Supplemental Information

Supplemental Methods

ADSP Sample Description

snd10000-Alzheimer’s Disease Sequencing Project (ADSP) Discovery

The initial phase of the ADSP research plan is called the Discovery Phase. Samples were selected from well-characterized study cohorts of individuals with or without an AD diagnosis and the presence or absence of known risk factor genes. The ADSP generated three sets of genome sequence data for these samples as part of the Discovery Phase: (1) WGS for 584 samples from 113 multiplex families (two or more affected per family), (2) Whole Exome Sequence (WES) for 5,096 AD cases and 4,965 controls, and (3) WES of an Enriched sample set comprised of 853 AD cases from multiply affected families and 171 Hispanic controls. The Case-Control and Enriched Case Study spans 24 cohorts provided by the Alzheimer’s Disease Genetics Consortium (ADGC) and the Cohorts for Heart and Aging Research in Genomic Epidemiology (CHARGE) Consortium.

Sequencing for these samples was conducted through three National Human Genome Research Institute (NHGRI) funded Large Scale Sequencing and Analysis Centers (LSACs): Baylor College of Medicine Human Genome Sequencing Center, the Broad Institute, the McDonnell Genome Institute at Washington University. The samples were sequenced on the Illumina HiSeq 2000/2500 platforms with 100bp paired-end reads. In the ADSP Discovery Case Control, 4586 samples were sequenced using the Illumina Rapid Capture Exome (ICE) kit and 6343 samples were sequenced using Roche Nimblegen’s VCRome v2.1 target capture kit. BAM files from hg37 build were sent to GCAD for processing on the VCPA1.1 pipeline. 10634 passed sequencing metrics and quality control.

snd10001- Alzheimer’s Disease Sequencing Project (ADSP) Extension

To further assess the genomes in multiply affected families, under funding provided by NHGRI, an additional 427 samples were whole genome sequenced. This included 107 additional samples from families studied under the Discovery Phase, 175 samples from 47 new families, and 145 Hispanic Controls. This portion of the study is called the Discovery Extension Phase. The Family Based Study spans seven cohorts provided by the Alzheimer’s Disease Genetics

Consortium (ADGC) and the Cohorts for Heart and Aging Research in Genomic Epidemiology (CHARGE) Consortium.

Under funding provided by NHGRI, an additional 3,000 subjects were whole genome sequenced. This included 1,466 cases and 1,534 controls. Of these 1,000 each of Non-Hispanic White (NHW), Caribbean Hispanic (CH), and African American (AA) descent were sequenced. Of these a total of 739 autopsy samples were sequenced [568 cases (500 NHW cases and 68 AA cases) and 171 controls (164 NHW and 7 AA)]. This Case-Control spans 5 cohorts provided by the Alzheimer's Disease Genetics Consortium (ADGC).

Sequencing for these samples was conducted through three National Human Genome Research Institute (NHGRI) funded Large Scale Sequencing and Analysis Centers (LSACs): Baylor College of Medicine Human Genome Sequencing Center, the Broad Institute, the McDonnell Genome Institute at Washington University. The samples were sequenced on the Illumina HiSeq X Ten platform with 150bp paired-end reads.

snd10002- Alzheimer's Disease Neuroimaging Initiative (ADNI)

ADNI is a global research study that actively supports the investigation and development of treatments that slow or stop the progression of AD. In this multisite longitudinal study, researchers at 63 sites in the US and Canada track the progression of AD in the human brain with clinical, imaging, genetic and biospecimen biomarkers through the process of normal aging, early mild cognitive impairment (EMCI), and late mild cognitive impairment (LMCI) to dementia or AD. Participants undergo a series of initial tests that are repeated at intervals over subsequent years, including a clinical evaluation, neuropsychological tests, genetic testing, lumbar puncture, and MRI and PET scans. The overall goal of ADNI is to validate biomarkers for use in Alzheimer' s disease clinical treatment trials. 1338 cases and 483 controls are included in this study across 4 phases.

Data used in the preparation of this article were obtained from the Alzheimer's Disease Neuroimaging Initiative (ADNI) database (adni.loni.usc.edu). The ADNI was launched in 2003 as a public-private partnership, led by Principal Investigator Michael W. Weiner, MD. The primary goal of ADNI has been to test whether serial magnetic resonance imaging

(MRI), positron emission tomography (PET), other biological markers, and clinical and neuropsychological assessment can be combined to measure the progression of mild cognitive impairment (MCI) and early Alzheimer's disease (AD). For up-to-date information, see www.adni-info.org.

snd10011-Accelerating Medicines Partnership – Alzheimer's Disease (AMP-AD)

AMP-AD samples from the ROSMAP, MayoRNAseq, and Mount Sinai Brain Bank cohorts were whole-genome sequenced at New York Genome Center on the HiSeqX machine. FASTQ files were sent to GCAD for processing on the VCPA1.1 pipeline. 1326 samples passed sequencing metrics and quality control.

snd10020-ADSP-Follow-up Study 1(FUS1)

The ADSP-FUS is a National Institute on Aging (NIA) initiative focused on identifying genetic risk and protective variants for late-onset Alzheimer Disease (LOAD). A concern in AD genetic studies is a lack of racial-ethnic diversity. The ADSP-FUS collects and sequences existing ethnically diverse and unique cohorts with clinical data to expand the utility of new discoveries for individuals from all populations.

ADSP FUS1 samples were whole-genome sequenced at USUHS either on the HiSeqX or NovaSeq machine. FASTQ files were sent to GCAD for processing on the VCPA1.1 pipeline. A total of 8,160 samples passed sequencing metrics and quality control.

This first release contains 3,250 AD cases, 4,149 cognitively normal individuals, 194 individuals with mild cognitive impairment, and 567 with unknown or other dementia from seven datasets (PR1066, ADC Autopsy, ADGC African American (release 1), HIHG Brain Bank, ADNI-WGS-2, APOE Extremes, and StEP AD).

snd10030-Alzheimer's Disease Genetics Consortium Texas Alzheimer's Research and Care Consortium (ADGC-TARCC)

The TARCC samples were sequenced at USUHS on the Novaseq machine. 1,018 samples were sequenced and FASTQ files were sent to GCAD for processing on the VCPA 1.1 pipeline. A total of 1,017 samples passed sequencing metrics and quality control checks.

snd10031-ADSP-Follow-up Study 2(FUS2)

The ADSP-FUS is a National Institute on Aging (NIA) initiative focused on identifying genetic risk and protective variants for late-onset Alzheimer Disease (LOAD). A concern in AD genetic studies is a lack of racial-ethnic diversity. The ADSP-FUS collects and sequences existing ethnically diverse and unique cohorts with clinical data to expand the utility of new discoveries for individuals from all populations.

ADSP FUS2 samples were whole-genome sequenced at either at the University of Miami on the HiSeqX or USUHS on the NovaSeq machine. 13228 samples were sequenced and FASTQ files were sent to GCAD for processing on the VCPA1.1 pipeline. A total of 12,621 samples passed sequencing metrics and quality control checks.

snd10032-Early-onset Alzheimer's Disease (EOAD1)

The EOAD samples were sequenced at USUHS on the NovaSeq machine. 3176 samples were sequenced and FASTQ files were sent to GCAD for processing on the VCPA 1.1 pipeline. A total of 3,132 samples passed sequencing metrics and quality control checks.

snd10033- The Diagnostic Assessment of Dementia for the Longitudinal Aging Study of India (LASI-DAD)

2,768 LASI-DAD respondents from 18 diverse ethno-linguistic and genographic groups across India who consented to the blood sample collection samples were sequenced at Medgenome on the HiSeqX machine. FASTQ files were sent to GCAD for processing on the VCPA 1.1 pipeline. A total of 2,686 samples passed sequencing metrics and quality control checks (including 6 technical replicates sequenced with ADSP rounds).

Clinical consensus diagnosis of dementia was achieved by using the Clinical Dementia Rating (CDR). At least three clinicians reviewed the case status and a second round of review by independent clinicians were conducted if the global ratings were different. For individuals without clinical consensus diagnoses, dementia status was predicted using a selected machine learning model, whose overall accuracy and agreement with the final consensus diagnoses

between the selected machine learning model and clinicians who participated in the clinical consensus diagnostic process were highest among all trained machine learning models.

Quality Control Filtering

The Genomic Center for Alzheimer Disease (GCAD) performs single nucleotide and insertion deletion variant calling for the ADSP. They deliver project level VCF files that include all variants called with flags indicating quality control (QC) metrics that can be used for filtering. For the current study the GCAD provided QC flags were used to identify and retain high quality variants within the ADSP VCFs. Specifically, for WGS data variants that did not receive a GATK pass, that were monomorphic across all samples, or had low call rate across all studies were excluded. Additionally, genotypes were set to missing for all individuals within a study if the variant had high mean depth or an ABhet ratio outside of 0.25 to 0.75. For WES data, the same filtering was implemented with the following modifications: genotypes were set to missing within a study if a low call rate was observed within that study and if variants were outside of the target region defined as the intersection of the target capture kits used by all contributing studies. Details of the GCAD provided QC flags can be found in the readme files at NIAGADS.

Race/Ethnicity Information

Reported race and ethnicity information in the phenotypic files from NIAGADS was combined to represent Hispanic and Non-Hispanic White, Hispanic and Non-Hispanic Black, Hispanic and Non-Hispanic Other/Unknown groups in the ADSP individuals. The other/unknown group includes Native Americans/Alaska Native, Asian, Native Hawaiian/Pacific Islander, other reported races, and unknown race.

Acknowledgement

Data for this study were prepared, archived, and distributed by the National Institute on Aging Alzheimer's Disease Data Storage Site (NIAGADS) at the University of Pennsylvania (U24-AG041689), funded by the National Institute on Aging.

Alzheimer's Disease Sequencing Project (sa000001) data:

The Alzheimer's Disease Sequencing Project (ADSP) is comprised of two Alzheimer's Disease (AD) genetics consortia and three National Human Genome Research Institute (NHGRI) funded Large Scale Sequencing and Analysis Centers (LSAC). The two AD genetics consortia are the Alzheimer's Disease Genetics Consortium (ADGC) funded by NIA (U01 AG032984), and the Cohorts for Heart and Aging Research in Genomic Epidemiology (CHARGE) funded by NIA (R01 AG033193), the National Heart, Lung, and Blood Institute (NHLBI), other National Institute of Health (NIH) institutes and other foreign governmental and non-governmental organizations. The Discovery Phase analysis of sequence data is supported through UF1AG047133 (to Drs. Schellenberg, Farrer, Pericak-Vance, Mayeux, and Haines); U01AG049505 to Dr. Seshadri; U01AG049506 to Dr. Boerwinkle; U01AG049507 to Dr. Wijsman; and U01AG049508 to Dr. Goate and the Discovery Extension Phase analysis is supported through U01AG052411 to Dr. Goate, U01AG052410 to Dr. Pericak-Vance and U01 AG052409 to Drs. Seshadri and Fornage.

Sequencing for the Follow Up Study (FUS) is supported through U01AG057659 (to Drs. PericakVance, Mayeux, and Vardarajan) and U01AG062943 (to Drs. Pericak-Vance and Mayeux). Data generation and harmonization in the Follow-up Phase is supported by U54AG052427 (to Drs. Schellenberg and Wang). The FUS Phase analysis of sequence data is supported through U01AG058589 (to Drs. Destefano, Boerwinkle, De Jager, Fornage, Seshadri, and Wijsman), U01AG058654 (to Drs. Haines, Bush, Farrer, Martin, and Pericak-Vance), U01AG058635 (to Dr. Goate), RF1AG058066 (to Drs. Haines, Pericak-Vance, and Scott), RF1AG057519 (to Drs. Farrer and Jun), R01AG048927 (to Dr. Farrer), and RF1AG054074 (to Drs. Pericak-Vance and Beecham).

The ADGC cohorts include: Adult Changes in Thought (ACT) (U01 AG006781, U19 AG066567), the Alzheimer's Disease Research Centers (ADRC) (P30 AG062429, P30

AG066468, P30 AG062421, P30 AG066509, P30 AG066514, P30 AG066530, P30 AG066507, P30 AG066444, P30 AG066518, P30 AG066512, P30 AG066462, P30 AG072979, P30 AG072972, P30 AG072976, P30 AG072975, P30 AG072978, P30 AG072977, P30 AG066519, P30 AG062677, P30 AG079280, P30 AG062422, P30 AG066511, P30 AG072946, P30 AG062715, P30 AG072973, P30 AG066506, P30 AG066508, P30 AG066515, P30 AG072947, P30 AG072931, P30 AG066546, P20 AG068024, P20 AG068053, P20 AG068077, P20 AG068082, P30 AG072958, P30 AG072959), the Chicago Health and Aging Project (CHAP) (R01 AG11101, RC4 AG039085, K23 AG030944), Indiana Memory and Aging Study (IMAS) (R01 AG019771), Indianapolis Ibadan (R01 AG009956, P30 AG010133), the Memory and Aging Project (MAP) (R01 AG17917), Mayo Clinic (MAYO) (R01 AG032990, U01 AG046139, R01 NS080820, RF1 AG051504, P50 AG016574), Mayo Parkinson's Disease controls (NS039764, NS071674, 5RC2HG005605), University of Miami (R01 AG027944, R01 AG028786, R01 AG019085, IIRG09133827, A2011048), the Multi-Institutional Research in Alzheimer's Genetic Epidemiology Study (MIRAGE) (R01 AG09029, R01 AG025259), the National Centralized Repository for Alzheimer's Disease and Related Dementias (NCRAD) (U24 AG021886), the National Institute on Aging Late Onset Alzheimer's Disease Family Study (NIA- LOAD) (U24 AG056270), the Religious Orders Study (ROS) (P30 AG10161, R01 AG15819), the Texas Alzheimer's Research and Care Consortium (TARCC) (funded by the Darrell K Royal Texas Alzheimer's Initiative), Vanderbilt University/Case Western Reserve University (VAN/CWRU) (R01 AG019757, R01 AG021547, R01 AG027944, R01 AG028786, P01 NS026630, and Alzheimer's Association), the Washington Heights-Inwood Columbia Aging Project (WHICAP) (RF1 AG054023), the University of Washington Families (VA Research Merit Grant, NIA: P50AG005136, R01AG041797, NINDS: R01NS069719), the Columbia University Hispanic Estudio Familiar de Influencia Genetica de Alzheimer (EFIGA) (RF1 AG015473), the University of Toronto (UT) (funded by Wellcome Trust, Medical Research Council, Canadian Institutes of Health Research), and Genetic Differences (GD) (R01 AG007584). The CHARGE cohorts are supported in part by National Heart, Lung, and Blood Institute (NHLBI) infrastructure grant HL105756 (Psaty), RC2HL102419 (Boerwinkle) and the neurology working group is supported by the National Institute on Aging (NIA) R01 grant AG033193.

The CHARGE cohorts participating in the ADSP include the following: Austrian Stroke Prevention Study (ASPS), ASPS-Family study, and the Prospective Dementia Registry-Austria (ASPS/PRODEM-Aus), the Atherosclerosis Risk in Communities (ARIC) Study, the Cardiovascular Health Study (CHS), the Erasmus Rucphen Family Study (ERF), the Framingham Heart Study (FHS), and the Rotterdam Study (RS). ASPS is funded by the Austrian Science Fond (FWF) grant number P20545-P05 and P13180 and the Medical University of Graz. The ASPS-Fam is funded by the Austrian Science Fund (FWF) project I904), the EU Joint Programme – Neurodegenerative Disease Research (JPND) in frame of the BRIDGET project (Austria, Ministry of Science) and the Medical University of Graz and the Steiermärkische Krankenanstalten Gesellschaft. PRODEM-Austria is supported by the Austrian Research Promotion agency (FFG) (Project No. 827462) and by the Austrian National Bank (Anniversary Fund, project 15435. ARIC research is carried out as a collaborative study supported by NHLBI contracts (HHSN268201100005C, HHSN268201100006C, HHSN268201100007C, HHSN268201100008C, HHSN268201100009C, HHSN268201100010C, HHSN268201100011C, and HHSN268201100012C). Neurocognitive data in ARIC is collected by U01 2U01HL096812, 2U01HL096814, 2U01HL096899, 2U01HL096902, 2U01HL096917 from the NIH (NHLBI, NINDS, NIA and NIDCD), and with previous brain MRI examinations funded by R01-HL70825 from the NHLBI. CHS research was supported by contracts HHSN268201200036C, HHSN268200800007C, N01HC55222, N01HC85079, N01HC85080, N01HC85081, N01HC85082, N01HC85083, N01HC85086, and grants U01HL080295 and U01HL130114 from the NHLBI with additional contribution from the National Institute of Neurological Disorders and Stroke (NINDS). Additional support was provided by R01AG023629, R01AG15928, and R01AG20098 from the NIA. FHS research is supported by NHLBI contracts N01-HC-25195 and HHSN268201500001I. This study was also supported by additional grants from the NIA (R01s AG054076, AG049607 and AG033040 and NINDS (R01 NS017950). The ERF study as a part of EUROSPAN (European Special Populations Research Network) was supported by European Commission FP6 STRP grant number 018947 (LSHG-CT-2006-01947) and also received funding from the European Community's Seventh Framework Programme (FP7/2007-2013)/grant agreement HEALTH-F4-2007-201413 by the European Commission under the programme "Quality of Life and Management of the Living Resources" of 5th Framework Programme (no. QLG2-CT-2002-

01254). High-throughput analysis of the ERF data was supported by a joint grant from the Netherlands Organization for Scientific Research and the Russian Foundation for Basic Research (NWO-RFBR 047.017.043). The Rotterdam Study is funded by Erasmus Medical Center and Erasmus University, Rotterdam, the Netherlands Organization for Health Research and Development (ZonMw), the Research Institute for Diseases in the Elderly (RIDE), the Ministry of Education, Culture and Science, the Ministry for Health, Welfare and Sports, the European Commission (DG XII), and the municipality of Rotterdam. Genetic data sets are also supported by the Netherlands Organization of Scientific Research NWO Investments (175.010.2005.011, 911-03-012), the Genetic Laboratory of the Department of Internal Medicine, Erasmus MC, the Research Institute for Diseases in the Elderly (014-93-015; RIDE2), and the Netherlands Genomics Initiative (NGI)/Netherlands Organization for Scientific Research (NWO) Netherlands Consortium for Healthy Aging (NCHA), project 050-060-810. All studies are grateful to their participants, faculty and staff. The content of these manuscripts is solely the responsibility of the authors and does not necessarily represent the official views of the National Institutes of Health or the U.S. Department of Health and Human Services.

The FUS cohorts include: the Alzheimer’s Disease Research Centers (ADRC) (P30 AG062429, P30 AG066468, P30 AG062421, P30 AG066509, P30 AG066514, P30 AG066530, P30 AG066507, P30 AG066444, P30 AG066518, P30 AG066512, P30 AG066462, P30 AG072979, P30 AG072972, P30 AG072976, P30 AG072975, P30 AG072978, P30 AG072977, P30 AG066519, P30 AG062677, P30 AG079280, P30 AG062422, P30 AG066511, P30 AG072946, P30 AG062715, P30 AG072973, P30 AG066506, P30 AG066508, P30 AG066515, P30 AG072947, P30 AG072931, P30 AG066546, P20 AG068024, P20 AG068053, P20 AG068077, P20 AG068082, P30 AG072958, P30 AG072959), Alzheimer’s Disease Neuroimaging Initiative (ADNI) (U19AG024904), Amish Protective Variant Study (RF1AG058066), Cache County Study (R01AG11380, R01AG031272, R01AG21136, RF1AG054052), Case Western Reserve University Brain Bank (CWRUBB) (P50AG008012), Case Western Reserve University Rapid Decline (CWRURD) (RF1AG058267, NU38CK000480), CubanAmerican Alzheimer’s Disease Initiative (CuAADI) (3U01AG052410), Estudio Familiar de Influencia Genetica en Alzheimer (EFIGA) (5R37AG015473, RF1AG015473, R56AG051876), Genetic and Environmental Risk Factors for Alzheimer Disease Among African Americans Study (GenerAAtions) (2R01AG09029, R01AG025259,

2R01AG048927), Gwangju Alzheimer and Related Dementias Study (GARD) (U01AG062602), Hillblom Aging Network (2014-A-004-NET, R01AG032289, R01AG048234), Hussman Institute for Human Genomics Brain Bank (HIHGBB) (R01AG027944, Alzheimer's Association "Identification of Rare Variants in Alzheimer Disease"), Ibadan Study of Aging (IBADAN) (5R01AG009956), Longevity Genes Project (LGP) and LonGenity (R01AG042188, R01AG044829, R01AG046949, R01AG057909, R01AG061155, P30AG038072), Mexican Health and Aging Study (MHAS) (R01AG018016), Multi-Institutional Research in Alzheimer's Genetic Epidemiology (MIRAGE) (2R01AG09029, R01AG025259, 2R01AG048927), Northern Manhattan Study (NOMAS) (R01NS29993), Peru Alzheimer's Disease Initiative (PeADI) (RF1AG054074), Puerto Rican 1066 (PR1066) (Wellcome Trust (GR066133/GR080002), European Research Council (340755)), Puerto Rican Alzheimer Disease Initiative (PRADI) (RF1AG054074), Reasons for Geographic and Racial Differences in Stroke (REGARDS) (U01NS041588), Research in African American Alzheimer Disease Initiative (REAAADI) (U01AG052410), the Religious Orders Study (ROS) (P30 AG10161, P30 AG72975, R01 AG15819, R01 AG42210), the RUSH Memory and Aging Project (MAP) (R01 AG017917, R01 AG42210Stanford Extreme Phenotypes in AD (R01AG060747), University of Miami Brain Endowment Bank (MBB), University of Miami/Case Western/North Carolina A&T African American (UM/CASE/NCAT) (U01AG052410, R01AG028786), Wisconsin Registry for Alzheimer's Prevention (WRAP) (R01AG027161 and R01AG054047), Mexico-Southern California Autosomal Dominant Alzheimer's Disease Consortium (R01AG069013), Center for Cognitive Neuroscience and Aging (R01AG047649), and the A4 Study (R01AG063689, U19AG010483 and U24AG057437).

The four LSACs are: the Human Genome Sequencing Center at the Baylor College of Medicine (U54 HG003273), the Broad Institute Genome Center (U54HG003067), The American Genome Center at the Uniformed Services University of the Health Sciences (U01AG057659), and the Washington University Genome Institute (U54HG003079). Genotyping and sequencing for the ADSP FUS is also conducted at John P. Hussman Institute for Human Genomics (HIHG) Center for Genome Technology (CGT).

Biological samples and associated phenotypic data used in primary data analyses were stored at Study Investigators institutions, and at the National Centralized Repository for Alzheimer's

Disease and Related Dementias (NCRAD, U24AG021886) at Indiana University funded by NIA. Associated Phenotypic Data used in primary and secondary data analyses were provided by Study Investigators, the NIA funded Alzheimer's Disease Centers (ADCs), and the National Alzheimer's Coordinating Center (NACC, U24AG072122) and the National Institute on Aging Genetics of Alzheimer's Disease Data Storage Site (NIAGADS, U24AG041689) at the University of Pennsylvania, funded by NIA. Harmonized phenotypes were provided by the ADSP Phenotype Harmonization Consortium (ADSP-PHC), funded by NIA (U24 AG074855, U01 AG068057 and R01 AG059716) and Ultrascale Machine Learning to Empower Discovery in Alzheimer's Disease Biobanks (AI4AD, U01 AG068057). This research was supported in part by the Intramural Research Program of the National Institutes of health, National Library of Medicine. Contributors to the Genetic Analysis Data included Study Investigators on projects that were individually funded by NIA, and other NIH institutes, and by private U.S. organizations, or foreign governmental or nongovernmental organizations.

The ADSP Phenotype Harmonization Consortium (ADSP-PHC) is funded by NIA (U24 AG074855, U01 AG068057 and R01 AG059716). The harmonized cohorts within the ADSP-PHC include: the Anti-Amyloid Treatment in Asymptomatic Alzheimer's study (A4 Study), a secondary prevention trial in preclinical Alzheimer's disease, aiming to slow cognitive decline associated with brain amyloid accumulation in clinically normal older individuals. The A4 Study is funded by a public-private-philanthropic partnership, including funding from the National Institutes of Health-National Institute on Aging, Eli Lilly and Company, Alzheimer's Association, Accelerating Medicines Partnership, GHR Foundation, an anonymous foundation and additional private donors, with in-kind support from Avid and Cogstate. The companion observational Longitudinal Evaluation of Amyloid Risk and Neurodegeneration (LEARN) Study is funded by the Alzheimer's Association and GHR Foundation. The A4 and LEARN Studies are led by Dr. Reisa Sperling at Brigham and Women's Hospital, Harvard Medical School and Dr. Paul Aisen at the Alzheimer's Therapeutic Research Institute (ATRI), University of Southern California. The A4 and LEARN Studies are coordinated by ATRI at the University of Southern California, and the data are made available through the Laboratory for Neuro Imaging at the University of Southern California. The participants screening for the A4 Study provided permission to share their de-identified data in order to advance the quest to find a successful treatment for Alzheimer's disease. We would like to acknowledge the dedication of all the

participants, the site personnel, and all of the partnership team members who continue to make the A4 and LEARN Studies possible. The complete A4 Study Team list is available on: a4study.org/a4-study-team.; the Adult Changes in Thought study (ACT), U01 AG006781, U19 AG066567; Alzheimer's Disease Neuroimaging Initiative (ADNI): Data collection and sharing for this project was funded by the Alzheimer's Disease Neuroimaging Initiative (ADNI) (National Institutes of Health Grant U01 AG024904) and DOD ADNI (Department of Defense award number W81XWH-12-2-0012). ADNI is funded by the National Institute on Aging, the National Institute of Biomedical Imaging and Bioengineering, and through generous contributions from the following: AbbVie, Alzheimer's Association; Alzheimer's Drug Discovery Foundation; Araclon Biotech; BioClinica, Inc.; Biogen; Bristol-Myers Squibb Company; CereSpir, Inc.; Cogstate; Eisai Inc.; Elan Pharmaceuticals, Inc.; Eli Lilly and Company; EuroImmun; F. Hoffmann-La Roche Ltd and its affiliated company Genentech, Inc.; Fujirebio; GE Healthcare; IXICO Ltd.;Janssen Alzheimer Immunotherapy Research & Development, LLC.; Johnson & Johnson Pharmaceutical Research & Development LLC.; Lumosity; Lundbeck; Merck & Co., Inc.;Meso Scale Diagnostics, LLC.; NeuroRx Research; Neurotrack Technologies; Novartis Pharmaceuticals Corporation; Pfizer Inc.; Piramal Imaging; Servier; Takeda Pharmaceutical Company; and Transition Therapeutics. The Canadian Institutes of Health Research is providing funds to support ADNI clinical sites in Canada. Private sector contributions are facilitated by the Foundation for the National Institutes of Health (www.fnih.org). The grantee organization is the Northern California Institute for Research and Education, and the study is coordinated by the Alzheimer's Therapeutic Research Institute at the University of Southern California. ADNI data are disseminated by the Laboratory for Neuro Imaging at the University of Southern California; Estudio Familiar de Influencia Genetica en Alzheimer (EFIGA): 5R37AG015473, RF1AG015473, R56AG051876; the Health & Aging Brain Study – Health Disparities (HABS-HD), supported by the National Institute on Aging of the National Institutes of Health under Award Numbers R01AG054073, R01AG058533, R01AG070862, P41EB015922, and U19AG078109; the Korean Brain Aging Study for the Early Diagnosis and Prediction of Alzheimer's disease (KBASE), which was supported by a grant from Ministry of Science, ICT and Future Planning (Grant No: NRF-2014M3C7A1046042); Memory & Aging Project at Knight Alzheimer's Disease Research Center (MAP at Knight ADRC): The Memory and Aging Project at the Knight-ADRC (Knight-

ADRC). This work was supported by the National Institutes of Health (NIH) grants R01AG064614, R01AG044546, RF1AG053303, RF1AG058501, U01AG058922 and R01AG064877 to Carlos Cruchaga. The recruitment and clinical characterization of research participants at Washington University was supported by NIH grants P30AG066444, P01AG03991, and P01AG026276. Data collection and sharing for this project was supported by NIH grants RF1AG054080, P30AG066462, R01AG064614 and U01AG052410. We thank the contributors who collected samples used in this study, as well as patients and their families, whose help and participation made this work possible. This work was supported by access to equipment made possible by the Hope Center for Neurological Disorders, the Neurogenomics and Informatics Center (NGI: https://neurogenomics.wustl.edu/) and the Departments of Neurology and Psychiatry at Washington University School of Medicine; National Alzheimer's Coordinating Center (NACC): The NACC database is funded by NIA/NIH Grant U24 AG072122. SCAN is a multi-institutional project that was funded as a U24 grant (AG067418) by the National Institute on Aging in May 2020. Data collected by SCAN and shared by NACC are contributed by the NIA-funded ADRCs as follows: P30 AG062429 (PI James Brewer, MD, PhD), P30 AG066468 (PI Oscar Lopez, MD), P30 AG062421 (PI Bradley Hyman, MD, PhD), P30 AG066509 (PI Thomas Grabowski, MD), P30 AG066514 (PI Mary Sano, PhD), P30 AG066530 (PI Helena Chui, MD), P30 AG066507 (PI Marilyn Albert, PhD), P30 AG066444 (PI John Morris, MD), P30 AG066518 (PI Jeffrey Kaye, MD), P30 AG066512 (PI Thomas Wisniewski, MD), P30 AG066462 (PI Scott Small, MD), P30 AG072979 (PI David Wolk, MD), P30 AG072972 (PI Charles DeCarli, MD), P30 AG072976 (PI Andrew Saykin, PsyD), P30 AG072975 (PI David Bennett, MD), P30 AG072978 (PI Neil Kowall, MD), P30 AG072977 (PI Robert Vassar, PhD), P30 AG066519 (PI Frank LaFerla, PhD), P30 AG062677 (PI Ronald Petersen, MD, PhD), P30 AG079280 (PI Eric Reiman, MD), P30 AG062422 (PI Gil Rabinovici, MD), P30 AG066511 (PI Allan Levey, MD, PhD), P30 AG072946 (PI Linda Van Eldik, PhD), P30 AG062715 (PI Sanjay Asthana, MD, FRCP), P30 AG072973 (PI Russell Swerdlow, MD), P30 AG066506 (PI Todd Golde, MD, PhD), P30 AG066508 (PI Stephen Strittmatter, MD, PhD), P30 AG066515 (PI Victor Henderson, MD, MS), P30 AG072947 (PI Suzanne Craft, PhD), P30 AG072931 (PI Henry Paulson, MD, PhD), P30 AG066546 (PI Sudha Seshadri, MD), P20 AG068024 (PI Erik Roberson, MD, PhD), P20 AG068053 (PI Justin Miller, PhD), P20 AG068077 (PI Gary Rosenberg, MD), P20 AG068082 (PI Angela Jefferson, PhD),

P30 AG072958 (PI Heather Whitson, MD), P30 AG072959 (PI James Leverenz, MD); National Institute on Aging Alzheimer's Disease Family Based Study (NIA-AD FBS): U24 AG056270; Religious Orders Study (ROS): P30AG10161,R01AG15819, R01AG42210; Memory and Aging Project (MAP - Rush): R01AG017917, R01AG42210; Minority Aging Research Study (MARS): R01AG22018, R01AG42210; the Texas Alzheimer's Research and Care Consortium (TARCC), funded by the Darrell K Royal Texas Alzheimer's Initiative, directed by the Texas Council on Alzheimer's Disease and Related Disorders; Washington Heights/Inwood Columbia Aging Project (WHICAP): RF1 AG054023;and Wisconsin Registry for Alzheimer's Prevention (WRAP): R01AG027161 and R01AG054047. Additional acknowledgments include the National Institute on Aging Genetics of Alzheimer's Disease Data Storage Site (NIAGADS, U24AG041689) at the University of Pennsylvania, funded by NIA.

Alzheimer's Disease Neuroimaging Initiative (sa000002) data:

Data collection and sharing for this project was funded by the Alzheimer's Disease Neuroimaging Initiative (ADNI) (National Institutes of Health Grant U01 AG024904) and DOD ADNI (Department of Defense award number W81XWH-12-2-0012). ADNI is funded by the National Institute on Aging, the National Institute of Biomedical Imaging and Bioengineering, and through generous contributions from the following: AbbVie, Alzheimer's Association; Alzheimer's Drug Discovery Foundation; Araclon Biotech; BioClinica, Inc.; Biogen; Bristol-Myers Squibb Company; CereSpir, Inc.; Cogstate; Eisai Inc.; Elan Pharmaceuticals, Inc.; Eli Lilly and Company; EuroImmun; F. Hoffmann-La Roche Ltd and its affiliated company Genentech, Inc.; Fujirebio; GE Healthcare; IXICO Ltd.; Janssen Alzheimer Immunotherapy Research & Development, LLC.; Johnson & Johnson Pharmaceutical Research & Development LLC.; Lumosity; Lundbeck; Merck & Co., Inc.; Meso Scale Diagnostics, LLC.; NeuroRx Research; Neurotrack Technologies; Novartis Pharmaceuticals Corporation; Pfizer Inc.; Piramal Imaging; Servier; Takeda Pharmaceutical Company; and Transition Therapeutics. The Canadian Institutes of Health Research is providing funds to support ADNI clinical sites in Canada. Private sector contributions are facilitated by the Foundation for the National Institutes of Health (www.fnih.org). The grantee organization is the Northern California Institute for Research and Education, and the study is coordinated by the Alzheimer's Therapeutic

Research Institute at the University of Southern California. ADNI data are disseminated by the Laboratory for Neuro Imaging at the University of Southern California.

Additional information to include in an acknowledgment statement can be found on the LONI site: https://adni.loni.usc.edu/wp-content/uploads/how_to_apply/ADNI_Data_Use_Agreement.pdf.

Alzheimer’s Disease Genetics Consortium (sa000003) data:

The Alzheimer’s Disease Genetics Consortium (ADGC) supported sample preparation, sequencing and data processing through NIA grant U01AG032984. Sequencing data generation and harmonization is supported by the Genome Center for Alzheimer’s Disease, U54AG052427, and data sharing is supported by NIAGADS, U24AG041689. Samples from the National Centralized Repository for Alzheimer’s Disease and Related Dementias (NCRAD), which receives government support under a cooperative agreement grant (U24 AG021886) awarded by the National Institute on Aging (NIA), were used in this study. We thank contributors who collected samples used in this study, as well as patients and their families, whose help and participation made this work possible.

ADGC-TARCC (snd10030) data:

This study was made possible by the Texas Alzheimer’s Research and Care Consortium (TARCC) funded by the state of Texas through the Texas Council on Alzheimer’s Disease and Related Disorders and the Darrell K Royal Texas Alzheimer’s Initiative.

Accelerating Medicines Partnership-Alzheimer’s Disease (AMP-AD) (sa000011) data:

Mayo RNAseq Study- Study data were provided by the following sources: The Mayo Clinic Alzheimer's Disease Genetic Studies, led by Dr. Nilufer Ertekin-Taner and Dr. Steven G. Younkin, Mayo Clinic, Jacksonville, FL using samples from the Mayo Clinic Study of Aging, the Mayo Clinic Alzheimer's Disease Research Center, and the Mayo Clinic Brain Bank. Data collection was supported through funding by NIA grants P50 AG016574, R01 AG032990, U01 AG046139, R01 AG018023, U01 AG006576, U01 AG006786, R01 AG025711, R01 AG017216, R01 AG003949, NINDS grant R01 NS080820, CurePSP Foundation, and support from Mayo Foundation. Study data includes samples collected through the Sun Health Research Institute

Brain and Body Donation Program of Sun City, Arizona. The Brain and Body Donation Program is supported by the National Institute of Neurological Disorders and Stroke (U24 NS072026 National Brain and Tissue Resource for Parkinson's Disease and Related Disorders), the National Institute on Aging (P30 AG19610 Arizona Alzheimer's Disease Core Center), the Arizona Department of Health Services (contract 211002, Arizona Alzheimer's Research Center), the Arizona Biomedical Research Commission (contracts 4001, 0011, 05-901 and 1001 to the Arizona Parkinson's Disease Consortium) and the Michael J. Fox Foundation for Parkinson's Research

ROSMAP- We are grateful to the participants in the Religious Order Study, the Memory and Aging Project. This work is supported by the US National Institutes of Health [U01 AG046152, R01 AG043617, R01 AG042210, R01 AG036042, R01 AG036836, R01 AG032990, R01 AG18023, RC2 AG036547, P50 AG016574, U01 ES017155, KL2 RR024151, K25 AG041906-01, R01 AG30146, P30 AG10161, R01 AG17917, R01 AG15819, K08 AG034290, P30 AG10161 and R01 AG11101.

Mount Sinai Brain Bank (MSBB)- This work was supported by the grants R01AG046170, RF1AG054014, RF1AG057440 and R01AG057907 from the NIH/National Institute on Aging (NIA). R01AG046170 is a component of the AMP-AD Target Discovery and Preclinical Validation Project. Brain tissue collection and characterization was supported by NIH HHSN271201300031C.

The Diagnostic Assessment of Dementia for the Longitudinal Aging Study of India (LASI-DAD) (sa000019) data:

The Longitudinal Aging Study in India, Diagnostic Assessment of Dementia data is sponsored by the National Institute on Aging (grant numbers R01AG051125 and U01AG065958) and is conducted by the University of Southern California.

Dissecting the Genomic Etiology of non-Mendelian Early-Onset Alzheimer Disease (EOAD) and Related Phenotypes (sa000023) data:

This work was supported by the National Institutes of Health (NIH) grant R01AG064614. The ADSP-FUS is supported by U01AG057659.

The National Institutes of Health, National Institute on Aging (NIH-NIA) supported this work through the following grants: ADGC, U01 AG032984, RC2 AG036528; samples from the National Centralized Repository for Alzheimer's Disease and Related Dementias (NCRAD), which receives government support under a cooperative agreement grant (U24 AG21886) awarded by the National Institute on Aging (NIA), were used in this study. Sequencing data generation and harmonization is supported by the Genome Center for Alzheimer's Disease, U54AG052427, and data sharing is supported by NIAGADS, U24AG041689. We thank contributors who collected samples used in this study, as well as patients and their families, whose help and participation made this work possible.

NIH grants supported enrollment and data collection for the individual studies including the Alzheimer's Disease Centers (ADC, P30 AG062429 (PI James Brewer, MD, PhD), P30 AG066468 (PI Oscar Lopez, MD), P30 AG062421 (PI Bradley Hyman, MD, PhD), P30 AG066509 (PI Thomas Grabowski, MD), P30 AG066514 (PI Mary Sano, PhD), P30 AG066530 (PI Helena Chui, MD), P30 AG066507 (PI Marilyn Albert, PhD), P30 AG066444 (PI John Morris, MD), P30 AG066518 (PI Jeffrey Kaye, MD), P30 AG066512 (PI Thomas Wisniewski, MD), P30 AG066462 (PI Scott Small, MD), P30 AG072979 (PI David Wolk, MD), P30 AG072972 (PI Charles DeCarli, MD), P30 AG072976 (PI Andrew Saykin, PsyD), P30 AG072975 (PI David Bennett, MD), P30 AG072978 (PI Neil Kowall, MD), P30 AG072977 (PI Robert Vassar, PhD), P30 AG066519 (PI Frank LaFerla, PhD), P30 AG062677 (PI Ronald Petersen, MD, PhD), P30 AG079280 (PI Eric Reiman, MD), P30 AG062422 (PI Gil Rabinovici, MD), P30 AG066511 (PI Allan Levey, MD, PhD), P30 AG072946 (PI Linda Van Eldik, PhD), P30 AG062715 (PI Sanjay Asthana, MD, FRCP), P30 AG072973 (PI Russell Swerdlow, MD), P30 AG066506 (PI Todd Golde, MD, PhD), P30 AG066508 (PI Stephen Strittmatter, MD, PhD), P30 AG066515 (PI Victor Henderson, MD, MS), P30 AG072947 (PI Suzanne Craft, PhD), P30 AG072931 (PI Henry Paulson, MD, PhD), P30 AG066546 (PI Sudha Seshadri, MD), P20 AG068024 (PI Erik Roberson, MD, PhD), P20 AG068053 (PI Justin Miller, PhD), P20 AG068077 (PI Gary Rosenberg, MD), P20 AG068082 (PI Angela Jefferson, PhD), P30 AG072958 (PI Heather Whitson, MD), P30 AG072959 (PI James Leverenz, MD). The Miami ascertainment and research were supported in part through: RF1AG054080, R01AG027944, R01AG019085, R01AG028786-02, RC2AG036528. The Columbia ascertainment and research were supported in part through: R37AG015473 and U24AG056270. The University of

Washington ascertainment and research were supported in part through R01AG044546, RF1AG053303, RF1AG058501, U01AG058922 and R01AG064877.

This work was supported in part by NIA grants U01AG058589 and U01AG068221. ADSP data for this study were prepared, archived, and distributed by the National Institute on Aging Alzheimer's Disease Data Storage Site (NIAGADS) at the University of Pennsylvania (U24-AG041689), funded by the National Institute on Aging (accession NG00067). The full acknowledgement statement for the ADSP, which includes funding information, can be found at: https://dss.niagads.org/datasets/ng00067/ .

Table S1. Descriptive Statistics in the Cognitively Healthy Samples.

	**Cognitively Healthy (n=1,784)**	**General Control (n=11,508)**
Female	1,151 (65%)	6,929 (60%)
Baseline Age (years)	82.2 (5.3)	71.7 (8.6)
Age Cognitively normal (years)	87 (2.5)	75 (6.2)
Age of Onset (years)	N/A	76.6 (6.1)

*Genotyped *APOE* allele counts were reported.

Table S2. Gene-based Test Results for Noncoding Variants in the Analyses.

Gene	CHR	Category	#SNV	cMAC	STAAR-O
**AD sample**					
HLA-F	6	Upstream	51	625	8.84×10^{-7}
**Cognitive healthy sample**					
MTERF2	12	UTR	140	1261	8.13×10^{-7}
STAMBP	2	Upstream	43	658	1.70×10^{-7}
PLXNB2	22	Upstream	11	24	8.03×10^{-9}
SULT1B1	4	Enhancer DHS	504	4163	5.61×10^{-7}
CARD11	7	Enhancer DHS	906	9615	3.42×10^{-8}
LINC01880	2	ncRNA	90	881	6.56×10^{-8}

*Showing genes with significant or suggestive association with ADRD or Cognitive healthy status.

**Abbreviations: Chromosome (Chr), single nucleotide variant (SNV), cumulative minor allele count (cMAC), untranslated region (UTR), DNase I hypersensitive site (DHS), noncoding RNA (ncRNA).

Table S3. Single Variant Test Results for *SSAN1* in the Cognitively Healthy Sample.

Chr	Position	Ref	Alt	N	MAC	β	SE	p-value
9	137188778	T	C	13292	1	-1.12	3.28	7.32E-01
9	137189129	A	G	13292	14	1.21	0.91	1.83E-01
9	137189131	A	C	13292	1	68.95	8.36	1.68E-16

Chr: Chromosome; MAC: minor allele count; SE: Standard Error.

Table S4. Single Variant Test Results for *ZNF200* in the AD Sample.

Chr	Position	REF	ALT	N	MAC	β	SE	p-value
16	3223922	G	T	23450	1	1.77	2.03	3.85E-01
16	3223936	G	A	23450	15	-0.19	0.59	7.53E-01
16	3224175	T	C	23451	1	-1.18	2.83	6.77E-01
16	3224196	A	G	23450	1	-1.24	2.58	6.31E-01
16	3224259	T	G	23452	3	0.05	1.25	9.67E-01
16	3224612	T	C	23450	3	0.243	1.32	8.56E-01
16	3233837	C	T	23451	1	47.66	6.98	8.46E-12

Chr: Chromosome; MAC: minor allele count; SE: Standard Error.

Table S5. Gene-based Test Results for Noncoding Variants in the Analyses after Implementing Modification #2.

Gene	CHR	Category	#SNV	cMAC	STAAR-O
**AD sample**					
HLA-F	6	Upstream	51	625	8.84×10^{-7}
**Cognitive healthy sample**					
MTERF2	12	UTR	140	1261	8.13x10-7
STAMBP	2	Upstream	43	658	1.70x10-7
PLXNB2	22	Upstream	11	24	5.35x10-9
SULT1B1	4	Enhancer DHS	504	4163	5.61x10-7
CARD11	7	Enhancer DHS	906	9615	3.42x10-8
LINC01880	2	ncRNA	90	881	6.56x10-8

*Showing genes with significant or suggestive association with ADRD or Cognitive healthy status.

**Abbreviations: Chromosome (Chr), single nucleotide variant (SNV), cumulative minor allele count (cMAC), untranslated region (UTR), DNase I hypersensitive site (DHS), noncoding RNA (ncRNA).

Figure S1. QQ-plots of the Gene-based Tests in the ADRD and Cognitively Healthy using the Original STAAR Framework.

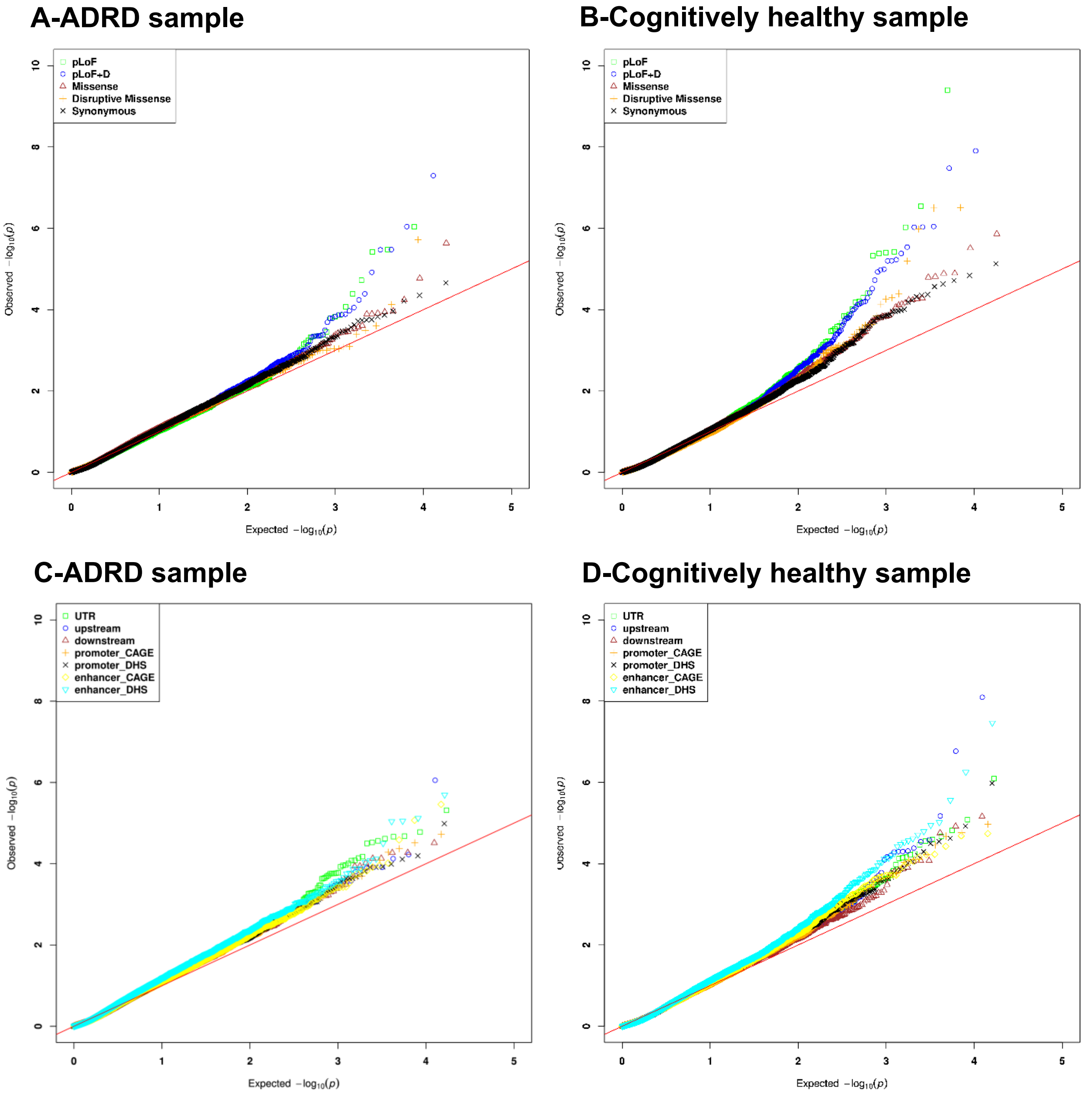

QQ-plots showing the distributions of gene-based test p-values from the original STAAR framework in the ADRD and cognitive healthy samples. A: gene-based test results for coding variants in the ADRD sample. B: gene-based test results for coding variant in the cognitive healthy sample. C: gene-based test results for noncoding variants in the ADRD sample. D: gene-based test results for noncoding variants in the cognitive healthy sample.

Figure S2. Manhattan Plots of the Gene-based Test Results in the ADRD and Cognitively Healthy Samples after implementing modification #1 to the STAAR framework.

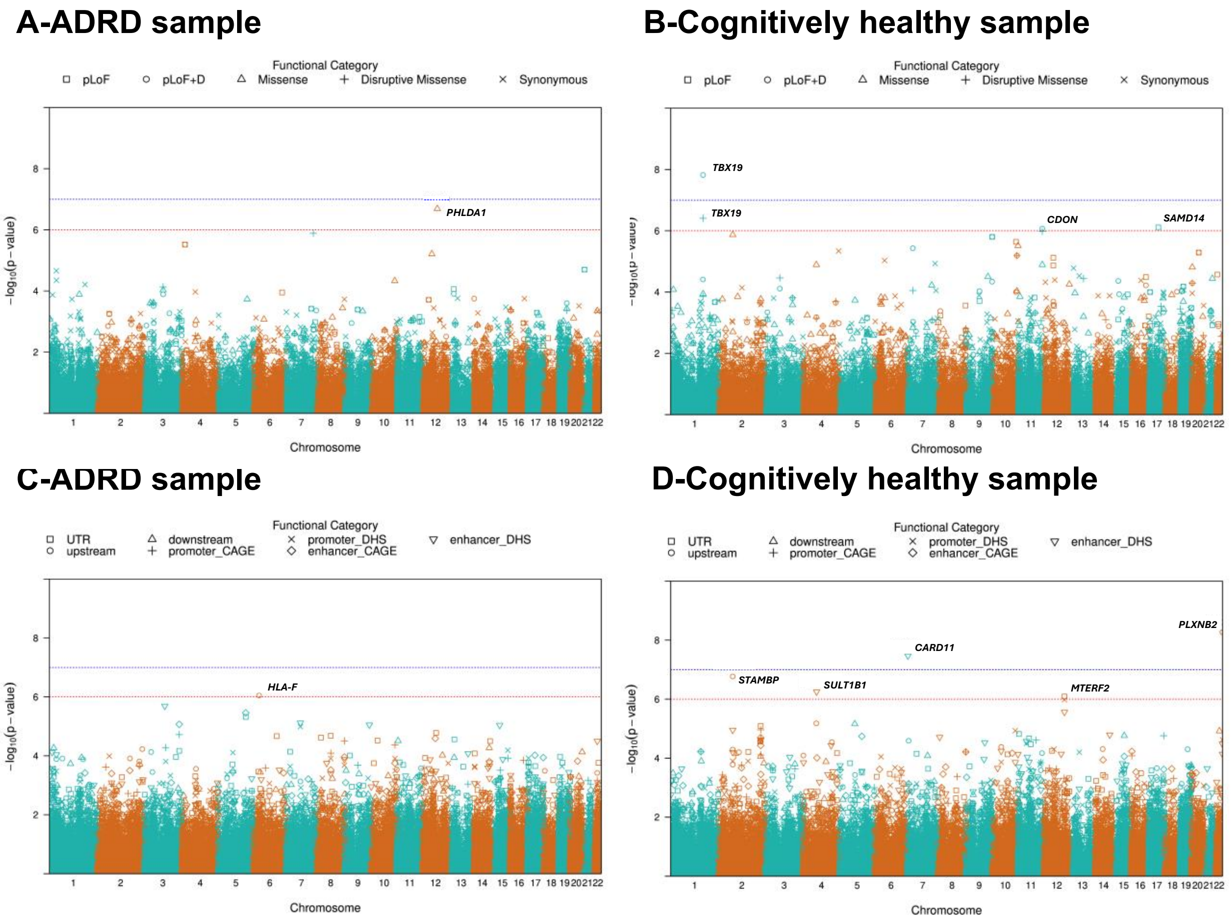

Manhattan plots showing gene-based test results for coding and noncoding variants after applying the modification #1 to the STAAR framework. The blue reference line represents the genome-wide significance threshold of $1x10^{-7}$ and the red reference line represents a suggestively significant threshold of 1x10-6. Specifically, panel A and C are gene-based test results for coding and noncoding variants in the ADRD sample, respectively. Panel B and D are gene-based test results for coding and noncoding variants in the cognitively healthy sample, respectively. Y-axis shows -log10(p-value) while x-axis represents the location of genes on chromosomes.

Figure S3. QQ-plots of the Gene-based Tests in the ADRD and Cognitively Healthy Samples after implementing modification #1 to the STAAR framework.

A-ADRD sample

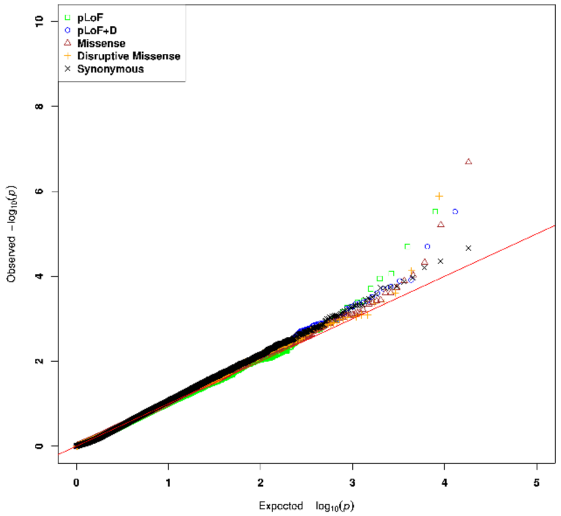

B-Cognitively healthy sample

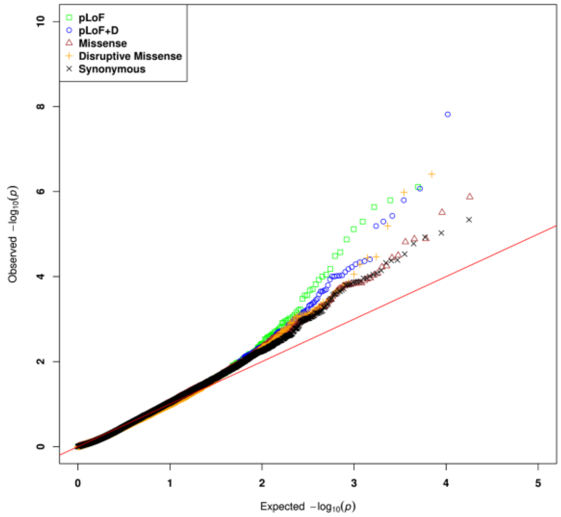

C-ADRD sample

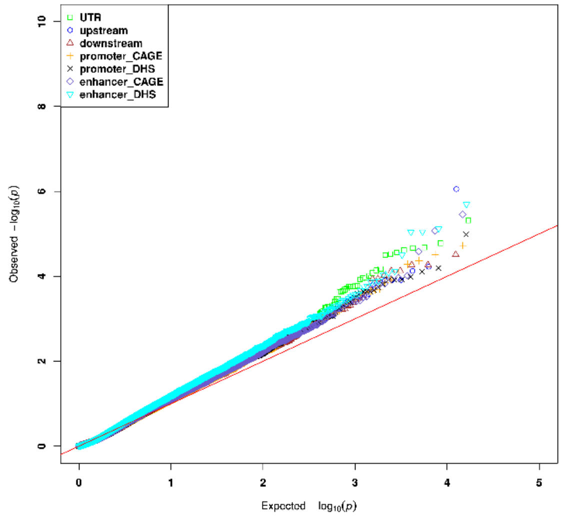

D-Cognitively healthy sample

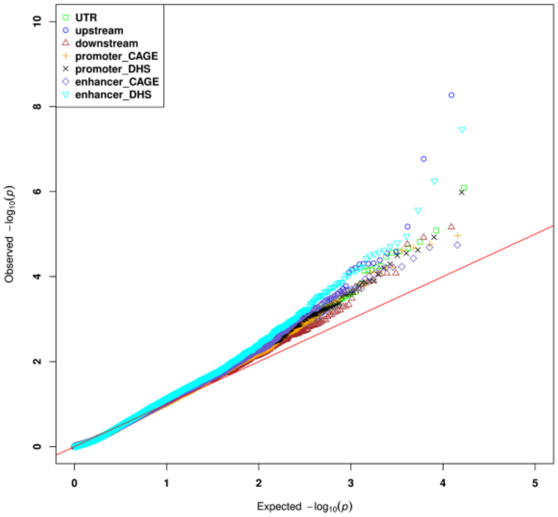

QQ-plots showing the distributions of gene-based test p-values after applying modification #1 to the STAAR framework in the ADRD and cognitive healthy samples. A: gene-based test results for coding variants in the ADRD sample. B: gene-based test results for coding variants in the cognitive healthy sample. C: gene-based test results for noncoding variants in the ADRD sample. D: gene-based test results for noncoding variants in the cognitive healthy sample.

Figure S4. Manhattan Plots of the Gene-based Tests of Noncoding Variants in the ADRD and Cognitively Healthy Samples before and after implementing modification #2.

A-ADRD sample

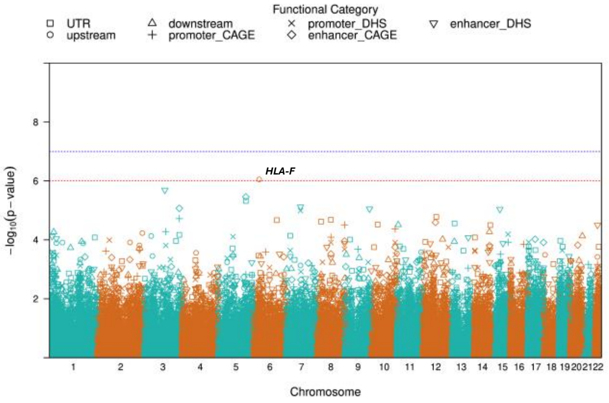

B- Cognitively healthy sample

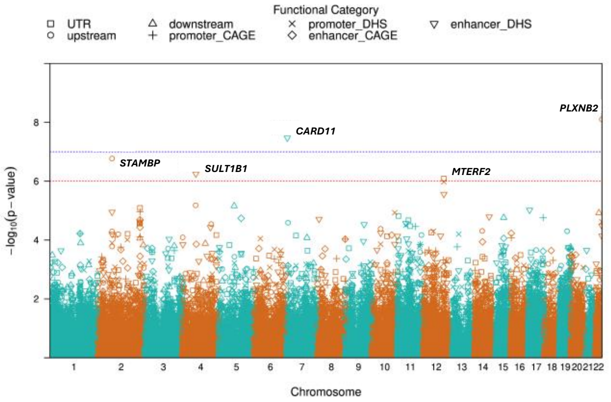

C-ADRD sample

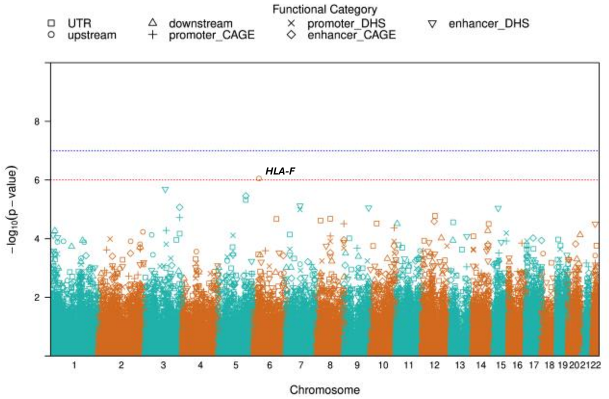

D- Cognitively healthy sample

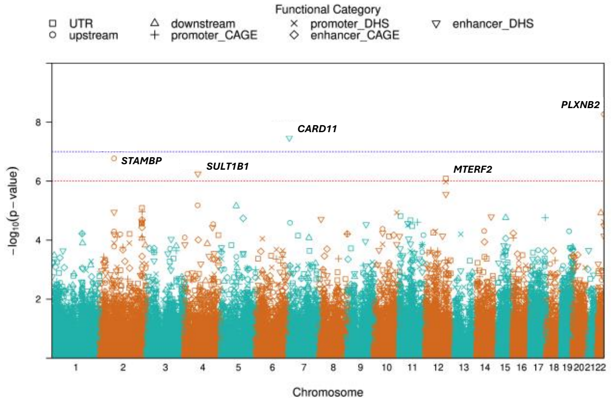

Manhattan plots showing gene-based test results for noncoding variants after applying modification #2 to the STAAR framework. The blue reference line represents the genome-wide significance threshold of $1x10^{-7}$ and the red reference line represents a suggestively significant threshold of $1x10^{-6}$. Specifically, panel A and C are gene-based test results before and after applying modifications #2 in the ADRD sample. Panel B and D are gene-based test results before and after applying modifications #2 in the cognitively healthy sample. Y-axis shows -log10(p-value) while x-axis represents the location of genes on chromosomes.

Figure S5. QQ-plots of the Gene-based Tests in the ADRD and Cognitively Healthy Samples after implementing modification #2 to the STAAR framework.

A-ADRD sample

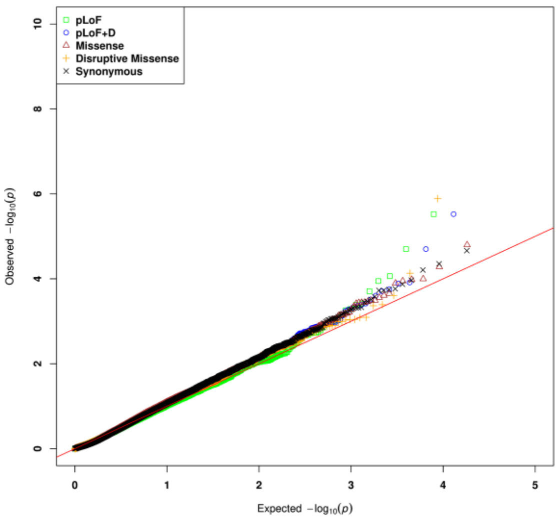

B-Cognitively healthy sample

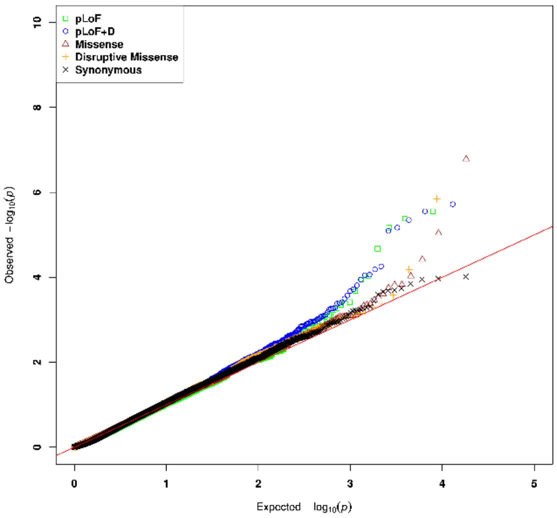

C-ADRD sample

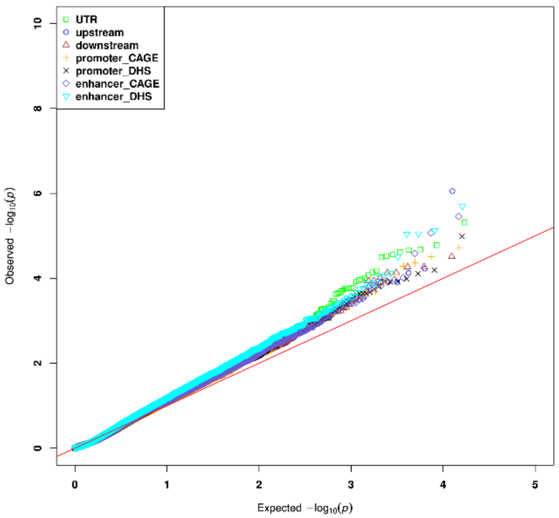

D-Cognitively healthy sample

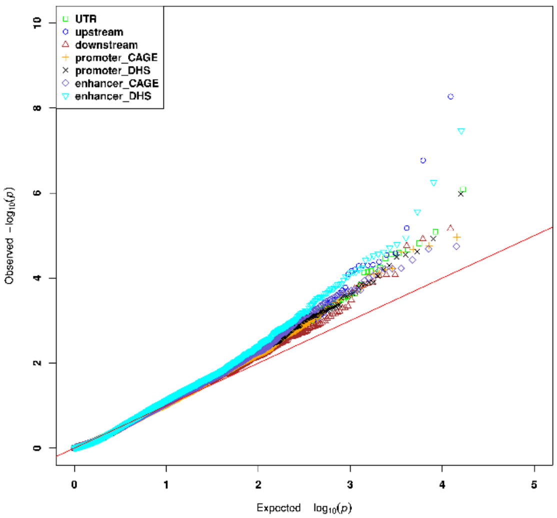

QQ-plots showing the distributions of gene-based test p-values after applying modification #2 to the STAAR framework in the ADRD and cognitive healthy samples. A: gene-based test results for coding variants in the ADRD sample. B: gene-based test results for coding variant in the cognitive healthy sample. C: gene-based test results for noncoding variants in the ADRD sample. D: gene-based test results for noncoding variants in the cognitive healthy sample.
